# Structural similarity, characterization of Poly Ethylene Glycol linkage and identification of product related variants in biosimilar pegfilgrastim

**DOI:** 10.1371/journal.pone.0212622

**Published:** 2019-03-13

**Authors:** Rakesh Shekhawat, Chintan Kumar Shah, Akash Patel, Sankaranarayanan Srinivasan, Poonam Kapoor, Suvaskumar Patel, Sharwan Kumar, Sandeep Sonar, Namrata More, Manasvi Joshi, Jatin Patel, Milan Vachhani, Bhargav Prasad Kodaganti, Upasana Choavatiya, Arabhi Pushpaja, Shubhangi Argade, Nidhi Nuwal, Manish Kumar, Sridevi Khambhampaty

**Affiliations:** 1 Analytical Development Laboratory, Intas Pharmaceuticals Ltd. (Biopharma Division), Ahmedabad, Gujarat, India; 2 Biocharacterization Development Laboratory, Intas Pharmaceuticals Ltd. (Biopharma Division), Ahmedabad, Gujarat, India; 3 CMC Technical writing team, Intas Pharmaceuticals Ltd. (Biopharma Division), Ahmedabad, Gujarat, India; University of Colorado Anschutz Medical Campus, UNITED STATES

## Abstract

The approval of biosimilars requires demonstration of biosimilarity, which rests on the base of thorough analytical characterization of the biosimilar product. In addition to demonstration of biosimilarity, the product related impurities need to be thoroughly characterized and controlled at minimal levels. Pegylation of peptides and proteins creates significant challenges for detailed structural characterization, such as PEG (Poly Ethylene Glycol) heterogeneity, site of addition and number of attached pegylated moieties. A combination of several methods including circular dichroism, FTIR (Fourier-transform Infrared Spectroscopy), fluorescence spectroscopy, DSC (Differential Scanning Calorimetry), 1D and 2D NMR (Nuclear Magnetic Resonance), Edman degradation and peptide mapping by LC-MS (Liquid Chromatography Mass Spectrometry) were used for characterization of N-terminally pegylated filgrastim. Product related impurities such as oxidized, reduced, deamidated, dipegylated variants and monopegylated positional isomers have been characterized in detail using various HPLC (High Performance Liquid Chromatography) based methods and LC-MS techniques. The functional characterization in terms of receptor binding and cell proliferation assay was conducted for the similarity assessment and the potential impact of the product variants on the *in vitro* biological activity has also been assessed. In summary, this study presents, for the first time, a detailed structural and molecular level characterization of a biosimilar pegfilgrastim providing a strong base for the demonstration of overall biosimilarity of the product with the innovator product.

## Introduction

A biosimilar product is defined as the biological product which is highly similar to the reference product not withstanding minor differences in clinically inactive components and that there are no clinically meaningful differences between the biological product and the reference product in terms of the safety, purity and potency of the product [1]. EMA guidance suggests that demonstration of similarity of a biological drug to the reference medicinal product in terms of pharmaceutical quality, structure, functional activity, efficacy, immunogenicity profile and safety based on the comparability exercise which is entailed to be established [2]. The demonstration of biosimilarity therefore involves extensive physicochemical and biological characterization of the product in comparison to the innovator product followed by clinical studies required to demonstrate complete biosimilarity. Intas has successfully developed a biosimilar filgrastim (Grastofil/Accofil) which was approved in EU in 2015. The quality of Intas Filgrastim has been compared in independent studies [3, 4] and found to be highly similar to the innovator product, with very low levels of product variants. Subsequently, Intas has developed a pegylated, long acting version of filgrastim. A limited biosimilarity assessment of this product was demonstrated in comparison to the US sourced Neulasta [5]. The biosimilar pegfilgrastim developed by Intas, under the brand name Pelgraz, is one of the first pegfilgrastim biosimilar to be approved for marketing authorization in EU. (http://www.ema.europa.eu/ema/pages/includes/document/open_document.jsp?webContentId=WC500252682). A comprehensive assessment of the Intas product in comparison to both EU and US sourced innovator products is discussed in this research article.

The production of proteins and peptides as efficient therapeutic drugs is employed for the treatment of many pathophysiological circumstances [6, 7]. Minor structural differences can considerably affect a protein’s safety and effectiveness, so it is important to evaluate these differences. Moreover, *in vivo*, cellular proteases may degrade the proteins and /or proteins can be rapidly excreted by kidneys, resulting into a short circulating half-life that reduces their therapeutic efficacy [6, 8]. To conquer these problems, several strategies have been explored including chemical modification with PEG (Pegylation). The covalent attachment of PEG is a well-established approach to improve protein stability and solubility, to reduce renal clearance and proteolytic degradation, and to decrease immunogenicity and antigenicity, all of which contribute to an improved clinical efficacy and safety profile. Due to its flexibility, hydrophilicity, variable size, and low toxicity, pegylation is of interest for extending protein half-life [6, 8–10]. Many pegylated biopharmaceuticals have been used in the market such as pegylated form of adenosine deaminase for treatment of severe combined immunodeficiency (SCID), pegylated Interferon-α2b and Interferon- α2a for treating Hepatitis B and C, and pegylated L-asparginase for treatment of certain types of leukemia [6, 8–10].

There are significant challenges related to the development of commercially viable pegylated protein product. The quality of a pegylated conjugate depends on the qualities of the PEG used for conjugation. One important challenge is–attachment of the PEG moiety at a specific site in a protein. Presence of impurities, distribution of molecular weight of the polymer and choice of reactive group chemistry will affect the structure and quality of the final pegylated protein [11, 12].

To manufacture pegfilgrastim, a 20 kDa monomethoxypolyethylene glycol (mPEG) molecule is covalently bound to the N-terminal methionyl residue of filgrastim using 20 kDa monomethoxy polyethylene glycol propionaldehyde (mPEG-PAL) as critical intermediate [9]. Recombinant human G-CSF (Granulocyte-Colony Stimulating Factor; rhG-CSF; INN filgrastim), a 175 amino acid long non-glycosylated polypeptide, expressed in *E*. *coli* was one of the first biopharmaceuticals to be commercialized (Neupogen; Amgen Inc.). Its additional N-terminal Met residue is not found in the native endogenous human protein. The structure of filgrastim consists of four α-helices arranged in an up–up, down–down conuration with a long loop connecting helices one and two and another loop between helices three and four. It contains two disulfide bonds (C37–C43 and C65–C75) and a single free cysteine residue (C18) [13]. G-CSF is a hematopoietic growth factor and cytokine that stimulates the production of neutrophils and affects neutrophil progenitor proliferation, differentiation, and cell functional activation [14]. Its recombinant form, filgrastim is used primarily to reduce incidence and duration of severe neutropenia and its associated complications in cancer patients that have received a chemotherapy regimen [15]. Pegylation increases the size of filgrastim so that it becomes too large for renal clearance and the clearance of the molecule is also dominated by a self-regulating mechanism, based on receptor mediated clearance. Due to its long half-life, pegfilgrastim requires only once-per-cycle administration for the management of chemotherapy-induced neutropenia, thus resulting in significant ease for patients.

During pegylation, mPEG-PAL preferentially reacts with N-terminal amino group of filgrastim forming a Schiff base in the first condensation step. This upon reduction with sodium cyanoborohydride forms a secondary amine bond between protein and polyethylene glycol to form pegylated filgrastim or pegfilgrastim [9]. Sodium cyanoborohydride was reported to be used for manufacture of Reference Medicinal Product, Neulasta [16].

The pegylated proteins need to be thoroughly characterized for provision of a safe and efficient drug and to meet the regulatory criteria for human use. The biosimilar pegylated filgrastim is developed, similar to the innovator product (Neulasta), by N-terminal pegylation of filgrastim using mPEG-PAL. The pegylation reaction is designed for N-terminal pegylation, however pegylation at the Lys side chains is also possible. The purification process is designed to remove variants arising from the pegylation process such as dipegylated variants and also isomers of monopegylated filgrastim. However, it is important to characterize all the residual impurities in the final purified drug product, to assess their impact on product efficacy and stability.

Herein, we demonstrate a high level of structural similarity of biosimilar pegfilgrastim with innovator pegfilgrastim using different analytical techniques for primary, higher order structure and functional assays. Subsequently, product related impurities (oxidized, deamidated and reduced variants of pegfilgrastim) and product variants generated during the process (dipegylated pegfilgrastim, Lys variants) of pegylation were isolated, purified and characterized by LC-MS (Liquid Chromatography–Mass Spectrometry) and LC-MS/MS. To ensure purity, the classical techniques, RP-HPLC (Reversed-phase High Performance Liquid Chromatography), SE-HPLC (Size Exclusion High Performance Liquid Chromatography) and CEx-HPLC (Cation Exchange High Performance Liquid Chromatography) were carried out. NMR technique was applied for identification of PEG conjugation site. The *in vitro* cell proliferation assay was employed to assess the potential impact of the variants on the function. Overall, the biosimilar pegfilgrastim developed by Intas has very low product variant levels in comparison to innovator product and these were thoroughly characterized.

## Materials and methods

### Materials

Acetonitrile (LC-MS grade), water (LC-MS grade) were procured from J T Baker. Formic acid (LC-MS grade) and tris (2-carboxyethyl) phosphine hydrochloride (TCEP.HCl) were procured from Thermo Scientific. Triethylamine (≥99.0%) was procured from Spectrochem. Ammonium bicarbonate (≥99.0%), tBHP (Tert-Butyl Hydroperoxide) (70%W/V), dithiothreitol (DTT) (≥99.0%), di-potassium hydrogen phosphate were procured from Sigma- Aldrich (Darmstadt, Germany). Sorbitol, sodium hydroxide, potassium di-hydrogen phosphate and glacial acetic acid were procured from Merck (GmBH). Endoprotease Glu-C and chymotrypsin (sequencing grade) were procured from Roche. Guanidine hydrochloride was procured from Nigu, USA. Ultrapure water (18.2 MΩ.cm at 25°C) was produced in house using the Milli-Q System (Millipore Corporation, Billerica, USA). The pegfilgrastim (10.0 mg mL^−1^ solution in 10 mmol L^−1^ acetate, 50 mg mL^−1^ sorbitol, pH (3.8–4.2) was produced by Intas Pharmaceutical Ltd—Biopharma division (Ahmedabad, India). The formulation buffer composition of pegfilgrastim drug product (10 mg mL^−1^) is 10 mmol L^−1^ acetate, 50 mg mL^−1^ sorbitol, 0.004 mg/mL polysorbate -20, pH (3.8–4.2). The pegfilgrastim product produced at Intas Pharmaceutical Ltd—Biopharma division is henceforth referred as INTP5 or pegylated filgrastim biosimilar throughout the study. Neulasta (Amgen Inc., USA) lots were procured from the US (United States), EU (European Union) and AUS (Australia) market is henceforth referred as originator product. For NMR studies, INTP5 lots are referred as I1 –I3, US lots as U1-U3 and EU lots as E1-E3. When referencing both originator and biosimilar products, the term pegfilgrastim is also used.

### Methods

#### N-Terminal sequencing

The samples were desalted using Zeba spin desalting columns from Thermo, samples were loaded on a PVDF membrane and analyzed using automated Edman degradation process using protein sequencer PPSQ-51A from Shimadzu.

#### Peptide mapping and amino acid sequencing using LC-MS (endoprotease Glu-C)

The samples were digested in native condition using endoprotease Glu-C followed by denaturation using guanidine hydrochloride. Further, one set of sample was reduced using DTT and one set of sample was kept in non-reduced condition. The digested peptides were then separated on a 1.7 μm, 2.1*100 mm BEH C18 column from Waters over a linear gradient of 0.95% B changes in 60 min at 0.2 mL/min flow rate with mobile phases containing 0.1% formic acid in water (mobile phase A) and 0.1% formic acid in acetonitrile (mobile phase B) and analyzed using mass spectrometer (Triple TOF 6600 or Triple TOF 4600 AB Sciex, USA). Capillary voltage of +5.5 kV, de-clustering potential 100 V, collision energy 10 V, mass range of m/z 100 to 2000 Da (for MS and MS/MS) and source temperature of 450°C were applied during analysis.

#### Circular dichroism

Far UV CD analysis was performed on a JASCO J-815 CD spectropolarimeter at 25°C. Samples were diluted in formulation buffer to a final concentration of 0.3 mg/mL, and spectra collected from 195 nm to 260 nm using a 0.1 cm path length cell. Blank correction of all the spectra was performed and the mean residue ellipticity, (deg cm^2^ dmol^-1^), was calculated according to the equation [Θ] = (100 x θ)/(c x l x N), where θ is the measured ellipticity (mdeg), c is the sample concentration (mM), l is the path length (cm), and N is the number of amino acids.

#### FTIR

Measurements were performed with an ATR (Attenuated Total Reflection) attachment on a IdentifyIR (Smiths Detection, Danbury CT, United States) system. The IR spectra were collected between 1900 cm^-1^ and 800 cm^-1^ with a 16-wavenumber resolution at a concentration of 10 mg/mL. Spectra were the average of 256 scans. A baseline correction was performed between each measurement.

#### Intrinsic fluorescence

All samples were diluted to a final concentration of 1 mg/mL in 0.9% NaCl. Steady-state fluorescence emission spectra were recorded with a spectrofluorometer FluoroMax-1 at 25°C using two excitation wavelengths: 271 nm and 291 nm. For excitation at 271 nm, the spectra were recorded between 280 nm and 450 nm using excitation slit of 1 mm and emission slit of 0.75 mm. For excitation at 291 nm, the spectra were recorded between 295 nm and 450 nm using excitation slit of 1 mm and emission slit of 1 mm. For both excitation wavelengths, the cuvette was placed in position l/s; the spectra were monitored with an integration time of 0.05 s, an increment of 1 nm and the mode S/R.

#### NMR spectroscopy

The samples at a concentration of 10 mg/mL were supplemented with D_2_O to 10% v/v and pipetted into a 5 mm NMR tube. 1D ^1^H spectra followed by 2D ^1^H-^13^C heteronuclear single quantum coherence (HSQC) NMR spectra were recorded at 298 K on a Bruker DRX800 equipped with a cryoprobe. Analysis of purified pegylated N-terminal peptide was carried out to provide an assessment of the linkage between PEG/Linker and the N-terminal Met residue (to the extent possible). Each of samples were reduced, carboxymethylated and digested with trypsin. The resultant peptides were separated using reversed phase HPLC. A fraction containing N-terminal pegylated peptide (PEG-MTPLGPASSLPQSFLLK) was collected, lyophilized and subjected to 1D and 2D ^1^H-^13^C NMR analysis. Collection of N-terminal pegylated peptide was performed from multiple injections to collect approximately 1 mg of pegylated peptide material. Natural abundance 2D ^1^H-^13^C HSQC (Heteronuclear Single Quantum Coherence) combined with 2D ^1^H-^13^C HMBC (Heteronuclear multiple bond correlation) spectra provides information of the N-terminal Met side chain. Samples were dissolved in 0.6 mL of 100% D_2_O and pipetted into a 5 mm NMR tube. 1D ^1^H spectra followed by 2D ^1^H-^13^C heteronuclear single quantum coherence (HSQC) and 2D ^1^H-^13^C HMBC (Heteronuclear multiple bond correlation) NMR spectra for the peptide samples were recorded at 298 K on a Bruker DRX800 equipped with a cryoprobe.

#### Differential scanning calorimetry (DSC)

The samples were diluted to a final concentration of 0.6 mg/mL in formulation buffer. Thermograms were recorded on a MicroCal VP-DSC at a scan rate of 60°C / h in a temperature range of 20°C to 90°C with a filtering period of 16 seconds. Data was analyzed using Origin software. Thermograms were corrected by subtraction of blank/buffer scan. The corrected thermograms were normalized for protein concentration. Peak integration was done by using non two state model.

#### Bioassay (*in vitro* cell proliferation assay)

The *in vitro* bioassay to determine the relative potency of pegfilgrastim is a cell proliferation assay. It utilizes the ability of murine myeloblastic cell line, M-NFS-60, to proliferate in the presence of pegfilgrastim in a dose dependent manner. M-NFS-60 cells were purchased from ATCC (CRL 1838) and adapted to grow in presence of GCSF. GCSF adapted M-NFS-60 cells (35,000 cells/well) were incubated with varying concentrations of pegfilgrastim reference solution or the test solution (0.006 ng/mL to 1.333 ng/mL). After a timed incubation of 44 ± 1.5 h, MTS (3-(4, 5-Dimethylthiazol-2-yl)-5-(3-carboxymethoxyphenyl)-2-(4-sulfophenyl)-2H-tetrazolium) and an electron coupling reagent PMS (Phenazine Methosulfate) were added to the plates. The dehydrogenase enzyme in viable cells reduces MTS into a colored formazan product that is soluble in tissue culture medium and can be measured spectrophotometrically. The readout was taken after an incubation of 4 h ± 15 min. Potency of each sample was assessed in three independent assays relative to an internal reference standard which was assigned a potency of 100%, based on the establishment data and geometric mean of the same is reported.

#### Flow cytometry based receptor binding assay

The receptor binding ability of pegfilgrastim to receptors on human granulocytes was assessed using FACSCalibur (BD Bioscience) flow cytometer. The assay is a competitive binding assay in which different concentration of unlabeled pegfilgrastim (0.06 ng/mL to 5000 ng/mL) competes with fixed concentration of biotin labeled GCSF to bind with GCSF receptors present on granulocytes (obtained by gating of normal human blood). The binding is measured using Phycoerythin labeled streptavidin. The fluorescence signal is inversely proportional to the concentration of pegfilgrastim. Binding activity of each sample was assessed in three independent assays relative to an internal reference standard and the geometric mean of the same is reported.

#### Binding kinetics analysis by SPR

Binding kinetic analysis was performed using surface plasma resonance (SPR) using Biacore 3000 or T200. Recombinant G-CSF receptor/Fc (Immunoglobulin constant region) chimera was captured on a pre-immobilized anti-human Fc antibody on a Biacore CM5 chip (GE Healthcare). The pegfilgrastim analyte solution (25 ng/mL to 400 ng/mL) is then passed over the chip and binding interactions can be measured. Data were fit with the 1:1 Langmuir global fit binding model. The association rate constant (k_a_), the dissociation rate constant (k_d_) and the equilibrium dissociation constant (K_D_) were measured. Each sample was tested in triplicate and average of the same is reported.

#### SDS-PAGE

Non- reducing SDS-PAGE with silver staining was performed using (4–20) % pre-cast gradient gel from Invitrogen. 0.015 μg (0.15%), 0.050 μg (0.5%), 0.15 μg (1.5%) and 10.00 μg (100%) of pegfilgrastim and filgrastim were loaded as reference standard. Amount of sample loaded was either 1.00 μg or 2.00 μg.

#### Generation of impurities

Oxidized impurities—INTP5 was forced oxidized using ~150 mM tBHP by incubating at 25°C for 24 hours.

Deamidated impurities—The deamidation in INTP5 was induced by pH stress using 20 mM glycine-HCl pH 2.0 by incubating at 25°C for ~15 days.

Higher molecular weight (HMW) impurities–The samples were vortexed for either 5 minutes or 24 hours.

#### Impurity peak collection, purification and enrichment

The pegfilgrastim impurity peaks appearing in various techniques (CEx-HPLC, RP-HPLC and SE-HPLC), either control or force degraded samples, were collected separately by manual impurity collection from multiple injections of the sample I2 (INTP5). Further the impurities were purified, concentrated and buffer exchanged to the formulation buffer (10 mmol L^−1^ acetate, 50 mg mL^−1^ sorbitol, pH 3.8−4.2) using centrifugal filter (Amikon ultra Millipore, Merck GmBH). All the impurity collection experiments were carried out using Agilent 1200 series HPLC. The details of techniques (CEx-HPLC, RP-HPLC and SE-HPLC) are mentioned below:

- **CEx-HPLC.** The separation of charge variant impurities was carried out on a 10 μm, 75 × 7.5 mm TSKgel SP-5PW column from TOSOH over a linear gradient of 4.0% B change in 15 min with mobile phases containing 10 mM sodium acetate pH 5.4 (mobile phase A) and 10 mM sodium acetate with 100 mM NaCl (mobile phase B).
- **RP-HPLC.** The separation of impurity based on hydrophobicity was carried out on a 5 μm, 300 Å, 250 × 4.6 mm Jupiter C18 column from Phenomenex over a linear gradient of 0.5% B change in 18 min with mobile phases containing 0.1% TFA in H_2_O (solvent A) and 0.1% TFA Trifluoroacetic Acid) in MeCN (solvent B). Column temperature was set at 60°C.
- **SEC-HPLC**. The low and high molecular weight impurities were analyzed on SEC-MALS. Separation was carried out on a 5 μm, 250 Å, 300 × 7.8 mm TSKgel G3000 SWXL hydrophilic silica gel column from TOSOH with mobile phase containing phosphate buffer with 100 mM NaCl and 10% ethanol. Column temperature was set at 25°C. INTP5 product corresponding to 36 μg of protein was loaded onto the column and further analyzed on MALS (Multiangle Light Scattering).

#### Mass spectrometry

Each collected impurity sample was digested using chymotrypsin and reduced using TCEP. HCl. The digested peptides were then separated on a 1.7 μm, 2.1*150 mm peptide CSH C18 column from Waters over a linear gradient of 0.23% B changes in 130 min at 0.2 mL/min flow rate with mobile phases containing 0.1% formic acid in water (mobile phase A) and 0.1% formic acid in acetonitrile (mobile phase B) and analyzed using mass spectrometer. Capillary voltage of +5.5 kV, de-clustering potential 100 V, collision energy 10 V, mass range of m/z 200 to 2000 Da for MS and 100 to 2000 Da for MS/MS and source temperature of 450°C were applied during analysis. Moreover, the collected impurity samples for determination of oxidation were digested using endoprotease Glu-C. The procedure followed for Glu-C is same as mentioned in primary structure analysis- peptide mapping. The mass spectra was obtained using information dependent analysis (IDA) mode in which one survey scan to identify 10 highest intensity peptides followed by high energy collision of these peptides to obtain MS/MS spectra was deployed to identify and confirm peptide sequence in impurity and Control (non-treated) sample. Spectra recorded with Analyst TF 1.7 were analyzed for the differences with Control sample using PeakView 2.2 (data analysis software) from AB Sciex.

## Results and discussion

### Structural similarity assessment

Similarity at primary structure level was established using N-terminal sequencing by Edman degradation process and Peptide mapping by MS and MS/MS.

The results of N-terminal sequencing provided in Table A in S1 Appendix shows that the INTP5, EU sourced Neulasta and US sourced Neulasta contain the same N-terminal amino acid sequence up to the first 20 residues. This sequence is the same as that for Filgrastim [17]. Furthermore, the first N-terminal amino acid residue is not detected by N-terminal sequencer indicating the pegylation of N-terminus in all three samples. Therefore, N-terminus is identical for all products tested and consistent with the expected amino acid sequence for a pegfilgrastim molecule.

**Fig 1:**
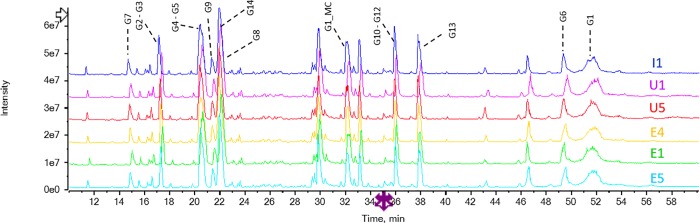
TIC stacked profile of INTP5 (Sample I1), US sourced Neulasta (Sample U1 and U5) and EU sourced Neulasta (Sample E1, E4 and E5).

The results of peptide mapping by LC-ESI-MS/MS for determination of sequence coverage is presented in Table B in S1 Appendix and Fig 1. The N-terminal peptide (G1, amino acids 1–20) did not produce a conclusive MS result due to the heterogeneous nature of the PEG moiety attached to the N-terminus. The sequence data of each peptide was confirmed to be identical by MS/MS analysis. For all samples, obtained experimental mass of all peptides was matching with that of the theoretical mass.

The assessment of higher order structural integrity and conformational stability of proteins in comparability studies was performed using multiple biophysical and calorimetric techniques.

**Fig 2:**
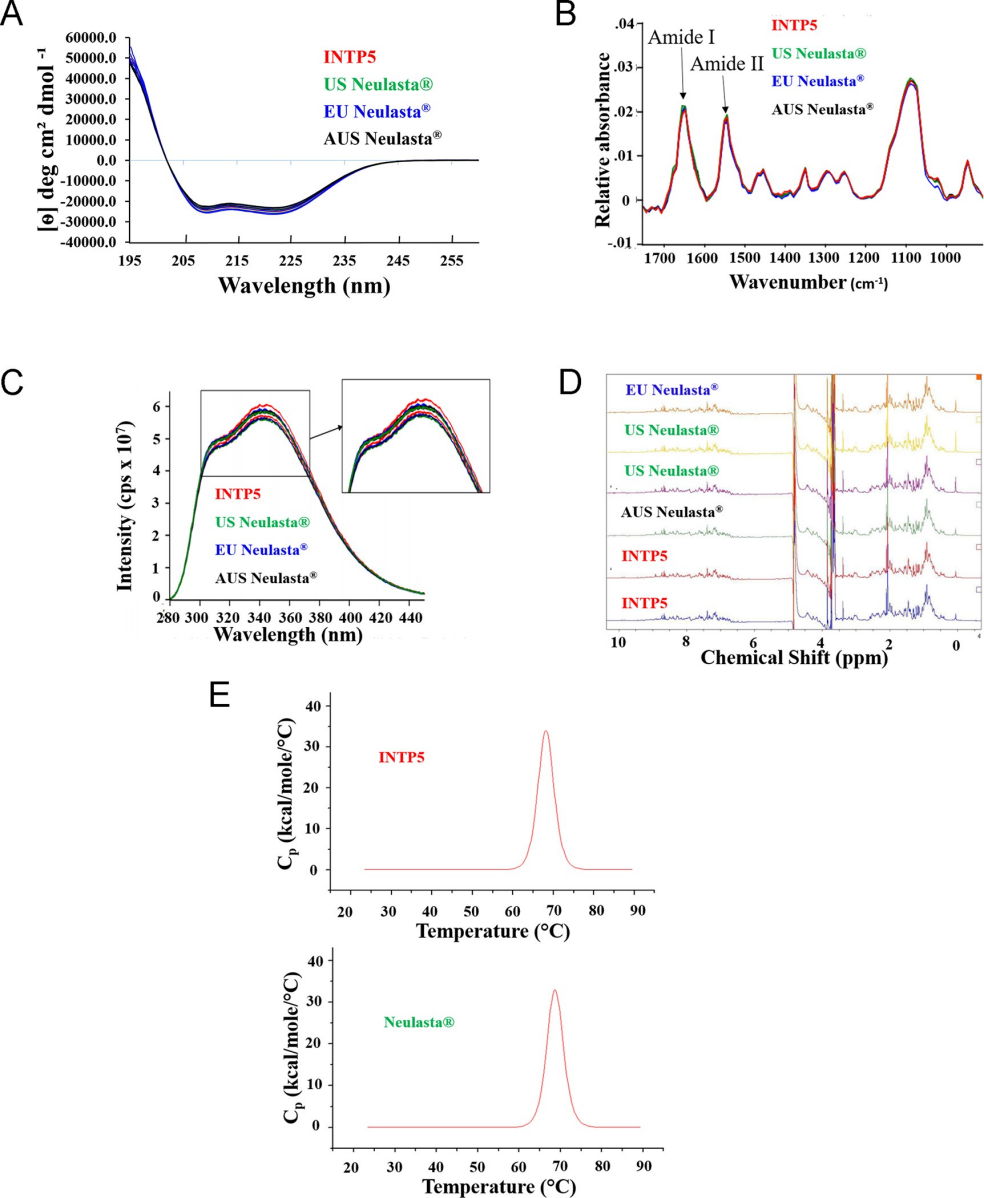
(A) Overlay of far UV CD spectra, (B) Overlay of FTIR spectra, (C) Overlay of fluorescence spectra, (D) overlay of 1D NMR spectra and (E) DSC thermogram of INTP5 and Neulasta.

Far-ultraviolet circular dichroism (Far UV-CD) and fourier transform infrared spectroscopy (FTIR) was used to analyze the overall secondary structure content. Overlays of the far UV CD spectra (mean residue ellipticity vs wavelength) are shown in 2 (A) that show clear spectral overlay for INTP5 with originator products. Two minima, one around 209 nm and another around 221 nm that are characteristic of a significant alpha helical structure were observed for both INTP5 and Neulasta samples (Table C in S1 Appendix). The observed minor variations are due to the day-to-day experimental variations. Multiple batches of the same product are assessed to show that the variation is observed amongst the lots of the same product as much as they are observed between the lots of product from the originator and INTP5. The mean and standard deviation of the observed minima for INTP5 and Neulasta samples are also comparable (Table C in S1 Appendix). This indicates that the secondary structure of INTP5 and Neulasta is similar. The IR spectra of INTP5 and Neulasta is shown in Fig 2(B). A spectral overlay of the FTIR spectra of INTP5 with different Neulasta samples showing similarity of the amide I band ~ around 1654 cm^-1^ and amide II band ~ around 1548 cm^-1^ also indicates similar secondary structure.

Intrinsic fluorescence spectroscopy and NMR, were used to compare the tertiary structural details with respect to the protein’s folded structure. Intrinsic fluorescence spectroscopy was performed at excitation wavelengths of 271 nm and 291 nm. The fluorescence spectra when excited at 271 nm are presented in Fig 2(C), which shows that INTP5 and Neulasta have two fluorescence emission peaks around ~308 nm and ~ 345 nm with similar emission fluorescence intensities. The peak at ~308 nm corresponds to tyrosine (Tyr) fluorescence and the peak at ~ 345 nm to tryptophan (Trp) fluorescence. For excitation at 291 nm the fluorescence intensity at 345 nm (Table D in S1 Appendix) also shows comparable fluorescence intensities. Overall, this indicates that the environment of the tyrosine and tryptophan residues are similar for INTP5 and Neulasta. Hence, INTP5 and Neulasta are highly similar in terms of tertiary structure when analyzed by intrinsic fluorescence spectroscopy.

**Fig 3:**
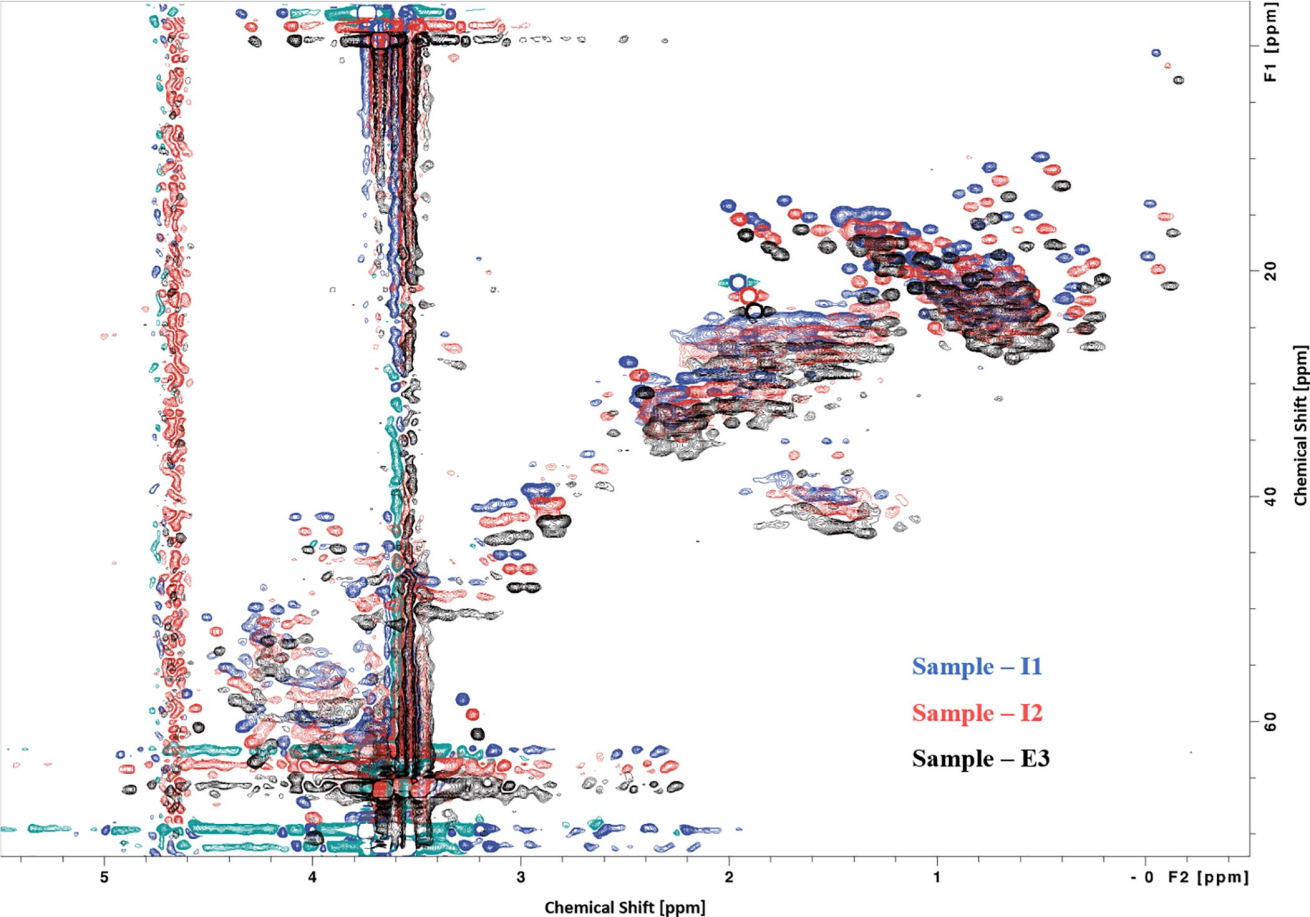
Shifted overlay of ^1^H-^13^C HSQC spectrum for INTP5 (Sample I1, Sample I2) and EU sourced Neulasta (Sample E3).

One-dimensional (1D) NMR provides information about protein structure and is useful in qualitative comparisons whereas two-dimensional (2D) NMR provides greater information about the overall secondary and tertiary structure of a protein. The 1D NMR spectra of INTP5 and Neulasta samples are shown in Fig 2(D). The signal pattern and relative intensities of the complete spectra of all the samples match with each other, indicating the similar nature of all the samples. Natural abundance 2D ^1^H-^13^C HSQC spectra were recorded for INTP5 and Neulasta samples and the aliphatic portion of the spectra exhibit identical chemical shift pattern as shown in the shifted overlay of 2D ^1^H-^13^C HSQC spectra for representative samples (I1, I2 and E3) Fig 3. Chemical shift distribution and weighted sum intensity of the peaks in the aliphatic region (Table E in S1 Appendix) that correspond to the high field methyl groups (^1^H -0.5 ppm to 2 ppm; ^13^C 0.0 ppm to 30 ppm) that belong to methyl groups proximal to aromatic rings within the protein core also show a high degree of correlation (Table F in S1 Appendix), indicating similarity in terms of tertiary structure.

Differential scanning calorimetry (DSC) was used to monitor conformational stability of product. The results of INTP5 and Neulasta showed a thermal transition profile of one major peak centered at ~68–70°C as shown in Fig 2(E). Range observed from calculated transition temperatures (Tm) for all samples are shown in Table G in S1 Appendix. The observed values indicate that all protein samples are monomeric with a single domain structure, and the overall structural stability is similar. A summary of the structural similarity assessment in provided in Table 1.

**Table 1:** List of methods used for assessment of primary structure, higher order structure and functional characteristics of INTP5, EU sourced Neulasta and US sourced Neulasta.

Quality Attribute	Analytical Procedure	Conclusion
Primary structure analysis	N terminal sequence	The 2–20 amino acids are identical for INTP5 and EU and US Neulasta. The first amino acid is not detected due to involvement in pegylation in all 3 products.
	Peptide map analysis- MS/MS analysis	For all peptides, obtained experimental mass is matching with that of the theoretical mass. The chromatograms of INTP5 and EU and US Neulasta are exactly overlapping indicating the identity of the amino acid sequence.
	PEG Linkage analysis by NMR	The identity of the N terminal methionine and its linkage PEG was confirmed by 1D and 2D NMR to be identical in INTP5 and EU and US Neulasta.
Higher order structure analysis	Far UV- Circular Dichroism (CD) Spectroscopy	The CD spectra are overlapping with 2 minima observed at ~ 209–210 nm and ~ 221–222 nm for INTP5 and EU, US and AUS Neulasta
	FTIR	FTIR spectra of INTP5 and EU, US and AUS Neulasta samples are similar for amide I and amide II bands
	1D NMR	Samples display a high degree of similarity in the signals that originate from INTP5 and EU and US Neulasta. The products show highly similar NMR profiles.
	2D NMR	
	Differential Scanning Calorimetry (DSC)	The Tm values of INTP5 are highly similar to that of EU and US Neulasta, with the Tm ranging between 68.2–69.2 ^o^ C for all the products.
	Fluorescence Spectroscopy	INTP5 and EU and US Neulasta show highly overlapping emission spectra.
Functional characteristics analysis	*in vitro* Cell Proliferation Assay (*in vitro* bioassay)	The % relative potencies for various lots tested were in in the range of 93–110% for INTP5; 88–107% for EU sourced Neulasta and 89–109% for US sourced Neulasta.
	Receptor Binding Assay (Flow Cytometry)	The % relative binding for various lots tested were in the range of 93–107% for INTP5; 88–108% for EU sourced Neulasta and 96–107% for US sourced Neulasta.
	Receptor Binding Assay (Surface Plasmon Resonance)	The K_D_ values for various lots tested were in the range of 8.10 E-11 to 2.60 E-10 for INTP5; 1.70E-10 to 3.70E-10 for EU sourced Neulasta and 6.60E-11 to 2.20E-10 for US sourced Neulasta.

### Functional similarity assessment

To establish similarity for functional quality attributes, three methods were employed i.e. receptor binding assay by flow cytometry, receptor binding assay by SPR and *in vitro* cell proliferation assay.

**Fig 4:**
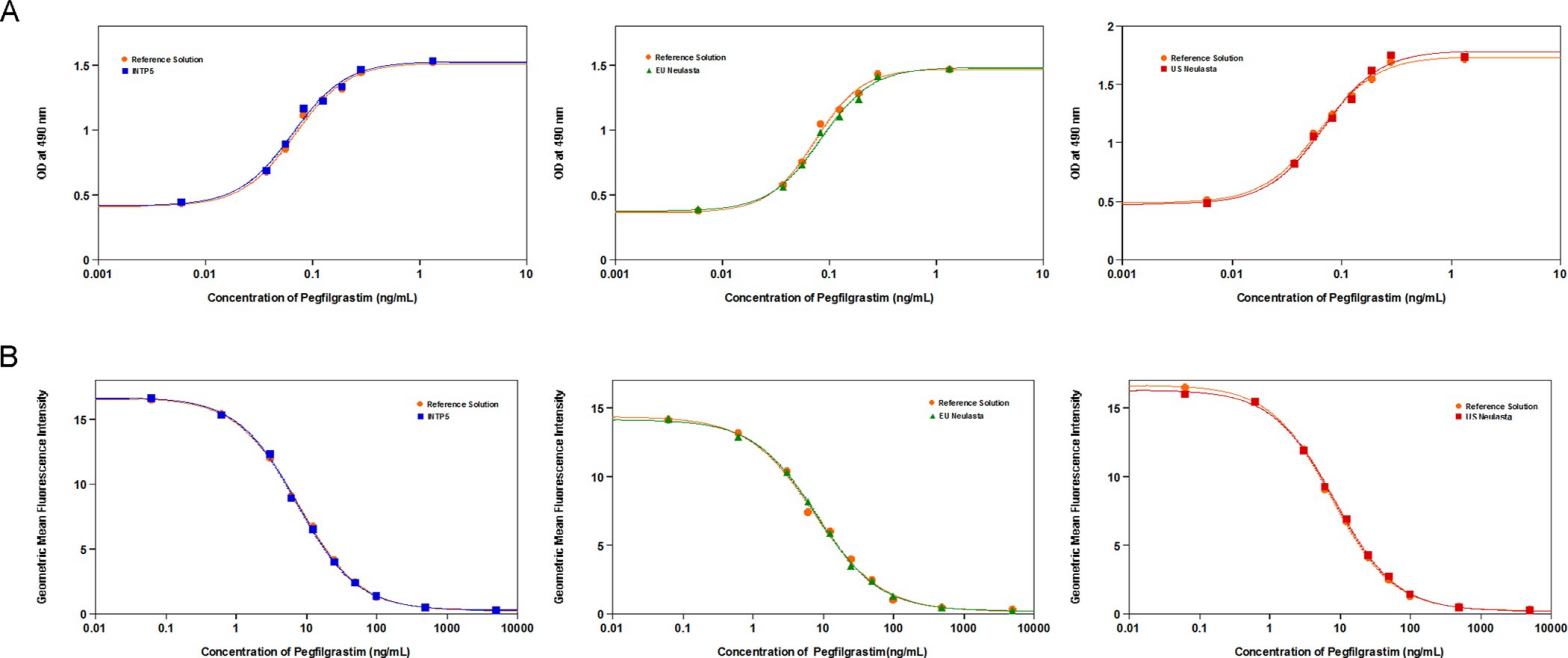
(A) Dose response profile of *in vitro* cell proliferation assay; (B) Dose response profile of receptor binding assay by flow cytometry.

The observed ranges for % relative potency by *in vitro* cell proliferation assay, % relative binding activity by flow cytometry and equilibrium dissociation constant (K_D_) values obtained by SPR are shown in Table 1 and the dose response profile of *in vitro* cell proliferation assay and receptor binding activity by flow cytometry are shown in Fig 4(A) and 4(B). Based on the ranges obtained for INTP5, EU sourced Neulasta and US sourced Neulasta, all the three products are similar in terms of functional attributes.

### Characterization of PEG linkage

Pegylation of peptides and proteins creates significant challenges for detailed structural characterization, such as PEG heterogeneity, site of addition and number of attached pegylated moieties [17]. The site of attachment and linkage of the polyethylene glycol moiety was investigated as part of biosimilarity assessment. A number of lines of evidence have been used to demonstrate that both INTP5 and EU sourced Neulasta and US sourced Neulasta have a single polyethylene glycol molecule attached to the amino terminus of the molecule by a secondary amine linkage. As a confirmation for the site of PEG attachment, N-terminal sequencing (Edman degradation) and peptide map analysis by MS/MS comparative results are provided. The N-terminal sequencing results show that the Met residue at the N-terminus is not detected due to PEG attachment [18] (Table A in S1 Appendix). The peptide-mapping data suggest that the PEG molecule is attached to the N-terminal peptide (amino acids 1–20) (S1 and S2 Figs). The Lys containing peptides are well detected in the peptide map LC-ESI-MS/MS, thus indicating that the site of attachment is not at a Lys residue. The results together indicate that the site of PEG attachment as the amino terminus (Fig 1 and Table B in S1 Appendix). For direct analysis of PEG linkage three lots of INTP5 (Sample I1, I2 and I3), three lots of EU sourced Neulasta (Sample E1, E2 and E3) and three lots of US sourced Neulasta (Sample U1, U2 and U3) were tested by NMR. Analysis of purified pegylated N-terminal peptide was carried out to provide an assessment of the linkage between PEG/Linker and the N-terminal Met residue (to the extent possible). The challenge of applying NMR spectroscopy increases in complexity in relation with the high molecular weight of Neulasta [19].

**Fig 5:**
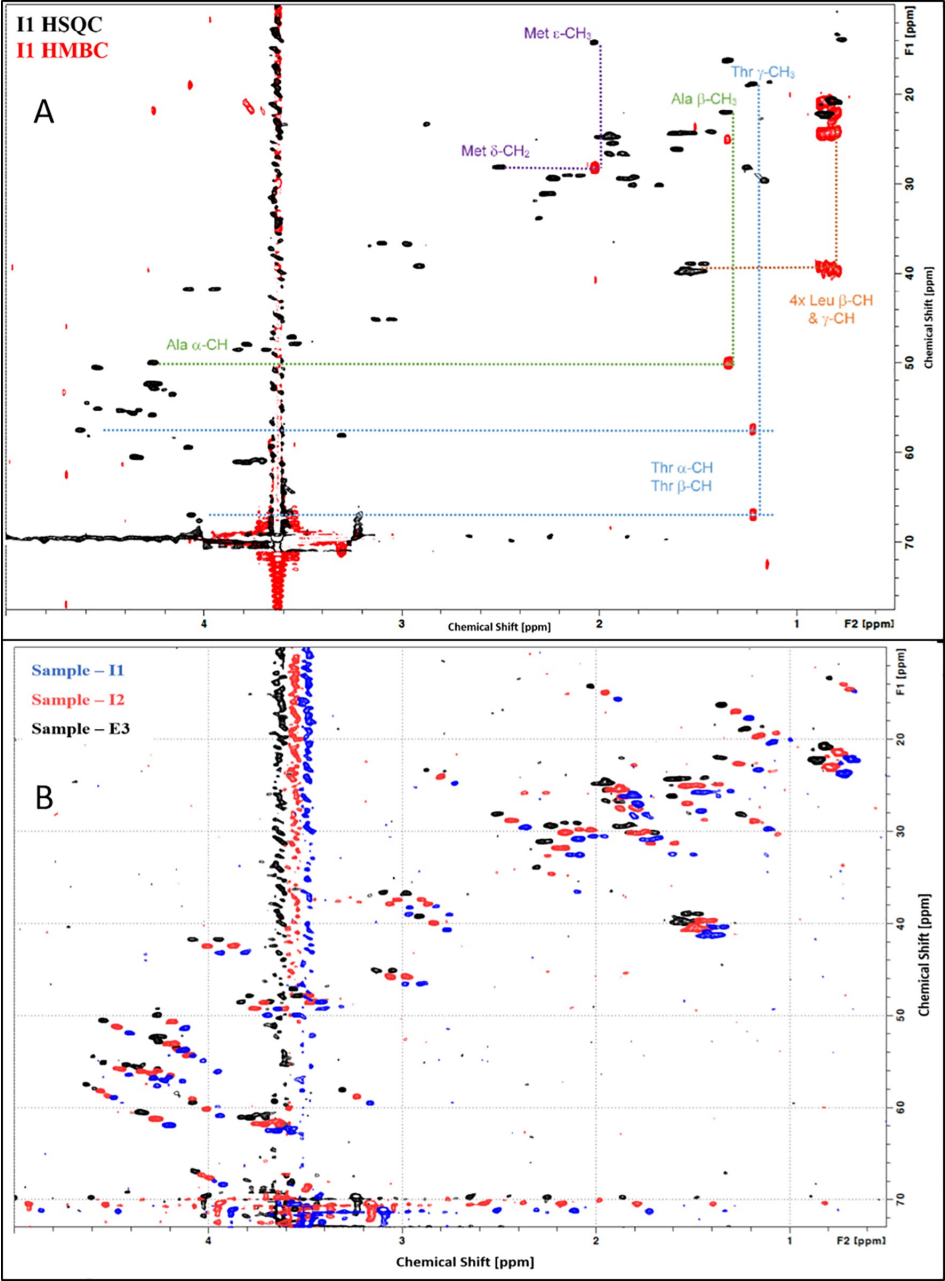
(A) Overlay of ^1^H-^13^C HSQC and HMBC spectra for INTP5 (Sample I1), N-terminal peptide; (B) Shifted overlay of ^1^H-^13^C HSQC spectrum for INTP5 (Sample I1, Sample I2) and EU sourced Neulasta (Sample E3).

A comparison of the 1D ^1^H NMR spectra for all samples is shown with a full aliphatic expansion shown in S3 Fig. The spectra exhibit a features characteristic of a short peptide (amino acid residue 1–17) and the presence of a large PEG moiety at ~3.8 ppm. The aromatic region shows a prominent aromatic side chain spin-system between 7.2–7.4 ppm, which is consistent with the sole phenylalanine in the peptide sequence. To identify further key amino acid assignments (in particular the N-terminal Met) a natural abundance 2D ^1^H-^13^C HSQC spectrum was recorded using sample I1 and compared with an 2D ^1^H-^13^C HMBC (multiple bond correlation) to link pairs of adjacent CH_n_s as shown in Fig 5(A). Natural abundance 2D ^1^H-^13^C HSQC (Heteronuclear Single Quantum Coherence) combined with 2D ^1^H-^13^C HMBC (Heteronuclear multiple bond correlation) spectra provides information of the N-terminal Met side chain. The four leucine residues can be observed through the characteristic Leuβ-γ correlations (orange dotted lines). The single threonine and alanine side chains can also be identified (blue and green dotted lines). The methyl signal for the N-terminal Met residue is well resolved and resonates at a characteristic Met chemical shift (εCH3:^1^H~2.1 ppm and ^13^C~15 ppm) and is readily correlated to the γCH2 on the other side of the sulphur (γCH2:^1^H~2.5 ppm and ^13^C~28 ppm–shown with purple lines). These chemical shifts are consistent with an unmodified Met side chain. If the sulphur were pegylated, the sulphonium ion would induce a significant downfield shift in ^1^H, ^13^C chemical shifts of the adjacent methyl (typically to ^1^H~3 ppm and ^13^C~21 ppm). For sample comparability, natural abundance 2D ^1^H-^13^C HSQC spectra were recorded for each samples and the aliphatic portion of the spectra exhibit identical chemical shifts patterns. Correlation coefficient for peak positions are 1.000 for each pairwise comparison indicating that the samples are indistinguishable from each other in terms of structure, composition and the PEG conjugation site on Met, which must be at the amine group. All samples exhibit identical chemical shifts patterns. To illustrate the similarity a shifted overlay of 2D ^1^H-^13^C HSQC spectra is shown for three example samples (namely NMR samples I1, I2 and E3) in Fig 5(B). It can be confirmed that the PEG conjugation site is on the N-terminal peptide as confirmed by LC-MS data and the direct N-terminal sequencing data confirms that the site is on the first Met residue. In support of the LC MS data, the NMR data for the N-terminal peptide confirms the presence of an intact Lys residue. Further, the NMR data confirms that the N-terminal Met side chain is intact and this concludes that the PEG is attached to the Nitrogen atom of the Met by the secondary amine linkage.

The results of all tests demonstrate that INTP5 is highly similar to EU sourced Neulasta and US sourced Neulasta in all attributes studied. The data presented assures the structural similarity of products starting from primary to higher order structure and finally functionality that assures a functional product conformation.

### Characterization of product related variants

Product related impurities are variants of the desired pegfilgrastim molecule, and include chemically modified species (e.g. oxidized, deamidation and reduced), higher molecular weight variants (e.g. aggregates), variants generated during the pegylation process such as dipegylated variants and positional isomers of monopegylated filgrastim (pegylated at sites other than N-terminal Met). These variants are formed during the processing of the product. Since the production process of biosimilar is different from that of the innovator, it is possible to have different types and levels of such product variants. To assure product safety and efficacy, it is essential to thoroughly characterize the variants in a biosimilar product. Three key analytical methods are used to assess the level of various variants: charge based variants using CEx-HPLC, size based variants using SE-HPLC and hydrophobicity based variants using RP-HPLC. The variants detected by each of the methods were characterized as detailed further. Peaks collected from the purified product, in-process samples, and forced degraded samples were used for characterization of the observed impurities with each method. Other methods such as LC-MS and SDS-PAGE (Sodium Dodecyl Sulfate Polyacrylamide Gel Electrophoresis) were employed for complete identification of the variant and wherever adequate enriched variant was available; the biological activity by *in vitro* bioassay was also assessed.

#### Characterization of impurities separated by CEx-HPLC

**Fig 6:**
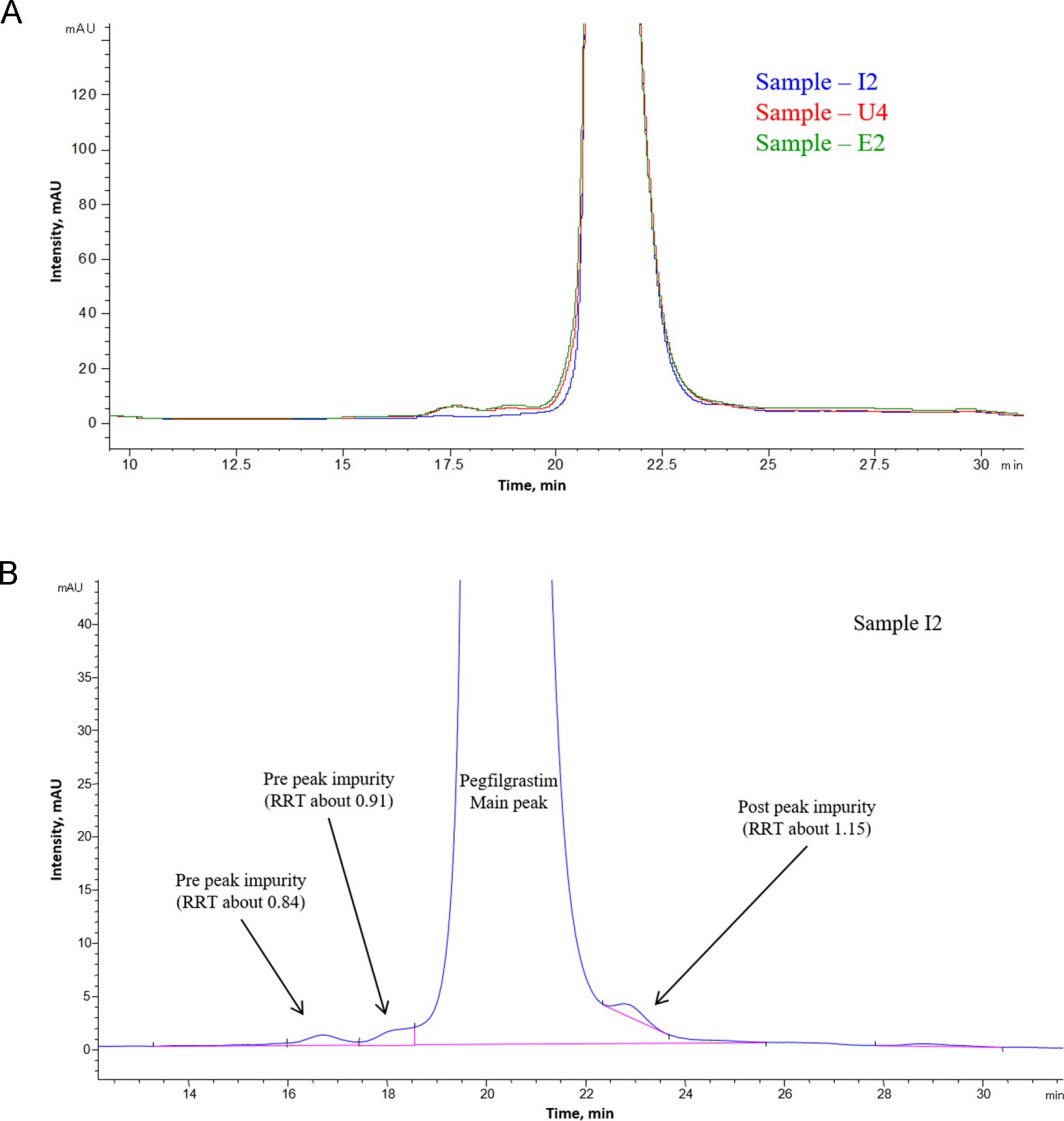
(A) Overlay of CEx chromatographic profile of INTP5, US Neulasta and EU Neulasta (Sample I2, U4 and E2); (B) Representative CEx-HPLC profile of INTP5 (Sample I2).

A representative CEx-HPLC profile, zoomed to show the impurities, is provided in Fig 6(A) (overlay of INTP5 with EU and US Neulasta). The level of the impurities present in the samples is provided in Table 2.

**Table 2:** Quantitative purity analysis of INTP5, US Neulasta and EU Neulasta by CEx-HPLC.

CEx-HPLC (Method LOD is 0.05% and LOQ is 0.2% of 100 μg)				
Sample Name		INTP5 (n = 16)	US Neulasta (n = 18)	EU Neulasta (n = 11)
% Main peak	Average	99.7	98.7	98.9
	Range (Min-Max)	99.2–100.0	98.6–99.2	98.6–99.1
% total impurities	Average	0.3	1.3	1.1
	Range (Min-Max)	<LOQ-0.8	0.8–1.5	0.9–1.4

Note: ‘n’ indicates number of product batches evaluated, with each result corresponding to one batch.

**Fig 7:**
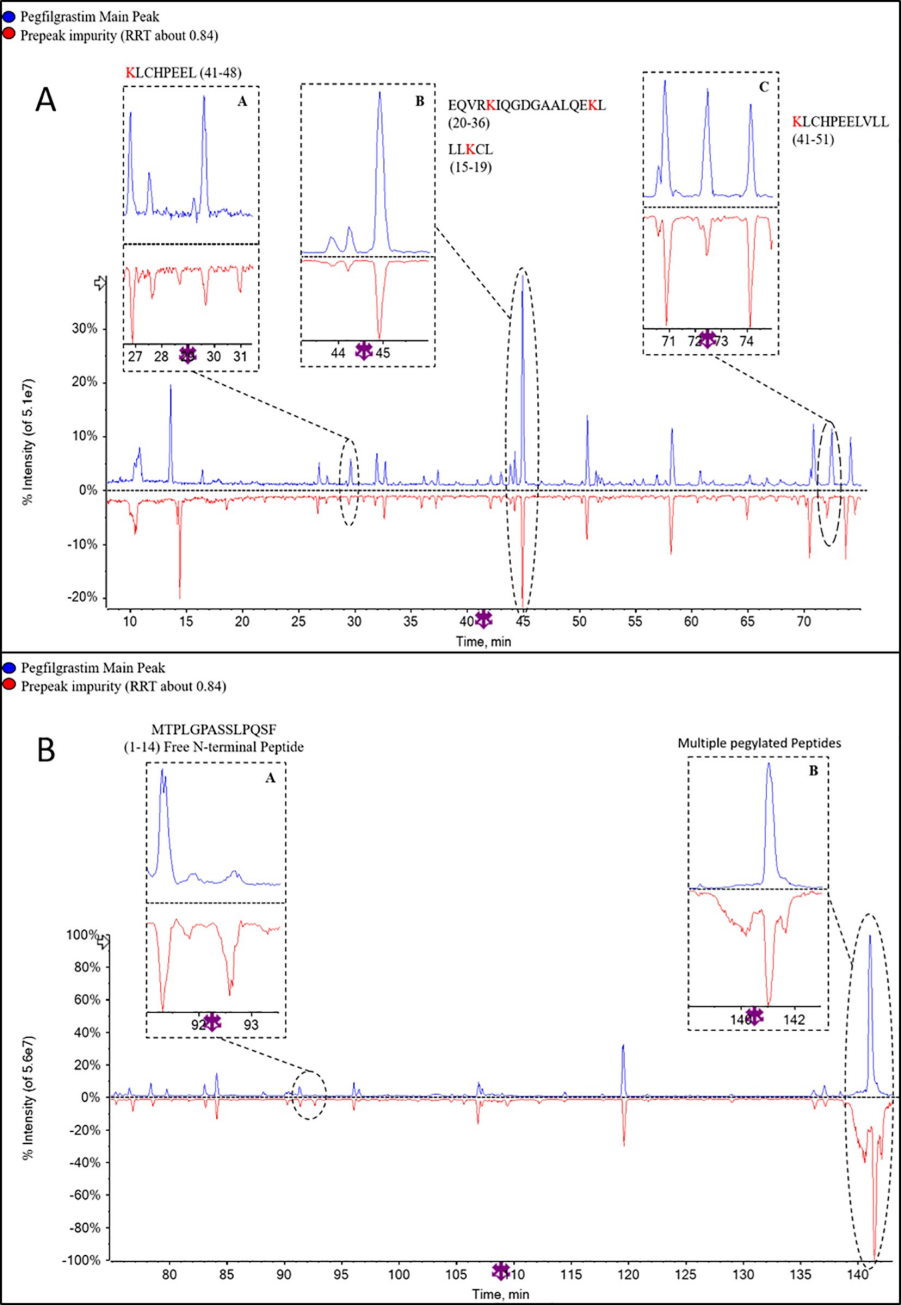
(A) TIC overlay of pegfilgrastim main peak and Pre-peak impurity (RRT 0.84) in peptide mapping (10–75 min) (B) TIC overlay of pegfilgrastim main peak and Pre-peak impurity (RRT 0.84) in peptide mapping (75–145 min).

The purified, buffer exchanged and concentrated CEx-HPLC impurities from Sample I2 (INTP5) (Fig 6(B) were preliminarily analyzed by non-reduced SDS-PAGE (S4 Fig). The result indicates that the major band in all the samples corresponds to the position is similar to the principal band of 100% reference solution of pegfilgrastim suggesting monopegylated nature of the collected impurities. Further confirmation of impurity was done by peptide mapping (chymotrypsin digestion) using LC-MS analysis. Comparison of the peptide mapping TIC (Total Ion Chromatogram) profile of the impurity RRT (Relative Retention Time) about 0.84, impurity RRT about 0.91 and impurity RRT about 1.15 samples with pegfilgrastim main peak sample (control sample) was performed to identify any differences (Fig 7(A) and 7(B), S5–S8 Figs). Major differences observed are encircled and zoomed images are provided. The significant differences shows that free N-terminal peptide peak is observed at Rt ~ 92 min in impurity RRT about 0.84 (Fig 7(B)) and impurity RRT about 1.15 (S8 Fig). This free N-terminal peptide peak is present in the negligible amount in the main peak sample indicating that filgrastim is pegylated at a site different than the N-terminus in the impurity sample. There are multiple peaks (appearing as humps) eluting near 140 min in the impurity sample RRT about 0. 84 (Fig 7(B)) and RRT about 1.15 (S8 Fig), which are not present in main peak sample. Mass spectra of these peaks are similar to the mass spectrum obtained for the pegylated peptide [20, 21]. This indicates that there are other pegylated species apart from N-terminally pegylated pegfilgrastim. TIC intensities of Lys41 containing peptides (majorly Rt ~30 min and ~72 min) are lesser in the impurity sample RRT about 0.84 than the pegfilgrastim main peak sample (Fig 7(A)). This indicates that the CEx variant at RRT about 0.84 is likely to be a pegylated variant with PEG attached to Lys41. For impurity sample RRT about 1.15, a drop (~20%) is observed in the TIC intensity of the peptide containing Lys35 and 24 containing peptides peaks (~45 min) with respect to the pegfilgrastim main peak sample (S7 Fig), which indicates further possibility of pegylation of the Lys (Lys35/ Lys24) in the impurity sample RRT about 1.15.

Based on the above results, the variants eluting at RRT about 0.84 and RRT about 1.15 are mainly a mixture of positional isomers of monopegylated filgrastim (filgrastim pegylated at a different site than the N-terminus). For the impurity sample RRT about 0.91, the TIC comparison shows deamidation at Gln174 (Rt ~13 min) which is not present in the pegfilgrastim main peak sample (S5 Fig). Approximately 48.6% of deamidation was observed at Gln174 while minor deamidation at different glutamine position was also observed (~9.2%). Minor amount of dipegylated pegfilgrastim variants were observed in RRT about 0.84 and RRT about 0.91 impurity peaks (S6 Fig). In all the samples, minor amount of oxidation is observed possibly due to sample processing [7]. All the three variants in CEx-HPLC show a low level of multi-pegylation as seen by the LC profiles showing multiple peaks at the position where the N-terminally pegylated peptide elutes. Moreover, the SDS-PAGE data (S4 Fig) indicates the presence of high molecular weight species which could be dimers and aggregates or dipegylated versions.

The above data can also be supported by the fact that the pegylation process involves attachment of a polyethylene glycol molecule, activated with an aldehyde moiety, to the N-terminal amino group of filgrastim by a secondary amine linkage in order to produce pegylated filgrastim. There are other primary amino groups present in the filgrastim molecule on the side chains of Lys residues at amino acid position number 17, 24, 35 and 41 which could potentially serve as unwanted sites of attachment of polyethylene glycol resulting in positional isomer, dipegylated and/or multi-pegylated species.

**Fig 8:**
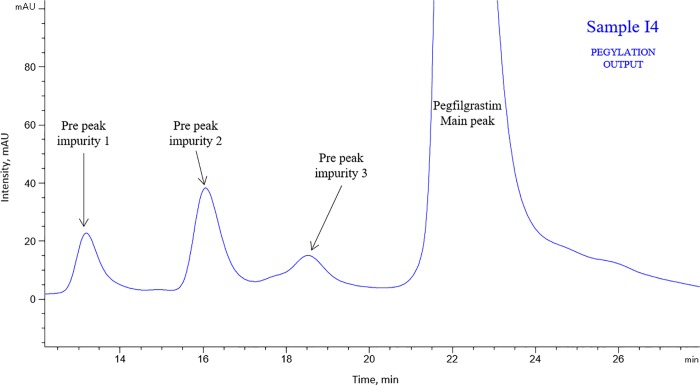
Representative CEx-HPLC profile of POP Sample I4 showing Pre-peak impurity 1 (RRT 0.60), Pre-peak impurity 2 (RRT 0.73) and Pre-peak impurity 3 (RRT 0.84).

The unpurified pegylation output (POP) sample consists of all these impurities that are subsequently removed by purification steps. Since this sample is an enriched source of the impurities, it was analyzed by CEx-HPLC to identify impurity peaks, and all major impurity peaks (Fig 8) separated in CEx-HPLC profile of pegylation output were collected, concentrated and purified. Purity of collected impurities was found to be > 95% by CEx HPLC.

**Fig 9:**
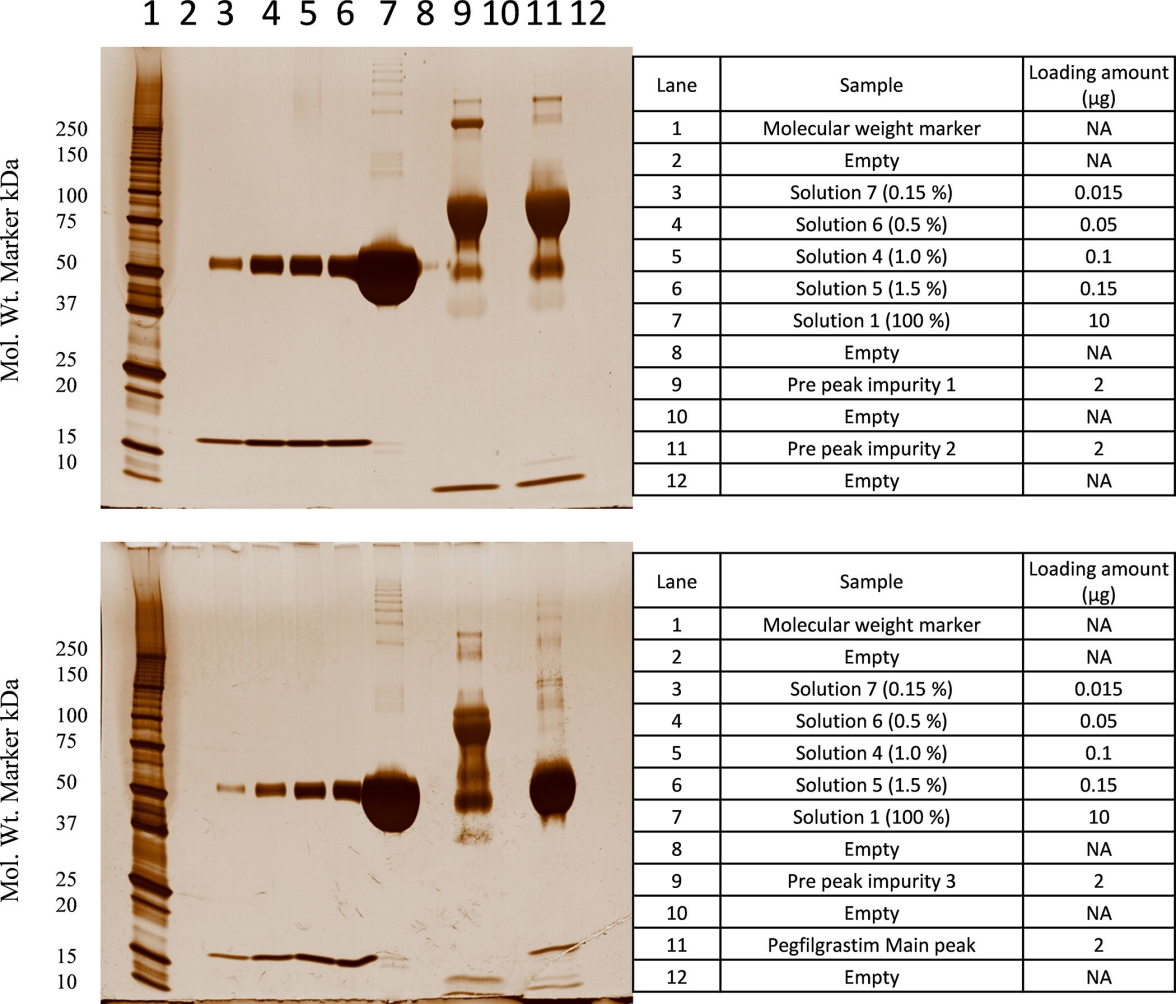
SDS-PAGE analysis of Sample I4-CEx impurity peaks (pegylation mixture output). (Solution 1, Solution 4, Solution 5, Solution 6 and Solution 7 are the internal Reference Standard solutions).

The purified impurity peaks were preliminarily analyzed by non-reduced SDS-PAGE silver stained method. As evident from Fig 9, there is no band observed near the 15 kDa marker band suggesting absence of any free filgrastim in the impurity samples. Higher molecular weight impurities were observed as a major band in pre-peak impurity 1 (RRT 0.60), 2 (RRT 0.73) and 3 (RRT 0.84) samples between the 75–100 kDa marker bands indicating the possibility of dipegylated species. Pre-peak 3 impurity (RRT 0.84) also had a major band at the size corresponding to the principal band indicating a likelihood of monopegylated positional isomers in this impurity. Additionally, pre-peak impurity 3 in POP sample matches with the position of the Pre-peak impurity in CEx-HPLC of INTP5 at RRT 0.84 peak. For further confirmation of the impurity, samples digested using chymotrypsin and Glu-C were analyzed by LC-MS and MS/MS based peptide mapping.

**Fig 10:**
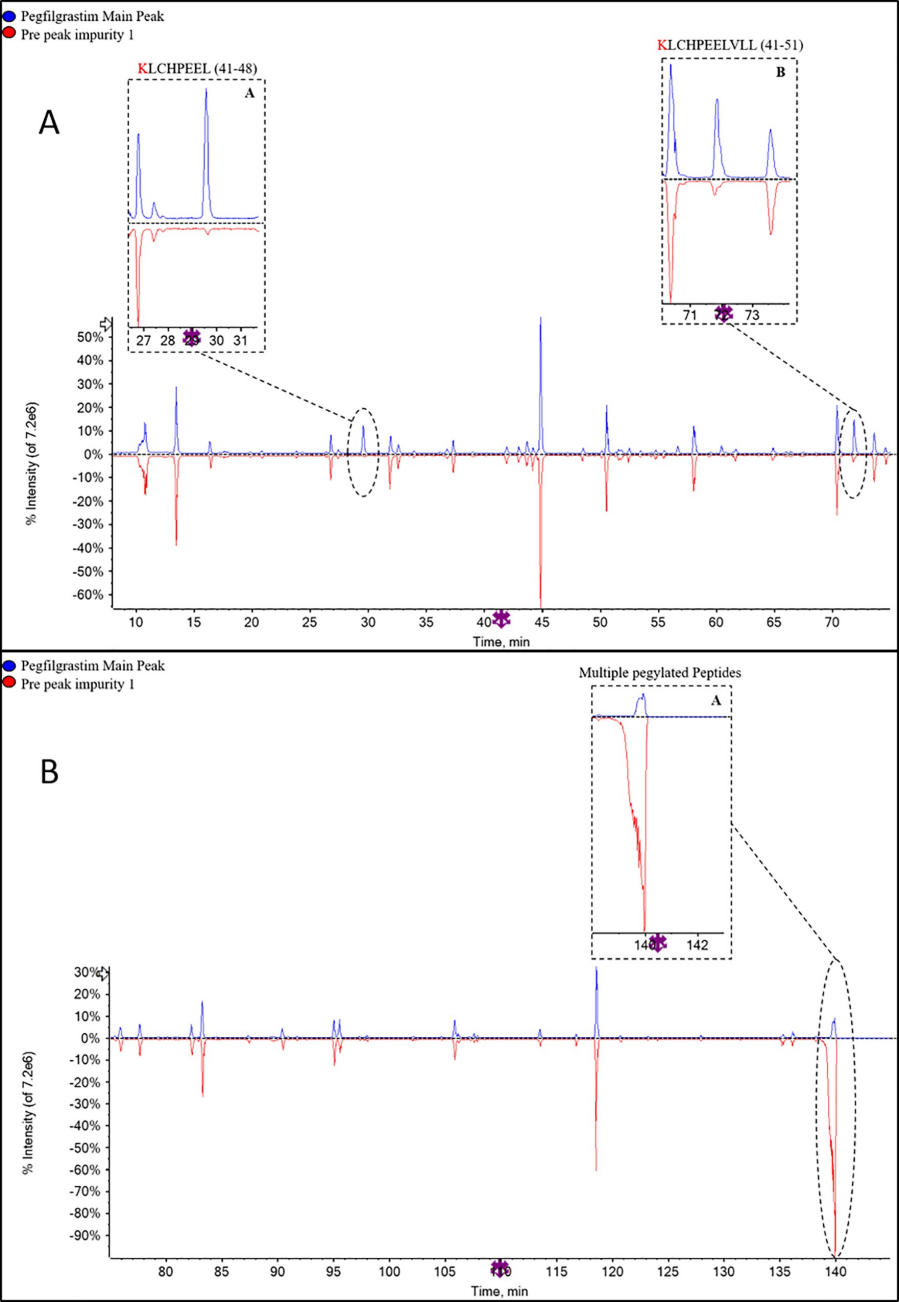
(A) TIC overlay of pegfilgrastim main peak and Pre-peak impurity 1 (RRT 0.60) in peptide mapping (10–75 min) (B) TIC overlay of pegfilgrastim main peak and Pre-peak impurity 1 (RRT- 0.60), TIC of peptide mapping (75–145 min).

Comparison of peptide mapping (TIC) profile of pre-peak impurity 1, pre-peak impurity 2, pre-peak impurity 3 and the pegfilgrastim main peak was performed. Major differences observed are encircled and zoomed image is provided in PIP (Picture In Picture) (Fig 10(A) and 10(B), S9–S13 Figs). In pre-peak impurity 1 (RRT– 0.60), peaks corresponding to Lys41 containing peptides (Rt ~30 min and ~72 min) are missing in the impurity sample with respect to the main peak digest (Fig 10(A) and 10(B)) which indicates the possibility of pegylation of the Lys41 present in these peptides. In Pre-peak impurity 2 (RRT 0.73), peaks corresponding to Lys35 or Lys24 containing peptides (Rt ~45 min) are missing in the impurity sample with respect to the main peak digest (S9 Fig) which indicates the possibility of pegylation of Lys35 or Lys24 in these peptides. The confirmation of the pegylation site for this sample was obtained by endoproteinase Glu-C peptide mapping, in which the peptide containing Lys24 at Rt ~14 is present while the peptide containing Lys35 at Rt ~17 is missing in the chromatogram (S11 Fig) (There are two peptides eluting at Rt ~17, one peptide corresponding to the amino acid 35–47 is missing while another peptide is present as seen in S11 Fig; confirmed based on MS data). Also, the absence of N-terminal peptide in both the impurities at ~92 min indicates complete pegylation at N-terminal (Met1). There are peaks (appearing as humps) eluting near 140 min in the pre-peak impurity 1 and 2 samples that were negligible in the pegfilgrastim main peak sample (Fig 10(B) and S10 Fig). Mass spectra of these peaks are similar to the mass spectrum obtained for the pegylated peptide (high ionization in TIC) [20, 21]. This indicates that there are other pegylated species apart from N-terminally pegylated filgrastim. Based on SDS-PAGE and LC-MS data, it can be inferred that the impurity peak 1 is dipegylated pegfilgrastim variants at position Met1 and Lys41 whereas pre-peak impurity 2 is dipegylated pegfilgrastim variants at Met1 and Lys35.

In Pre peak Impurity 3 (RRT– 0.84), the free N-terminal peptide peak is observed at ~ 92 min in impurity 3 (S13 Fig). This peak is absent in the pegfilgrastim main peak sample. The absence of free filgrastim in main peak and presence of free N-terminal peptide peak indicates this impurity has filgrastim pegylated at a site different than the N-terminus. There are multiple peaks (appearing as humps) eluting near 140 min in the pre-peak impurity 3 sample that are not present in the pegfilgrastim main peak digest (S13 Fig). Mass spectra of these peaks are similar to the mass spectrum obtained for the pegylated peptide [20, 21]. This further indicates that there are other pegylated species apart from N-terminally pegylated filgrastim. Moreover, a drop is observed in the intensity of Lys35 and Lys24 containing peptide peak (~45 min) and Lys17 containing peptide peak (~44 min) in the impurity sample with respect to the pegfilgrastim main peak digest (S12 Fig) which further indicates the possibility of pegylation of Lys at these positions.

Based on SDS-PAGE and above observations it can be inferred that the impurity peak 3 is mainly the dipegylated form with pegylation at N-terminal Met and Lys17 or Lys24 or Lys35 and minor positional isomers with pegylation except N–terminal Met. Since the pre-peak impurity 2 was identified to be pegylated at Lys35 in addition to the Met1, and is eluting distinctly in the CEx HPLC, it can be concluded that pre-peak impurity 3 would be pegylated at Lys17 or Lys24 in addition to Met1.

Furthermore, the *in vitro* cell proliferation assay data shows that pre-peak impurities 1, 2 and 3 do not have any biological activity whereas the main pegfilgrastim peak has activity similar to the reference standard (S14 Fig, Table 3).

**Table 3:** Summary of characterization of Pre-peak Impurities 1, 2, 3 and pegfilgrastim main peak from the pegylation reaction output.

Sr. No.	Sample Name	Molecular characteristics	% Relative potency
1	Pre Peak impurity 1 (RRT 0.60)	Dipegylated pegfilgrastim variant (Met1 and Lys41)	No dose response observed
2	Pre Peak impurity 2 (RRT 0.73)	Dipegylated pegfilgrastim variant (Met1 and Lys35)	
3	Pre Peak impurity 3 (RRT 0.84)	Dipegylated pegfilgrastim variants and positional isomers of monopegylated filgrastim	
4	Pegfilgrastim main peak	Native molecule	90

The identification of the impurities from the pegylation output indicates that dipegylated variants with pegylation at Lys residue in addition to the Met1 are present and these are largely removed during the purification process. Only very low level of these impurities is observed in the final purified drug product (Table 2). The data of the pegylation output further confirms that the observed impurities in CEx HPLC originate from the pegylation process.

#### Characterization of variants observed in RP-HPLC

**Fig 11:**
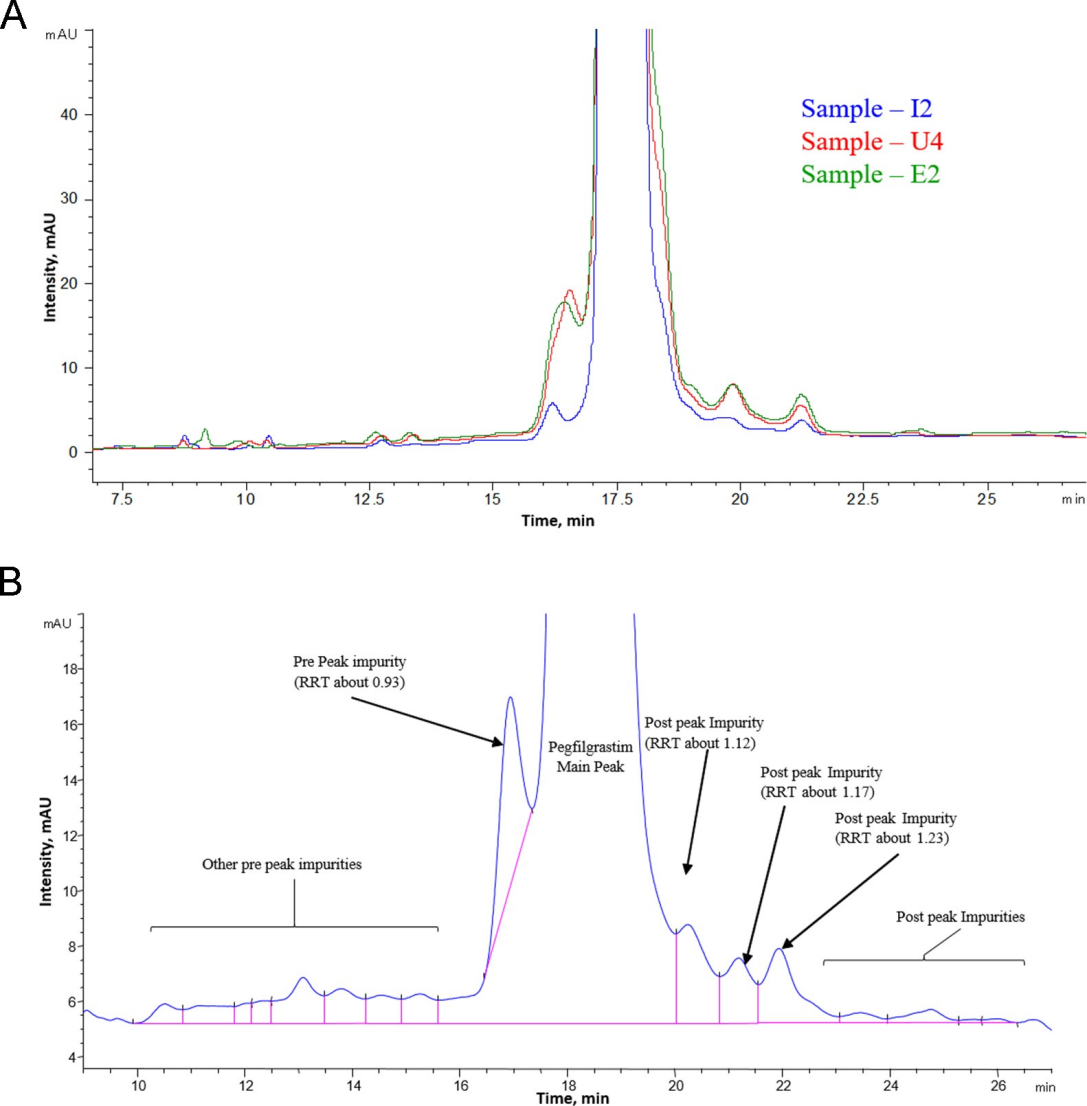
(A) Overlay of RP chromatographic profile of INTP5, US Neulasta and EU Neulasta (Sample I2, U4 and E2) (B) Representative RP-HPLC profile of INTP5 zoomed at the baseline to reveal the maximum possible impurities.

A representative RP-HPLC profile obtained for biosimilar INTP5 is provided in Fig 11(A). Reverse phase chromatography of pegfilgrastim is known to separate oxidized impurities, reduced forms, deamidated impurities and free filgrastim impurities [7]. The impurity peaks at RRT 0.93, 1.12, 1.17, and 1.23 are the major impurities observed in Sample I2 (Fig 11(B)). However, the total level of impurities (average) in Sample I2 is ~ 1.0% (Table 4), which poses difficulties in collecting the required amount of impurity for characterization using a control (non-treated) sample. Hence, characterization of impurities was performed by impurity collection from stressed samples.

**Table 4:** Quantitative purity analysis of INTP5, US Neulasta and EU Neulasta by RP-HPLC.

RP-HPLC (Method LOD is 0.01% and LOQ is 0.1% of 30 μg)				
Sample Name		INTP5 (n = 16)	US Neulasta (n = 18)	EU Neulasta (n = 11)
% Main peak	Average	98.9	98	98.1
	Range (Min-Max)	98.4–99.2	97.2–98.4	97.8–98.5
% total impurities	Average	1.0	2.1	1.9
	Range (Min-Max)	0.1–1.6	1.6–2.8	1.5–2.2

Since it is known that oxidized and deamidated variants are typically resolved in RP-HPLC, oxidized and deamidated variants were generated independently by stressing the sample to identify these variants. The purified variants from the stressed samples were compared to the INTP5 to identify the impurities.

**Fig 12:**
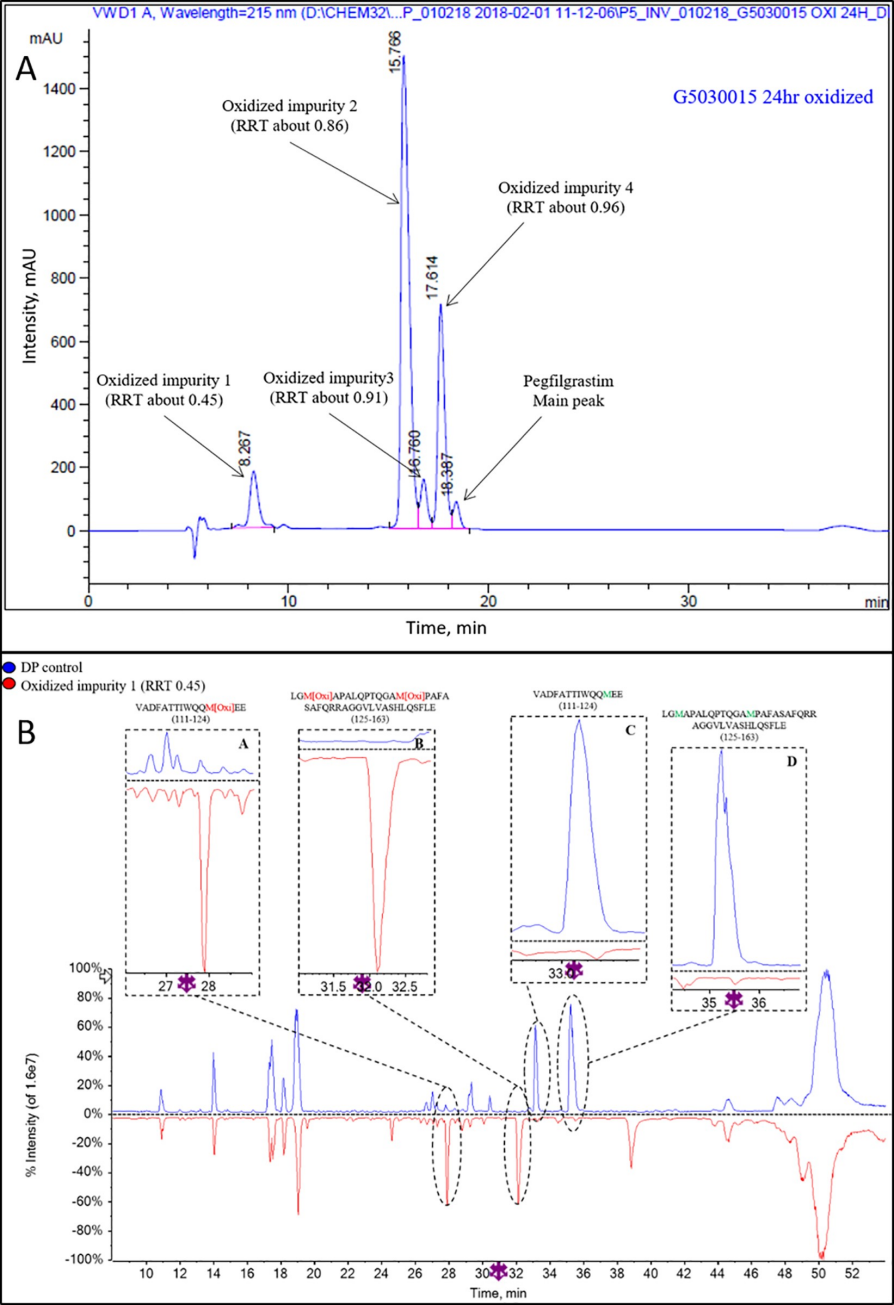
(A) Representative RP-HPLC profile of oxidized (24 hours) INTP5 Sample I2 (B) Overlay of control sample and oxidized impurity 1 (RRT About 0.45) TIC of peptide mapping (10–54 min).

**Oxidized impurities.** Impurities peaks observed in forced oxidized sample are at RRT 0.45, 0.86, 0.91 and 0.96 (Fig 12(A)). The purity of collected, buffer exchanged and purified impurities was found to be > 80% by RP HPLC. Collected impurity peaks were further characterized by LC/MS/MS based peptide mapping using endoproteinase Glu-C.

A comparison of the peptide mapping (TIC) profiles of oxidized impurity 1 (RRT about 0.45) and INTP5 control (sample I2) shows that the native Met containing peptides appearing at Rt ~33 min and ~35 min (Fig 12(B), PIP C and D) in the INTP5 Control sample, having molecular weight of 1667.8 Da (Amino Acid or AA 111–124) and 4025.1 Da (AA 125–163) respectively, are completely missing in the oxidized impurity 1 (RRT about 0.45) sample. Instead there are two new peaks at Rt ~28 min and ~32 min, with difference of ~+16 Da having peptide mass of 1683.8 Da (AA 111–124) and ~+32 Da difference having peptide mass of 4057.1 Da (AA 125–163), are observed in the oxidized impurity 1 (RRT about 0.45) sample. Hence, the impurity peak at RRT 0.45 is a tri-oxidized pegfilgrastim impurity with oxidation at Met122, Met127 and Met138 (Table H in S1 Appendix).

For oxidized impurity 2 (RRT about 0.86), MS and MS/MS data (S15 Fig) shows that the native form of Met containing peptides appearing at Rt ~35 min (S15 Fig, PIP B) in the control sample, having a molecular weight of 4025.1 Da (AA 125–163), is completely missing in the oxidized impurity 2 (RRT about 0.86) sample. Instead there is one new peak at ~32 min with a difference of ~+32 Da difference having peptide mass of 4057.1 Da (AA 125–163) is appearing in the oxidized impurity 2 (RRT about 0.86) sample. Hence, the impurity peak is a di-oxidized pegfilgrastim impurity with oxidation at Met127 and Met138.

For oxidized impurity 3 (RRT about 0.91), MS and MS/MS data (S16 Fig) shows that the native form of Met (Met127 and Met138) containing peptides appearing at ~35 min (S16 Fig, PIP C) in the control sample, having molecular weight 4025.1 Da (AA 125–163), is completely missing in the oxidized impurity 3 (RRT about 0.91) sample. Instead there is one major peak at ~34 min with difference of ~+16 Da having peptide mass of 4041.1 Da and one minor peak at ~32 min with difference of +32 Da having peptide mass of 4057.1 Da (AA 125–163), is appearing in the oxidized impurity 3 (RRT about 0.91) sample. Hence, this impurity peak is a majorly identified as mono-oxidized pegfilgrastim impurity at Met127. Minor impurity is identified as Met127 and Met138 di-oxidation.

For oxidized impurity 4 (RRT about 0.96), MS and MS/MS data (S17 Fig) shows that the native form of Met (Met 127 and Met138) containing peptide appearing at ~35 min (S17 Fig, PIP B) in the control sample, having molecular weight 4025.1 Da (AA 125–163), is completely missing in the oxidized impurity 4 (RRT about 0.96) sample. Instead there is one major peak at ~32.5 min with a difference of ~+16 Da having peptide mass of 4041.1 Da (AA 125–163), is appearing in the oxidized impurity 4 (RRT about 0.96) sample. Hence, this impurity is a mono-oxidized pegfilgrastim impurity at Met138.

As evident Table 5, the oxidized impurity 1 and 2 did not show any activity in the *in vitro* cell proliferation assay while oxidized impurity 4 has similar potency to that of reference standard. The 24 hour oxidation sample also showed reduced potency. Oxidized impurity 3 was not assessed for biological activity because of less sample availability. The data indicate that oxidation at Met138 does not impact the biological activity, whereas the oxidation at Met122 and Met127 inhibits activity.

**Table 5:** Characterization of oxidized species (Under forced oxidation stress) RRTs are based on RP-HPLC retention times relative to the main peak corresponding to the Native protein.

Sr. No.	Sample Name	Molecular characteristic	% Relative potency
1	Control sample	Native protein with 3 non oxidised Met residues	99
2	Oxidized (24 hour oxidized)	Mixture of oxidised variants	57
3	Oxidized impurity 1	Variant at RRT 0.45 with oxidation at Met122, 127, 138	No dose response observed
4	Oxidized impurity 2	Variant at RRT 0.86 with oxidation at Met127 and 138	No dose response observed
5	Oxidized impurity 3	Variant at RRT 0.91 with oxidation at Met127	Limited amount, not assessed in bioassay
6	Oxidized impurity 4	Variant at RRT 0.96 with oxidation at Met138	99

**Fig 13:**
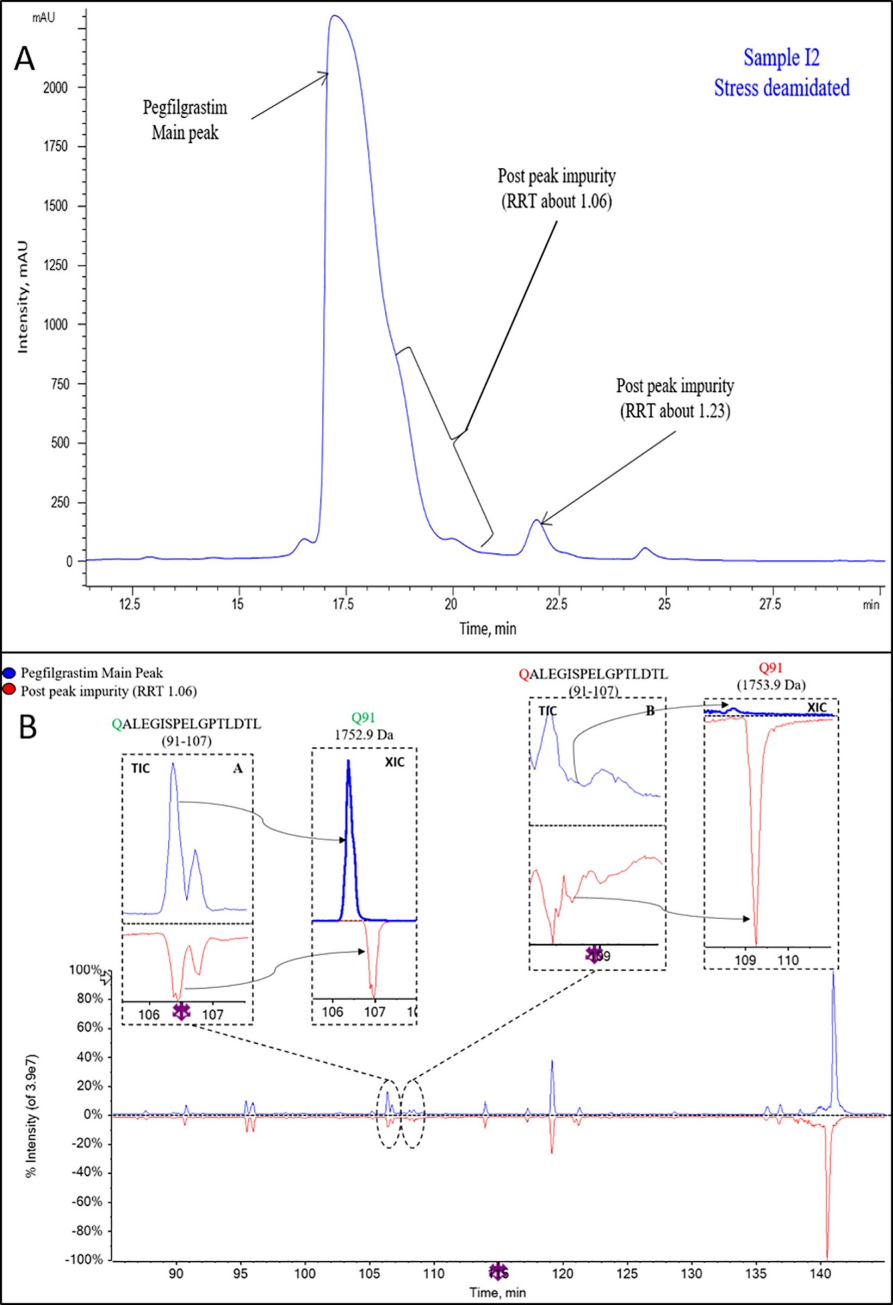
(A) Representative RP-HPLC profile of stressed deamidated INTP5 (Sample I2) for peak collection. (High loading- 500 μg) (B) Overlay of pegfilgrastim main peak and Post-peak Impurity (RRT about 1.06), TIC of peptide mapping (85–145 min) for peptide containing Q91.

**Deamidated impurities.** The stress deamidated INTP5 sample was analyzed using RP-HPLC to identify peaks (Fig 13(A)). Impurity peaks observed in forced deamidated pegylated filgrastim at RRT 1.06 and 1.23 were collected, buffer exchanged and purified. Purity of the collected peaks was checked by RP-HPLC and was found to be >80%. Collected impurity peaks were characterized by LC/MS/MS based peptide mapping using the endoproteinase chymotrypsin. The XIC (Extracted ion chromatogram data) of the Post-peak impurity (RRT about 1.06) (Fig 13(B)) indicates significant change in the deamidation percentage from that of pegfilgrastim main peak. It shows the presence of multiple of mono-deamidated species (bold type font in Table I in S1 Appendix) mainly at different positions of Gln residues such as Q21 or Q26 or Q33, Q87, Q91, Q120 or Q121, Q132 or Q135 (S18 and S19 Figs). It seems that at some Gln positions such as Q12, Q68, Q71 and Q174 deamidation artifacts were generated because of sample processing, as they are also present in similar amounts in the pegfilgrastim main peak (italic type font in Table I in S1 Appendix). Similarly for Post-peak impurity (RRT about 1.23) the XIC data shows the presence of deamidation mainly at the Q108 position (Table I in S1 Appendix).

The biological activity (*in vitro* bioassay) results shows that the Post-peak impurity (RRT about 1.06) has activity of about 77% and is considered as functionally active (taking into consideration the variability associated with cell based assays), whereas the deamidation at Q108 showing an activity of 27% is considered to be inactive as shown in Table 6, and S21 Fig. The Post-peak impurity (RRT about 1.12) was not assessed for biological activity because of less sample availability.

**Table 6:** Summary of characterization of peaks observed by RP-HPLC.

Sr. No.	Impurity by RP HPLC	Molecular characteristics	% Relative potency
1	Pre-peak impurity (RRT about 0.93)	Mono-oxidation at M127 position and dipegylated pegfilgrastim impurities	Inactive (both M127 oxidation and dipegylation render the molecule inactive)*
2	Post-peak impurity (RRT about 1.06)	Mono-deamidation of pegfilgrastim	77
3	Post-peak impurity (RRT about 1.12)	Free filgrastim and reduced pegfilgrastim	Limited amount, not assessed in bioassay
4	Post-peak impurity (RRT about 1.23)	Deamidation at Q108 position	27

*M127 oxidized molecules and dipegylated molecules have been assessed independently as shown in Table 3 and Table 5 and the outcome is derived here from that data, based on the molecular characteristic.

**Identification of impurities in the RP-HPLC profile of the purified drug product.** Based on the studies discussed above, obtained impurities either through the in-process samples or through the forced degradation studies, were individually spiked (at 2% level) into the purified INTP5 to ascertain the nature of impurities in the INTP5 (Sample I2 –S22 Fig). The spiked impurities co-eluted with the observed impurities in the purified product. Therefore, the identity of the impurities in the purified product is established by extrapolation from the known identification of the spiked impurities. The summary of identification of all impurities in RP-HPLC is provided in Table 6.

#### Characterization of variants in SEC-HPLC

**Fig 14:**
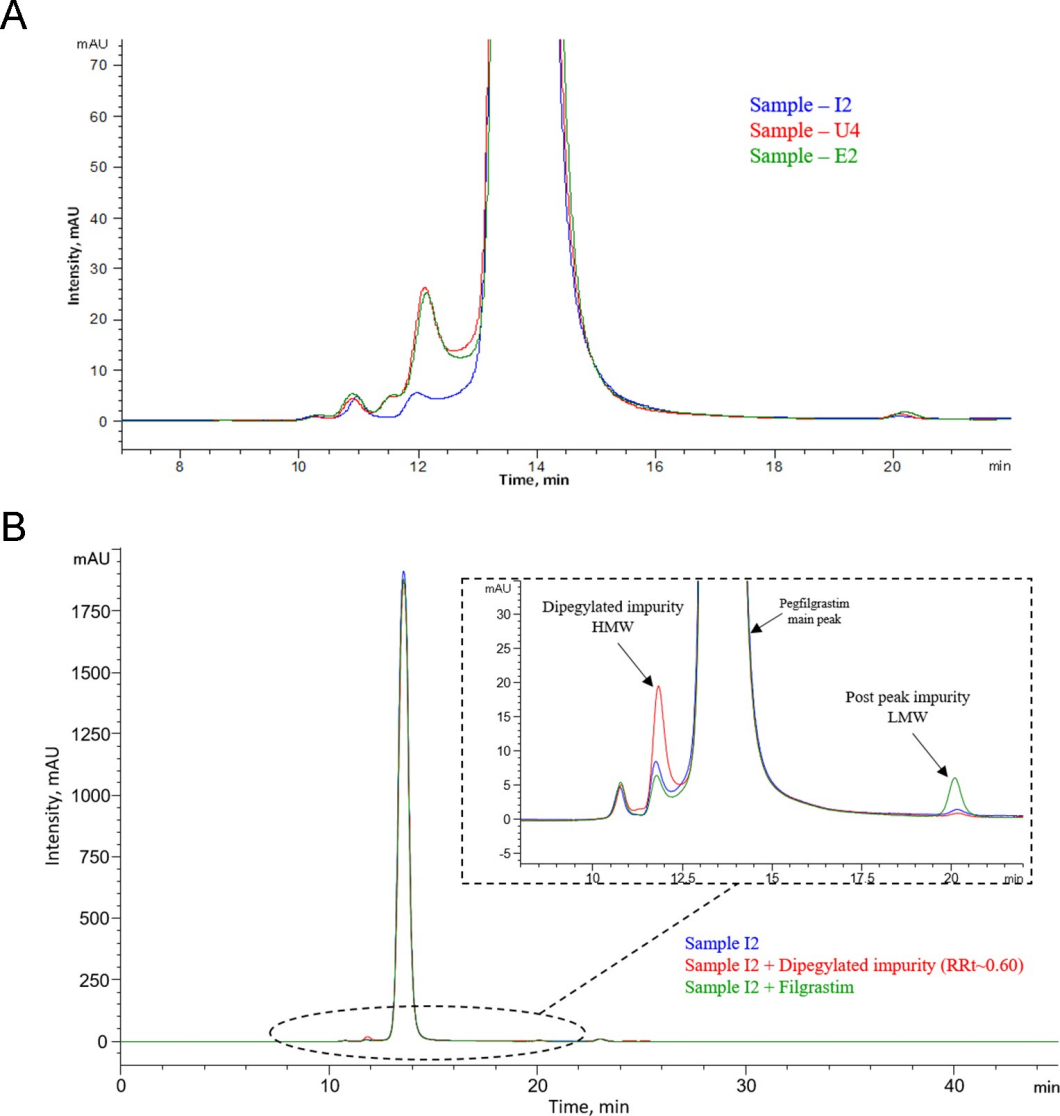
(A) Overlay of SEC-HPLC chromatographic profile of INTP5, US Neulasta and EU Neulasta (Sample I2, U4 and E2) (B) SEC-HPLC profile of spiking Low Molecular weight (Filgrastim, F1) (green) and High Molecular Weight (purified dipegylated filgrastim) (Red) impurities in INTP5.

A representative SEC-HPLC profile obtained for INTP5 is provided in Fig 14(A). Based on the literature [18, 22] higher molecular weight impurities (Pre-peak impurities RRT about 0.83 and RRT about 0.87) could be dipegylated or multipegylated pegfilgrastim, dimer, oligomers and aggregated impurities while the lower molecular weight impurity (Post peak impurity RRT about 1.47) could be free filgrastim. Impurities observed in SEC are in very low quantity (Table 7) hence the identification of these impurities is done by spiking known impurities in control samples. The SEC-HPLC overlay chromatogram of spiked dipegylated impurity (Met1 and Lys41 dipegylated) (Fig 14(B)) shows an increase in the height of the pre-peak impurity (RRT about 0.87) in the spiked sample with respect to the INTP5 control sample, which suggests that the dipegylated pegfilgrastim impurity elutes at RRT about 0.87 in SEC-HPLC. Spiking of free filgrastim into INTP5 control sample shows increase of the post-peak impurity (RRT ~ 1.47) in spiked samples with respect to the control sample which suggest that the low molecular weight impurity, free filgrastim, elutes at RRT~ 1.47 in SEC-HPLC (Fig 14 (B)).

**Table 7:** Quantitative purity analysis of INTP5, US Neulasta and EU Neulasta using SEC-HPLC.

SE-HPLC (Method LOD (Limit of Detection) is 0.05% and LOQ (Limit of Quantitation) is 0.1% of 36 μg)				
Sample Name		INTP5 (n = 16)	US Neulasta (n = 18)	EU Neulasta (n = 11)
% Main peak	Average	99.6	98.2	98.1
	Range (Min-Max)	99.3–99.7	97.6–98.6	97.5–98.3
% Total impurities	Average	0.4	1.8	1.9
	Range (Min-Max)	0.3–0.7	1.4–2.5	1.7–2.5

**Fig 15:**
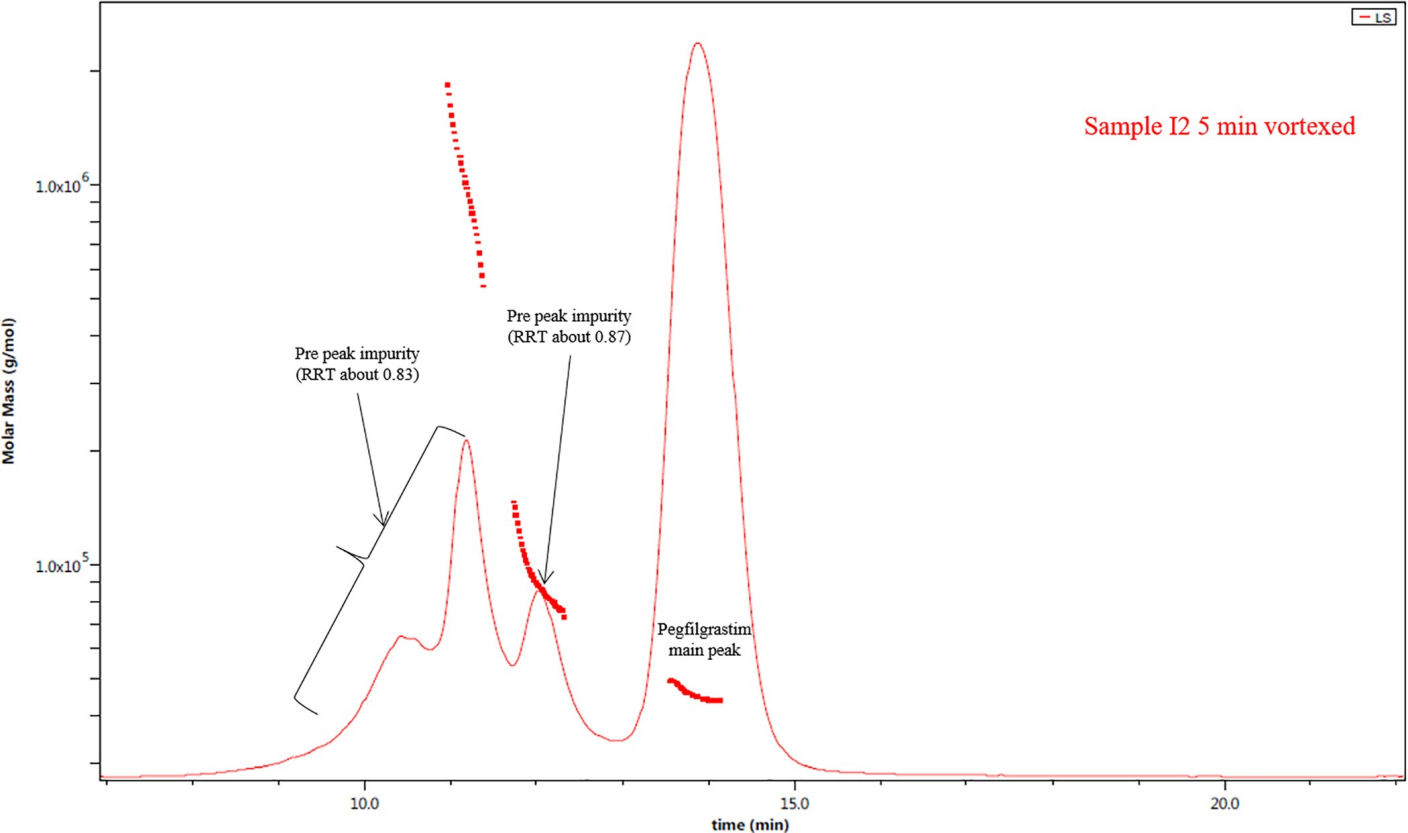
Light scattering profile of vortexed INTP5 (molar mass vs. time).

Additionally, the identification of pre-peak impurities was done using SEC-MALS of stress aggregated sample. An increase in impurity content at RRT about 0.83 and RRT about 0.87 was observed after vortexing (S23 Fig and Table J in S1 Appendix). The SEC-MALS data of RRT about 0.87 species in the vortexed samples of pegfilgrastim had a molecular weight of 91.4 kDa while the main peak molecular weight is 45.6 kDa [22, 23]. This indicates that the RRT ~ 0.87 impurity is a dimer of pegfilgrastim. The RRT ~ 0.83 peak molecular weight is 945 kDa indicating the presence of higher order aggregates (Fig 15 and Table 8).

**Table 8:** Determination of Molecular Weight by SEC-MALS.

Sample name	Principal peak		RRT about 0.87		RRT about 0.83*	
	(kDa)	Rt (min)	(kDa)	Rt (min)	(kDa)	Rt (min)
**INTP5 (vortexed)**	45.6	13.711	91.4	11.943	945.0	11.684

Note:

‘*’ The peak at about 11.12 min shows high scattering with a broad peak profile co-eluting with the 11.684 min peak suggesting multiple species eluting together. Appropriate molecular weight is not determined as multiple peaks were eluted together.

**Fig 16:**
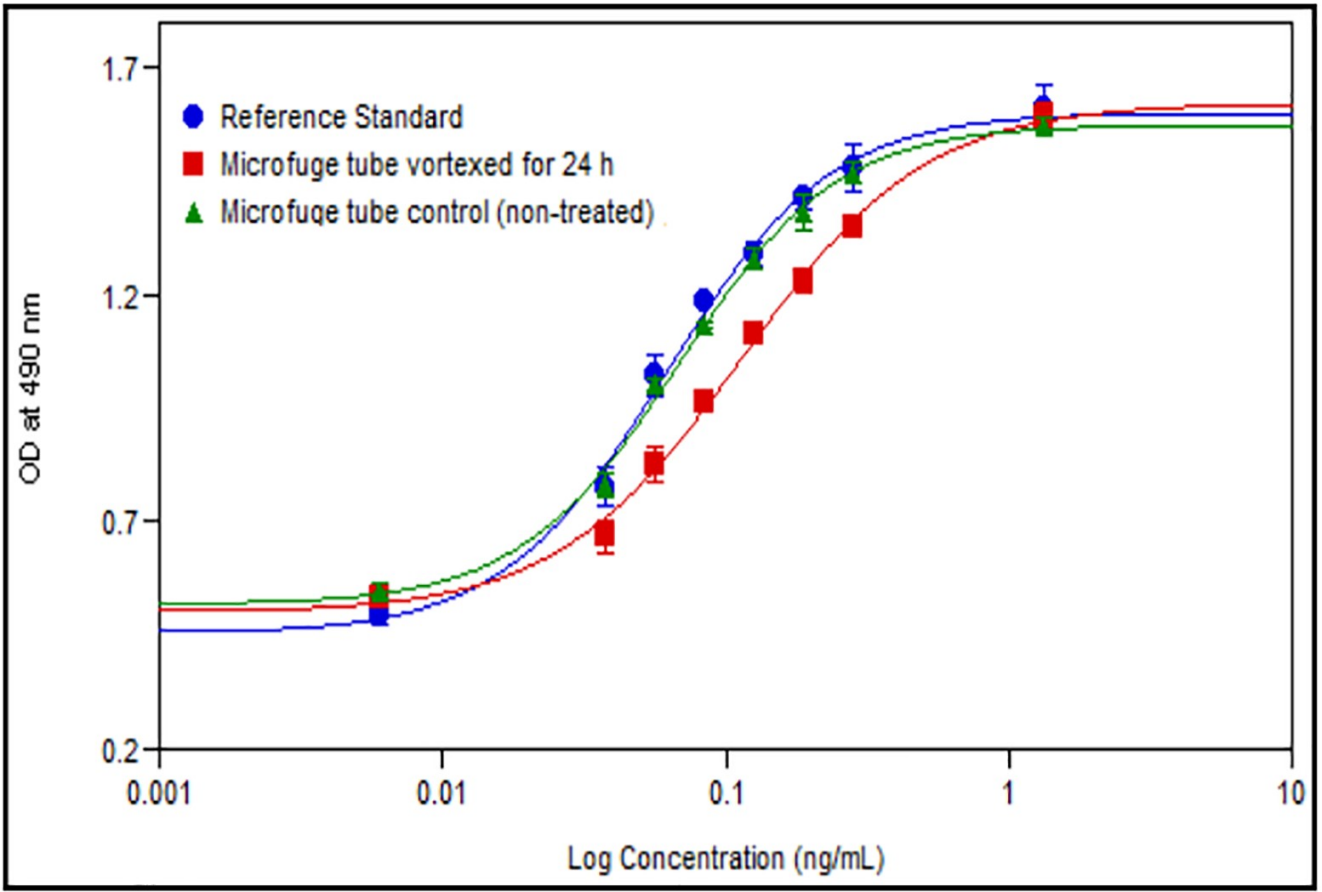
Representative profile for *in vitro* bioassay of INTP5 control (microfuge tube) and vortexed (stressed) samples.

To assess the impact of high molecular weight species (HMWs) on biological activity, stress sample was generated by vortexing the sample (Sample I2). The SEC-HPLC profile indicated that species generated were in this experiment is qualitatively same as shown in S24 Fig. The *in vitro* bioassay data (Fig 16 and Table 9) of stress aggregated sample (sample with about 60.4% HMW impurities) shows significant reduction in biological activity. This data indicates that most of these HMW impurities are biologically inactive.

**Table 9:** *In vitro* bioassay and SEC-HPLC data of forced aggregated samples.

Sample Name	% Total aggregates (RRT about 0.75 to 0.83)	% Dipegylated Filgrastim/ dime (RRT about 0.87)	% Free Filgrastim (RRT about 1.47)	% Largest Undefined peaks impurities	% Total Impurities	% Purity	% Undefined peaks impurities	% Relative Potency
Control INTP5	<LOQ (0.1%)	0.3	<LOQ (0.1%)	ND	0.3	99.7	ND	94
Stressed (vortexed) INTP5	39.9	20.5	<LOQ (0.1%)	ND	60.4	39.6	ND	53

All high molecular weight impurities, including dimers and dipegylated species were not active in the *in vitro* potency assay. Considering this, it is essential to control the size based impurities during the production process. As observed from the data in Table 7, these variants are well controlled in the INTP5 at levels below 2.0%.

## Conclusion

This study demonstrated the structural and functional similarity of INTP5 with Neulasta using various analytical techniques and functional assays. The pegylation of filgrastim involves chemical reaction of mPEG-PAL to filgrastim. The products of the pegylation reaction were thoroughly characterized and seen to consist of pegylation largely at the N-terminus, with a low percentage of pegylation at Lys residues which results into dipegylated species and positional isomers. The final purified pegfilgrastim was found to consist largely (> 98%) of mono-pegylated filgrastim with the PEG moiety attached to the N-terminal Met, in all three products tested–INTP5, EU and US sourced Neulasta. The linkage of the PEG to the N-terminus was confirmed by LC MS and NMR methodologies. In addition to the process generated variants, the product related variants- oxidized, deamidated, reduced, non-pegylated filgrastim, dimers and higher order aggregates were also characterized both at the molecular level and by assessing their impact using *in vitro* cell based potency assay. The study also confirmed that the impurity detection methods of CEx, RP and SEC-HPLC together provide a comprehensive resolution of all expected product variants and serve as important methods to monitor product quality routinely. A comprehensive summary of impurity characterization by various HPLC methods and its correlation to potency is presented in Table K in S1 Appendix. Extensive product characterization is of significance in biopharmaceutical manufacturing as it further helps in lifecycle management of the product. An in depth scientific understanding of the product quality attributes, enables a better design of process controls and ensures appropriate product quality monitoring during routine production and stability studies.

The pegylated filgrastim biosimilar produced at Intas has the same types of product variants as observed in the innovator product (Neulasta), and at slightly lower levels, providing assurance that the safety and efficacy of the biosimilar product would be similar to that of the innovator product. Biosimilars are lower cost, but equally safe and effective versions of the originator biologics and extensive evidence of scientific data are required by regulatory authorities for the demonstration of similarity. The in-depth structural characterization of the Intas pegfilgrastim provides a strong base for the demonstration of biosimilarity of the product.

## Supporting information

S1 Fig Identification of pegylation site by LC-ESI-MS, I1 INTP5. (TIF)

S2 Fig Identification of pegylation site by LC-ESI-MS, U1 (US Neulasta). (TIF)

S3 Fig Spectral comparison the aliphatic region of PEG-filgrastim N-terminal peptide samples. (Note: samples 1 to 3 are for INTP5 (I1, I2 and I3) and samples 4 to 9 for Neulasta (U1, E1, E2, U2, U3 and E3).
(TIF)

S4 Fig SDS-PAGE analysis of INTP5- I2 DP CEX impurity peaks. (Note: Aggregated and higher molecular weight impurities were also observed which indicates the possibility of dipegylated or multipegylated species in impurity fractions. There is also a possibility that some of the aggregates observed are generated during the process of impurity collection and concentration. Solution 1, Solution 4, Solution 5, Solution 6 and Solution 7 are the internal Reference Standard solutions.).
(TIF)

S5 Fig TIC overlay of pegfilgrastim main peak and Pre-peak impurity (RRT 0.91) in peptide mapping (10–75 min). (TIF)

S6 Fig TIC overlay of pegfilgrastim main peak and Pre-peak impurity (RRT 0.91) in peptide mapping (75–145 min). (TIF)

S7 Fig TIC overlay of pegfilgrastim main peak and Pre-peak impurity (RRT 1.15) in peptide mapping (10–75 min). (Note: Peaks eluted at retention time ~ 13.5 min in the pegfilgrastim main peak and ~ 15.5 min in Post-peak impurity RRT 1.15 are the same peptide–QRRAGGVL (146–153)).
(TIF)

S8 Fig TIC overlay of pegfilgrastim main peak and Pre-peak impurity (RRT 1.15) in peptide mapping (75–145 min). (TIF)

S9 Fig TIC overlay of pegfilgrastim main peak and Pre-peak impurity 2 (RRt- 0.73) in peptide mapping (10-75min). (TIF)

S10 Fig TIC overlay of pegfilgrastim main peak and Pre-peak impurity 2 (RRt- 0.73) in peptide mapping (75–145 min). (TIF)

S11 Fig TIC overlay of pegfilgrastim main peak and Pre-peak impurity 2 (RRt- 0.73) in Endoproteinase Glu-C peptide mapping (10–60 min). (Note: N-terminal pegylated peptide was diverted from the LC-MS as it contaminates the Triple TOF instrument. It is present in UV spectra in both Pre-peak impurity and Pegfilgrastim main peaks suggesting pegylation at M1 and L35 position.)
(TIF)

S12 Fig TIC overlay of pegfilgrastim main peak and Pre-peak impurity 3 (RRt- 0.84) in peptide mapping (10 min -75 min). (TIF)

S13 Fig TIC overlay of pegfilgrastim main peak and Pre-peak impurity 3 (RRt- 0.84) in peptide mapping (75–145 min). (Note: N-terminal pegylated peptide was diverted from the LC-MS as it contaminates the Triple TOF instrument. It is present in UV spectra in both the pre-peak impurity and the pegfilgrastim main peak suggesting Pegylation at M1 and K17/K24/K35 position.)
(TIF)

S14 Fig Dose response curves for *in vitro* biological activity of pre-peak impurities 1, 2 and 3. (TIF)

S15 Fig TIC overlay of INTP5 control and oxidized impurity 2 (RRt about 0.86) in peptide mapping (10–54 min). (TIF)

S16 Fig TIC overlay of INTP5 control and oxidized impurity 3 (RRt ~0.91) in peptide mapping (10–54 min). (TIF)

S17 Fig TIC overlay of INTP5 control and oxidized impurity 4 (RRt about 0.96) in peptide mapping (10–54 min). (TIF)

S18 Fig TIC overlay of pegfilgrastim main peak and Post-peak impurity (RRt about 1.06) in peptide mapping (10–60 min). (TIF)

S19 Fig TIC overlay of pegfilgrastim main peak and Post-peak impurity (RRt 1.06) in peptide mapping (60–85 min). (TIF)

S20 Fig TIC overlay of pegfilgrastim main peak and Post-peak impurity (RRt 1.23) in peptide mapping (60–85 min). (TIF)

S21 Fig Dose response curves for *in vitro* biological activity of Post-peak impurity (RRt 1.06) and Post-peak impurity (RRt 1.23). (TIF)

S22 Fig RP-HPLC profile of various impurities spiked in purified product (Sample I2). (TIF)

S23 Fig SEC-HPLC chromatogram of vortexed INTP5 (Sample I2). (TIF)

S24 Fig SEC-HPLC chromatogram of 24 hours vortexed INTP5 (Sample I2) in microfuge tube. (TIF)

S1 Appendix Supporting information–tables. (DOCX)
