# Supplementary figures and images for: Structural similarity, characterization of Poly Ethylene Glycol linkage and identification of product related variants in biosimilar pegfilgrastim
Source: PLoS One. 2019 Mar 13;14(3):e0212622. doi: 10.1371/journal.pone.0212622 (PMC6415886; doi:10.1371/journal.pone.0212622)

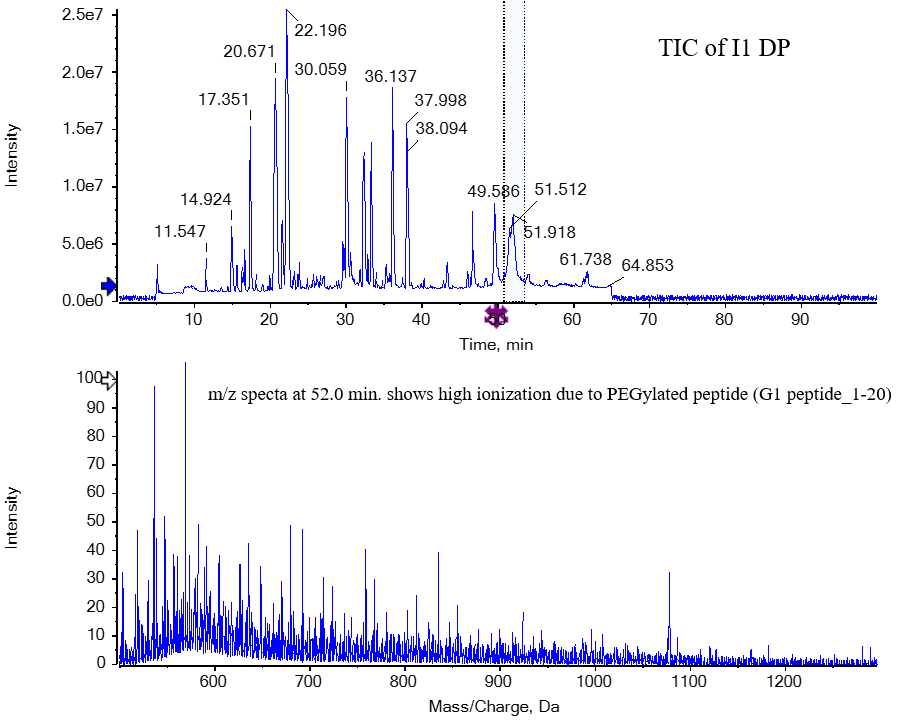

Supplement: S1 Fig — (TIF) [file pone.0212622.s001.tif]

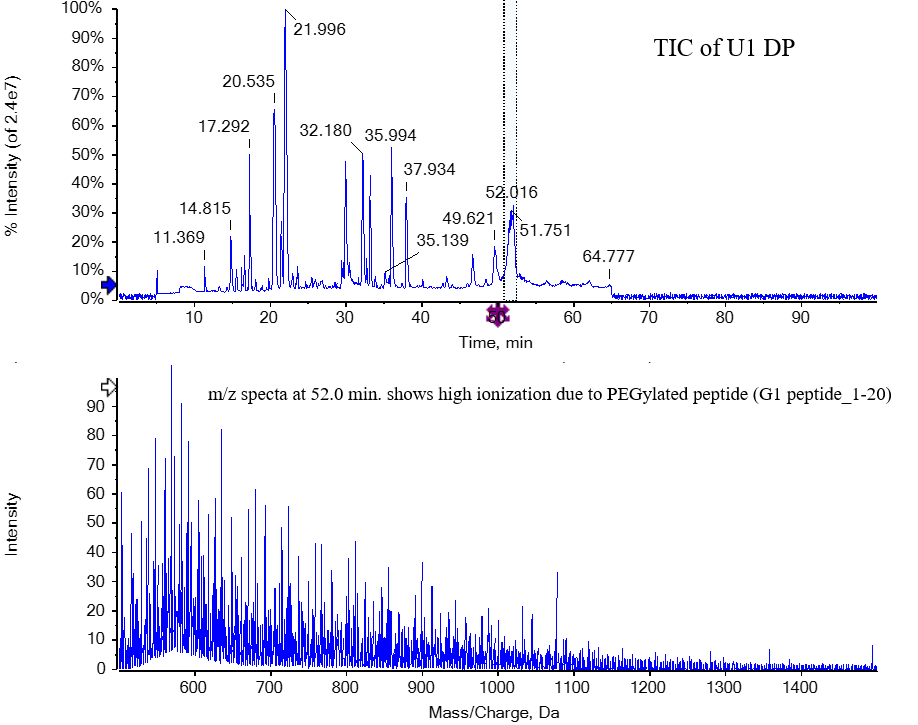

Supplement: S2 Fig — (TIF) [file pone.0212622.s002.tif]

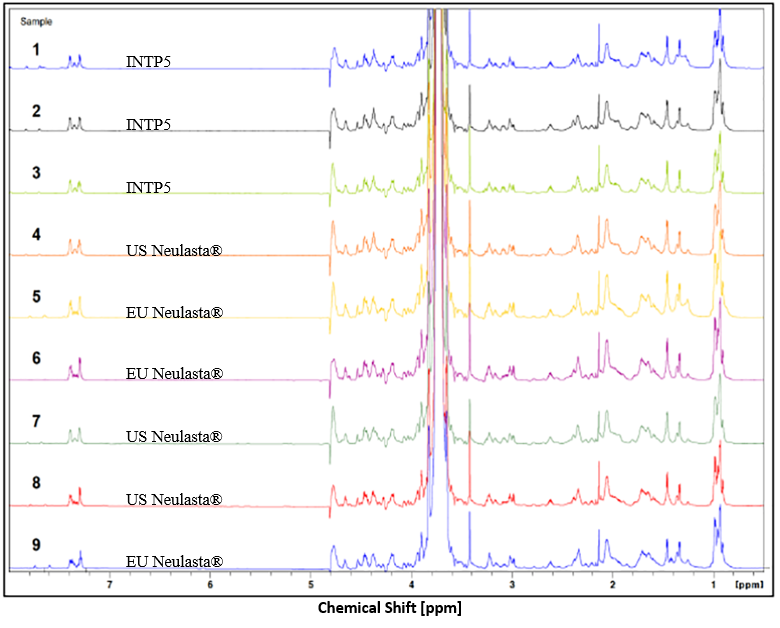

Supplement: S3 Fig — (Note: samples 1 to 3 are for INTP5 (I1, I2 and I3) and samples 4 to 9 for Neulasta (U1, E1, E2, U2, U3 and E3). (TIF) [file pone.0212622.s003.tif]

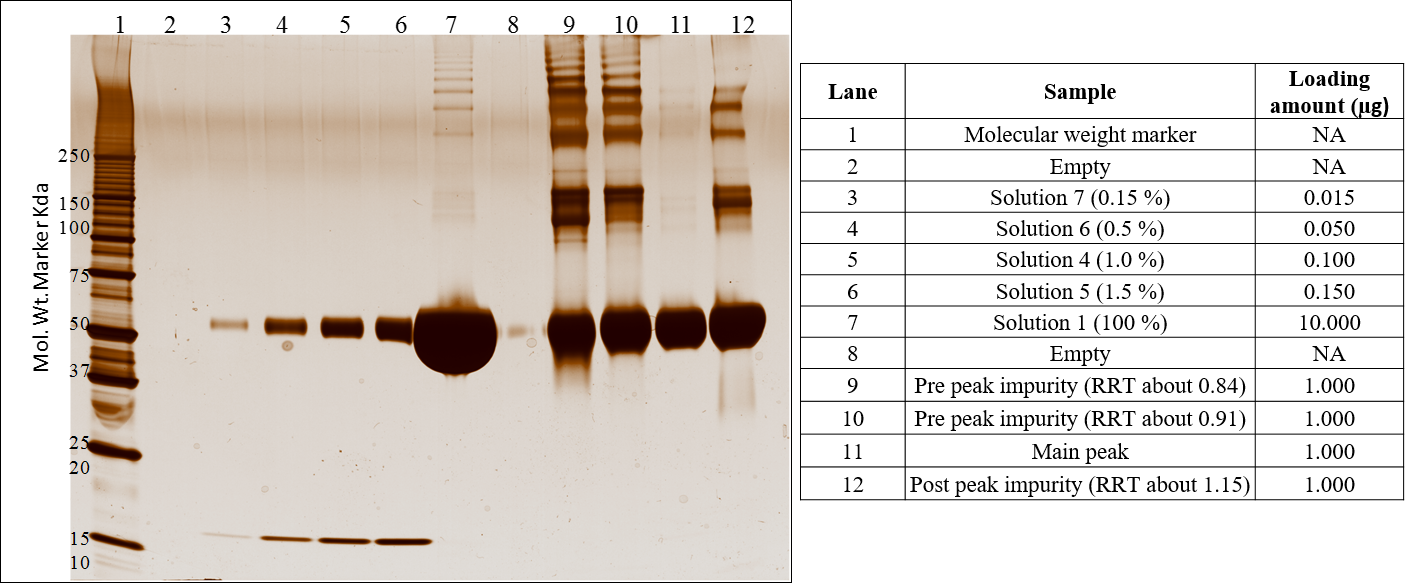

Supplement: S4 Fig — (Note: Aggregated and higher molecular weight impurities were also observed which indicates the possibility of dipegylated or multipegylated species in impurity fractions. There is also a possibility that some of the aggregates observed are generated during the process of impurity collection and concentration. Solution 1, Solution 4, Solution 5, Solution 6 and Solution 7 are the internal Reference Standard solutions.). (TIF) [file pone.0212622.s004.tif]

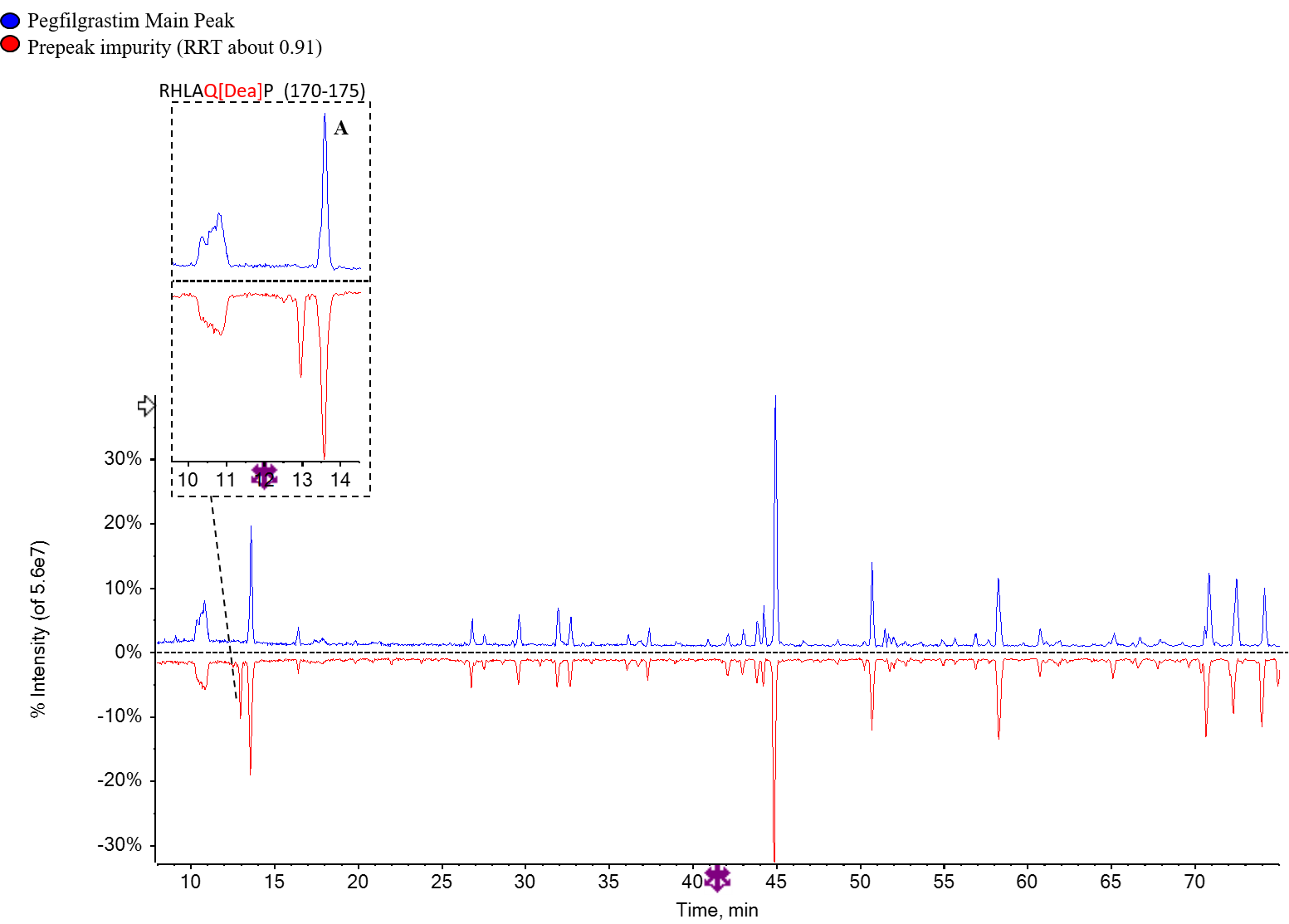

Supplement: S5 Fig — (TIF) [file pone.0212622.s005.tif]

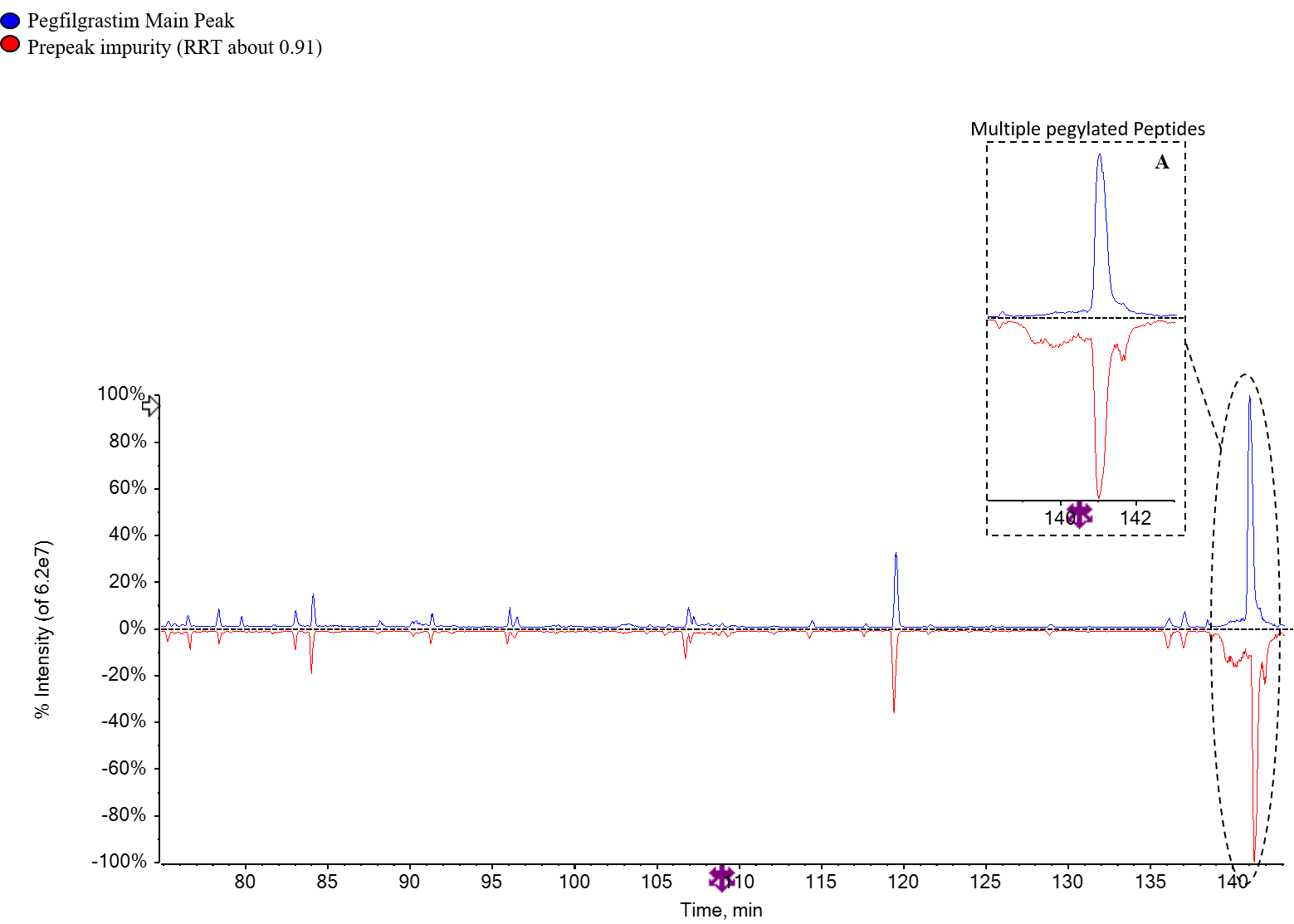

Supplement: S6 Fig — (TIF) [file pone.0212622.s006.tif]

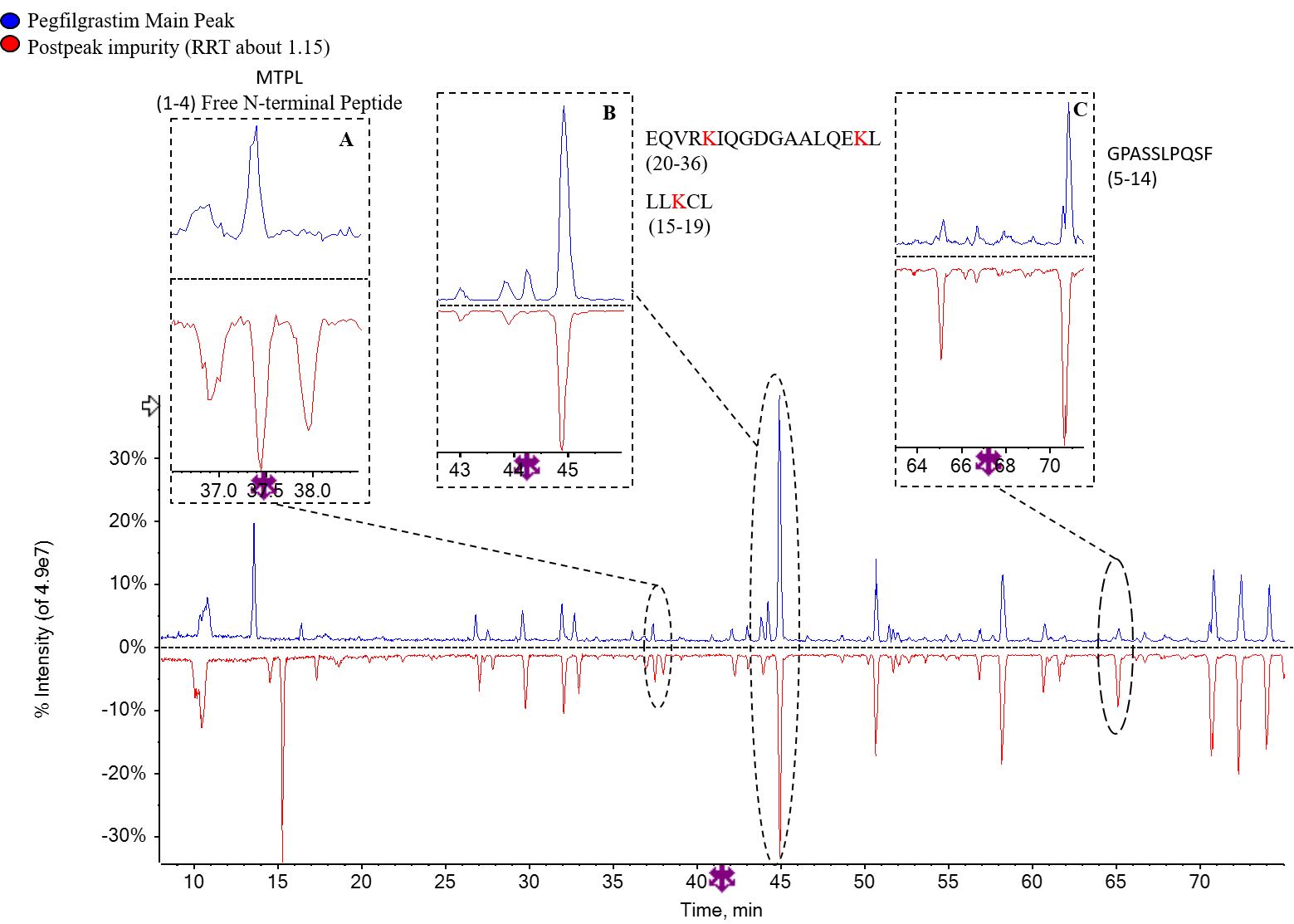

Supplement: S7 Fig — (Note: Peaks eluted at retention time ~ 13.5 min in the pegfilgrastim main peak and ~ 15.5 min in Post-peak impurity RRT 1.15 are the same peptide–QRRAGGVL (146–153)). (TIF) [file pone.0212622.s007.tif]

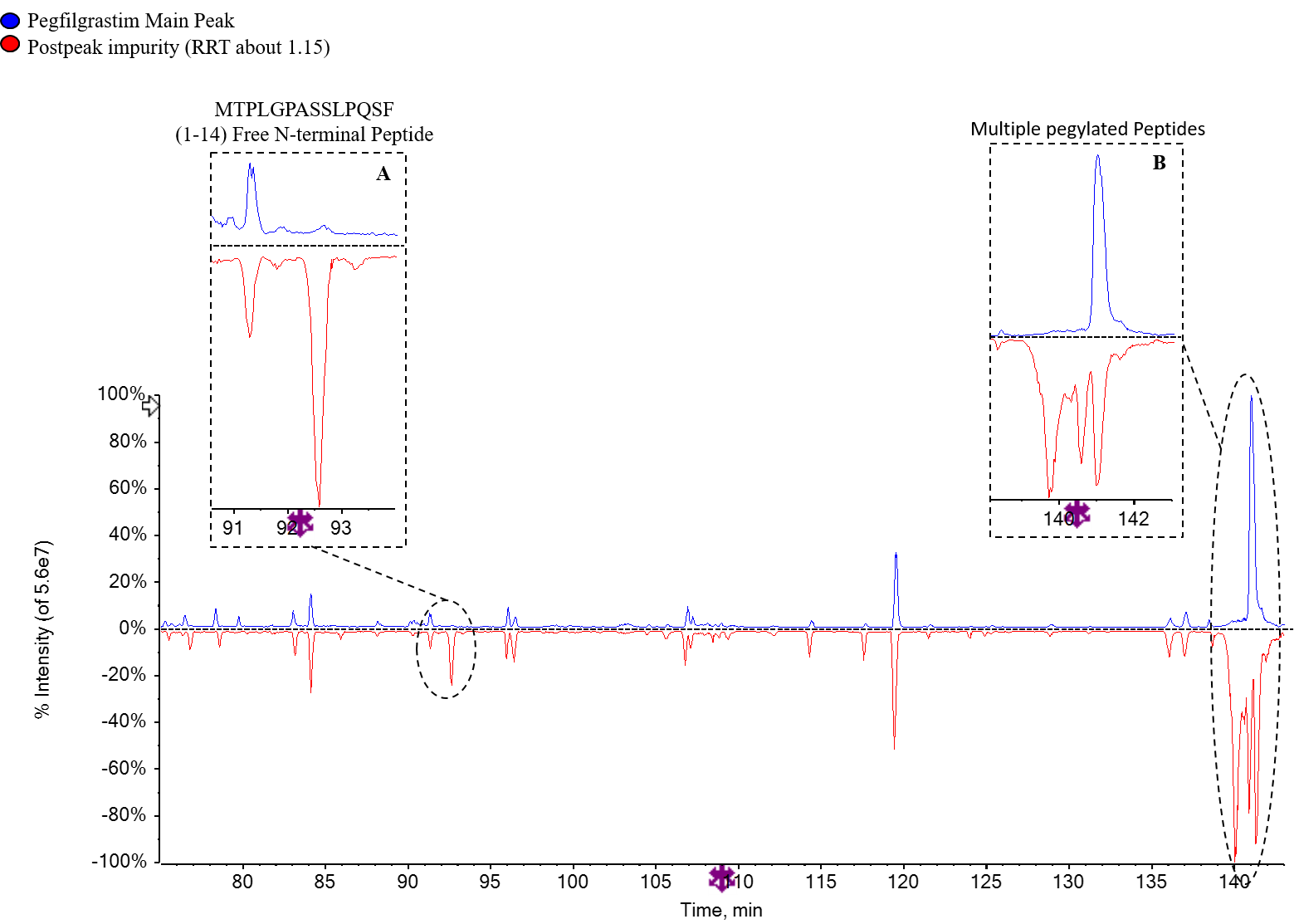

Supplement: S8 Fig — (TIF) [file pone.0212622.s008.tif]

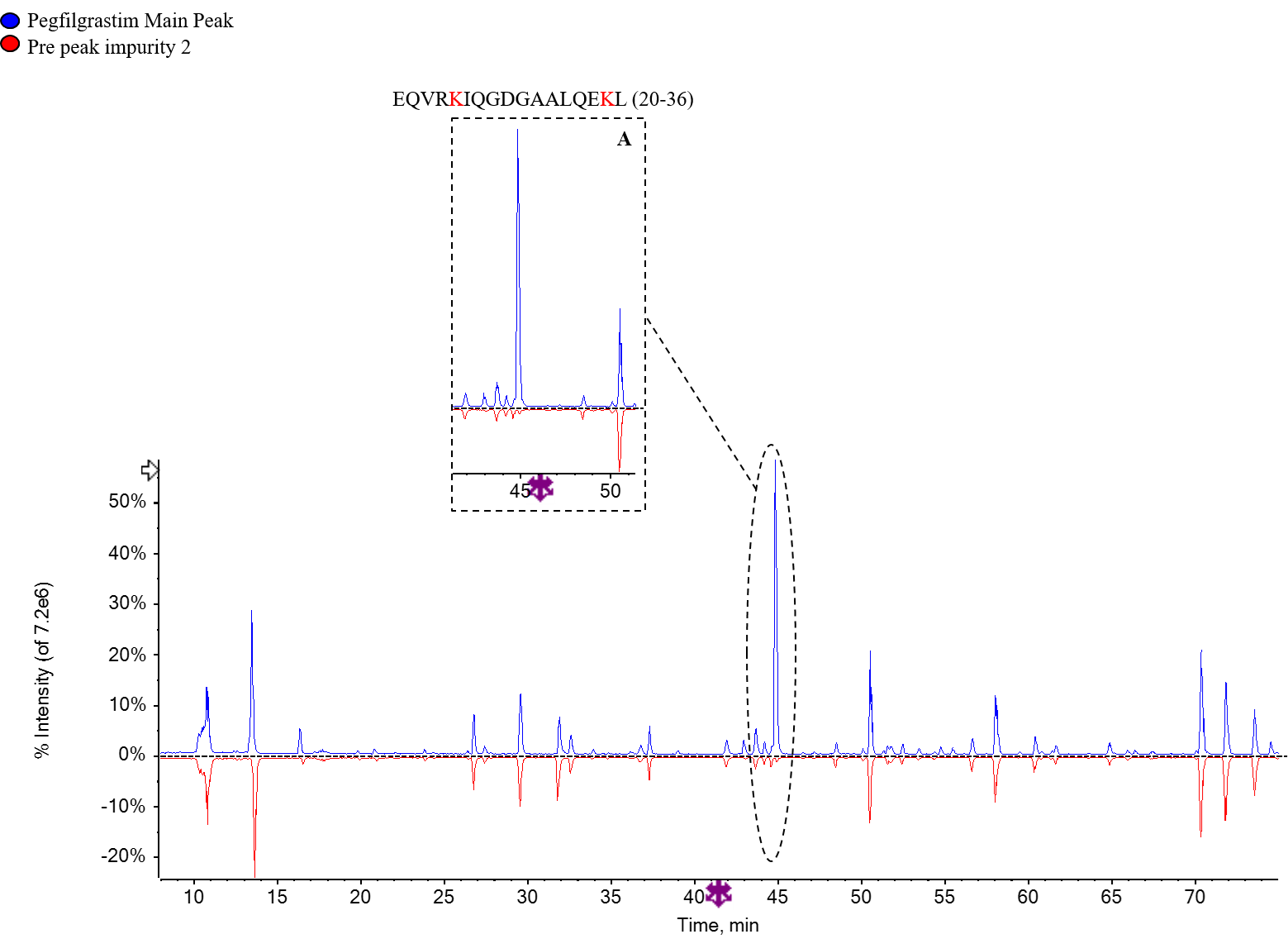

Supplement: S9 Fig — (TIF) [file pone.0212622.s009.tif]

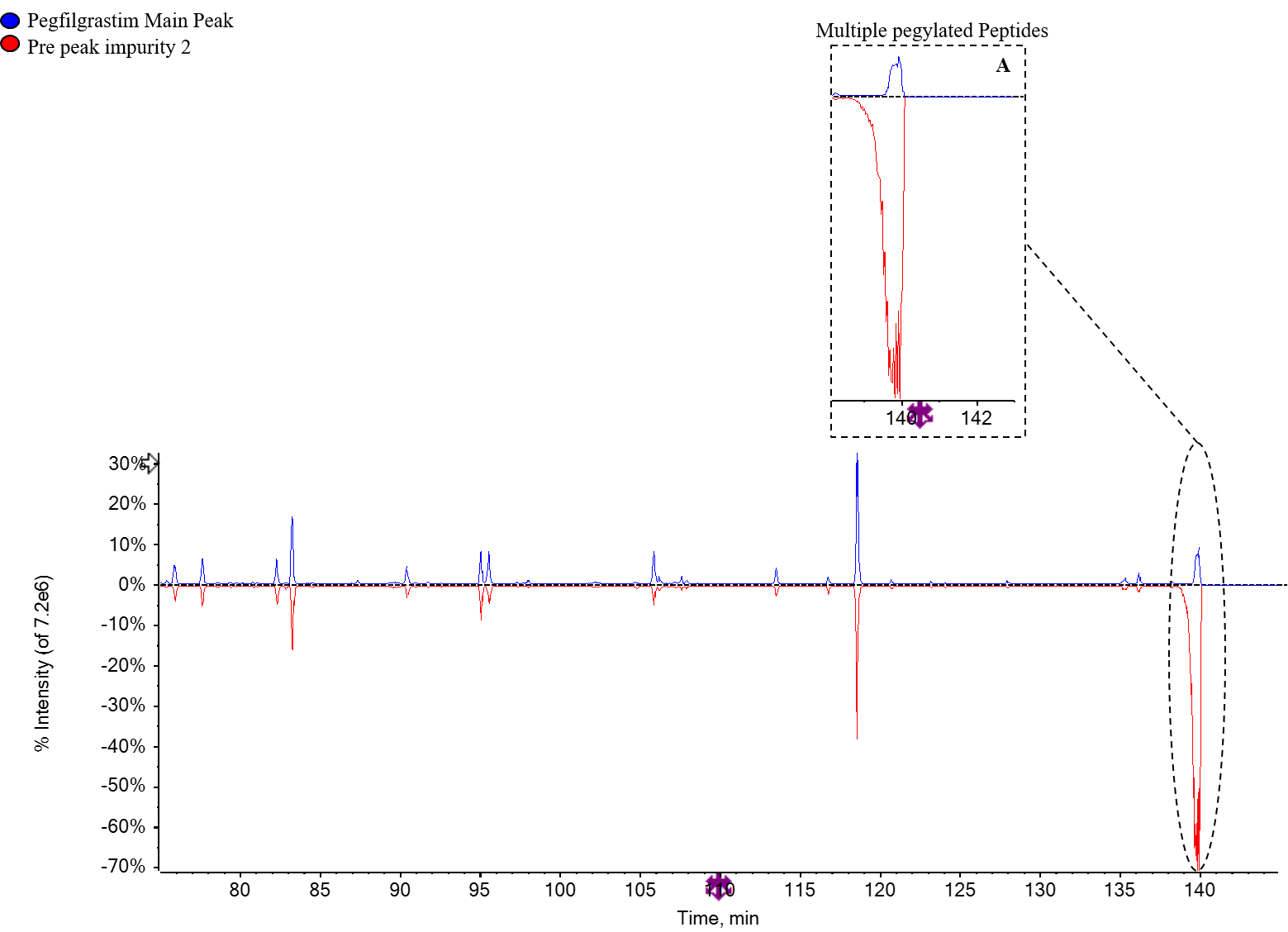

Supplement: S10 Fig — (TIF) [file pone.0212622.s010.tif]

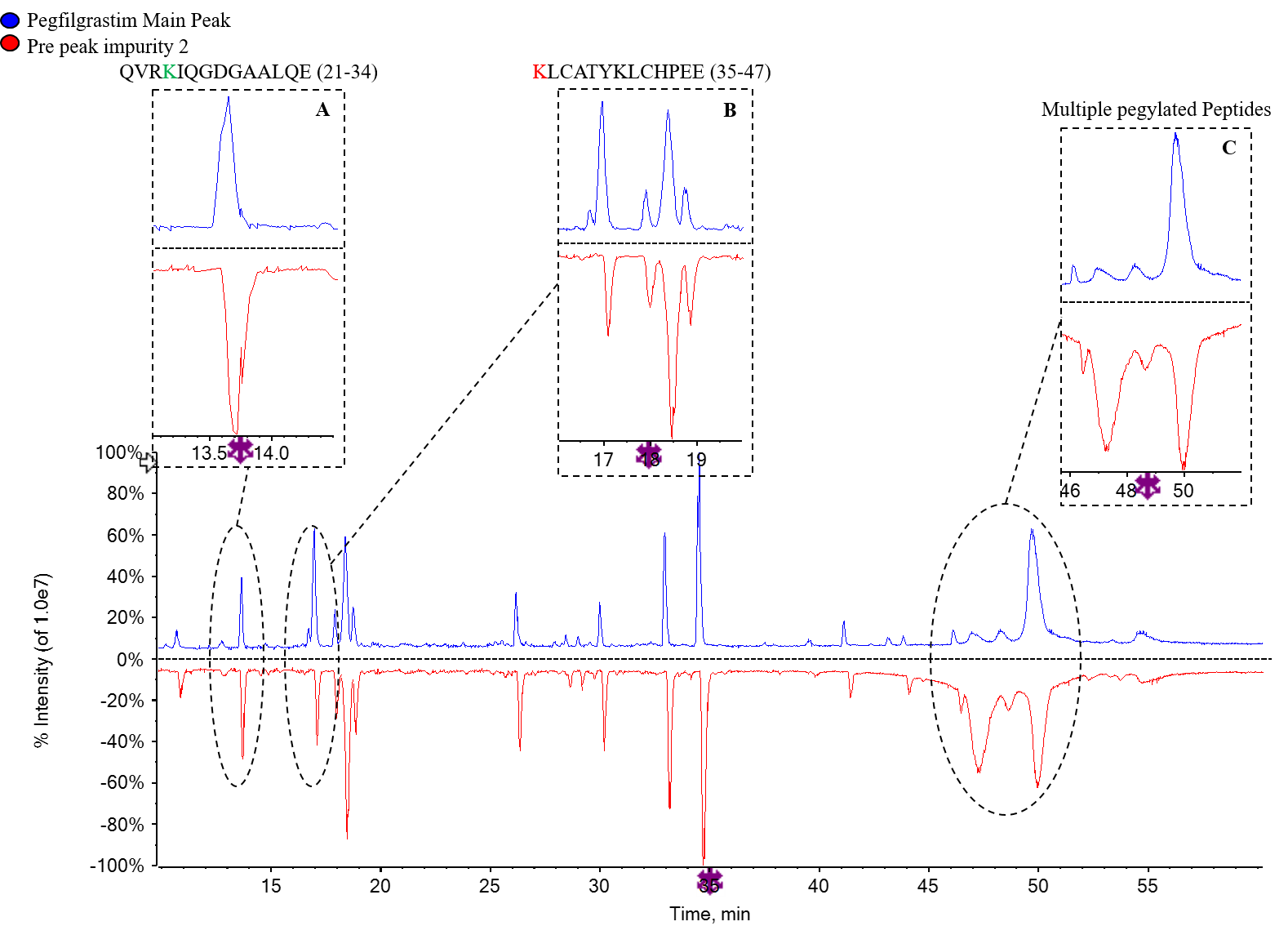

Supplement: S11 Fig — (Note: N-terminal pegylated peptide was diverted from the LC-MS as it contaminates the Triple TOF instrument. It is present in UV spectra in both Pre-peak impurity and Pegfilgrastim main peaks suggesting pegylation at M1 and L35 position.) (TIF) [file pone.0212622.s011.tif]

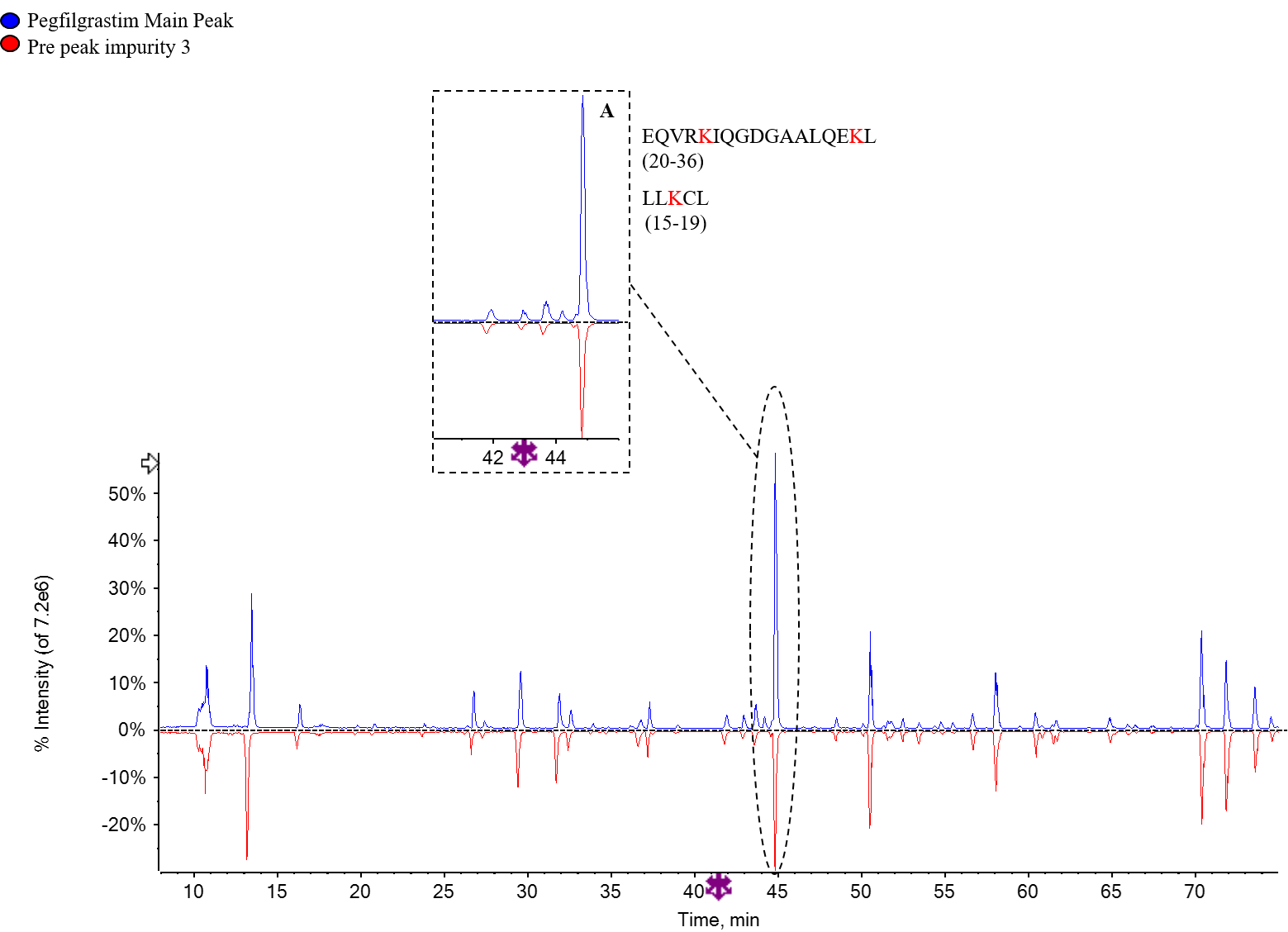

Supplement: S12 Fig — (TIF) [file pone.0212622.s012.tif]

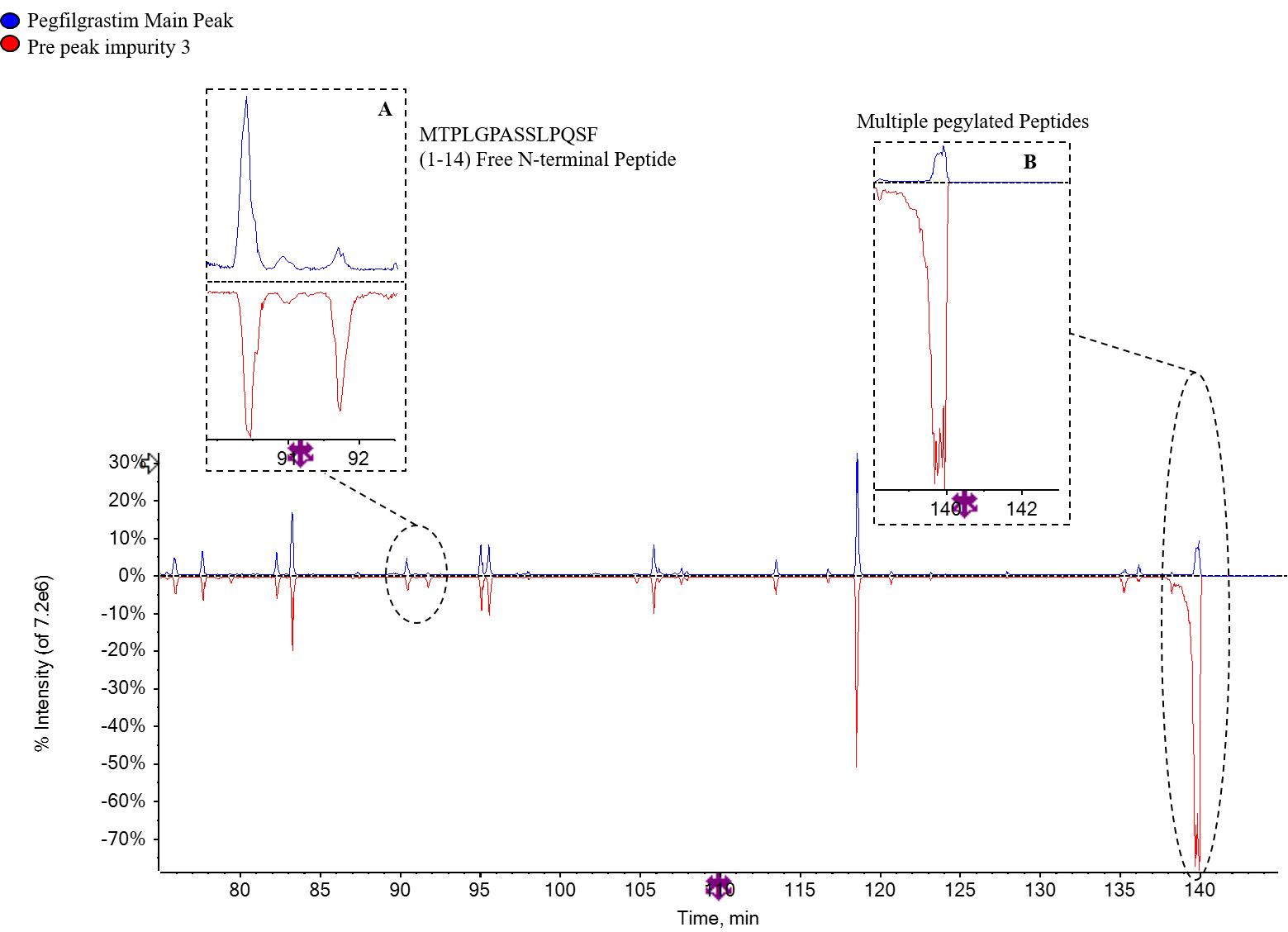

Supplement: S13 Fig — (Note: N-terminal pegylated peptide was diverted from the LC-MS as it contaminates the Triple TOF instrument. It is present in UV spectra in both the pre-peak impurity and the pegfilgrastim main peak suggesting Pegylation at M1 and K17/K24/K35 position.) (TIF) [file pone.0212622.s013.tif]

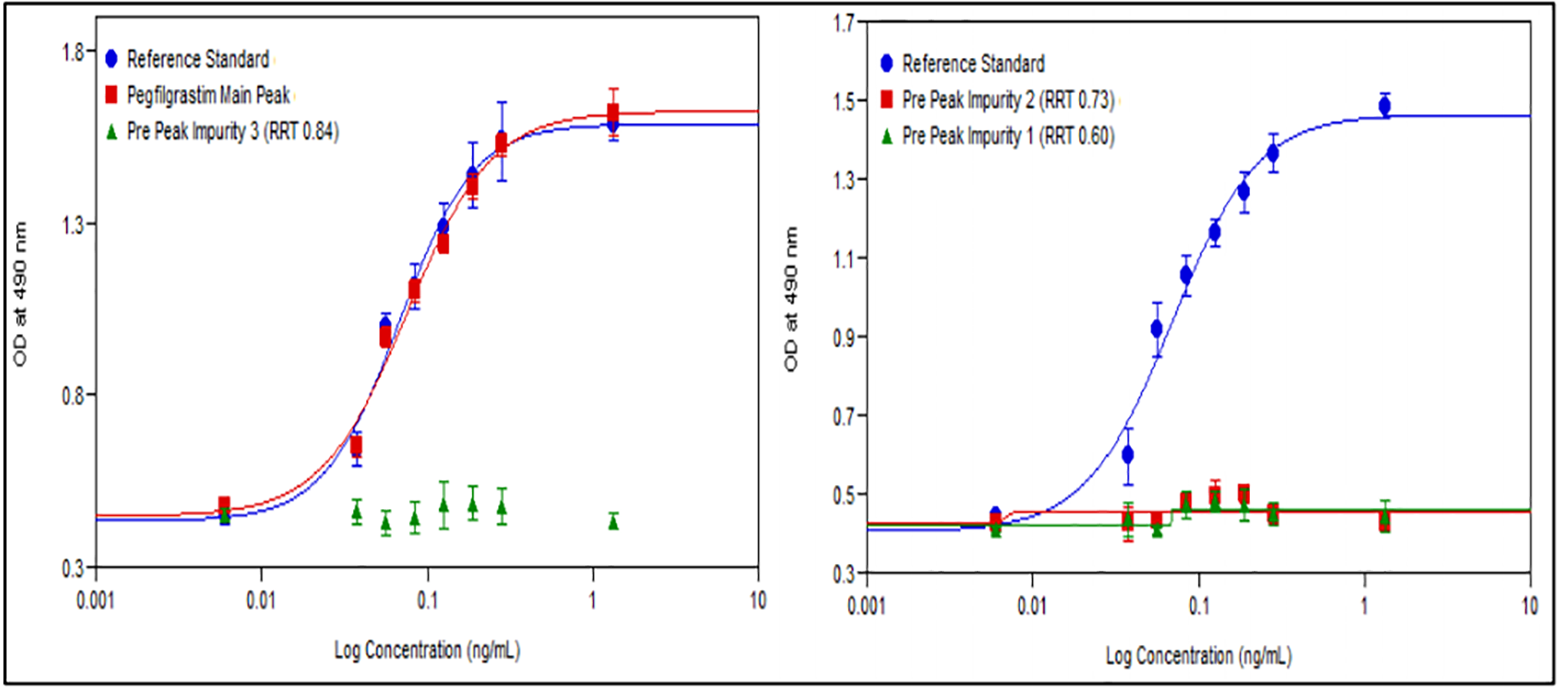

Supplement: S14 Fig — (TIF) [file pone.0212622.s014.tif]

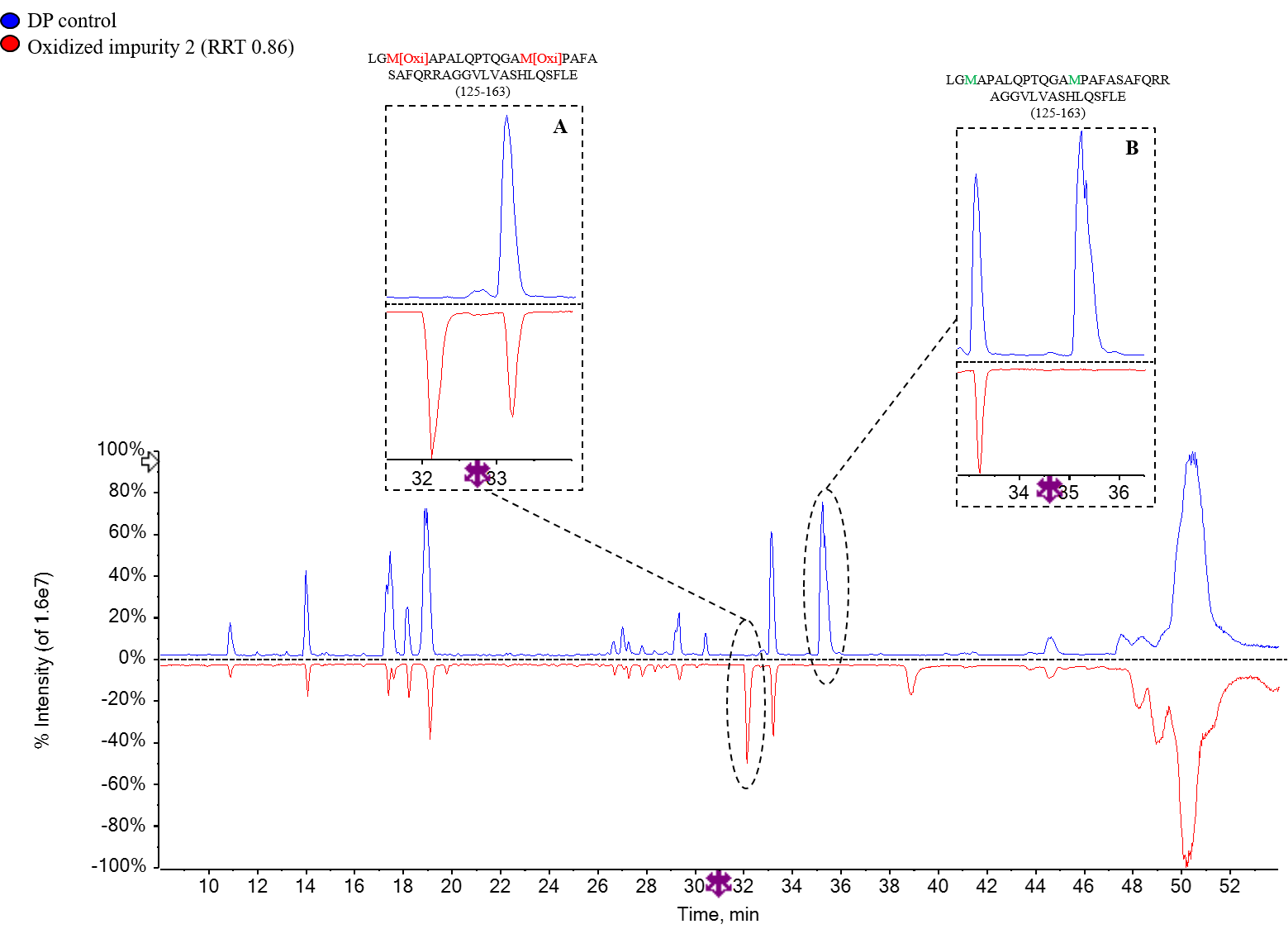

Supplement: S15 Fig — (TIF) [file pone.0212622.s015.tif]

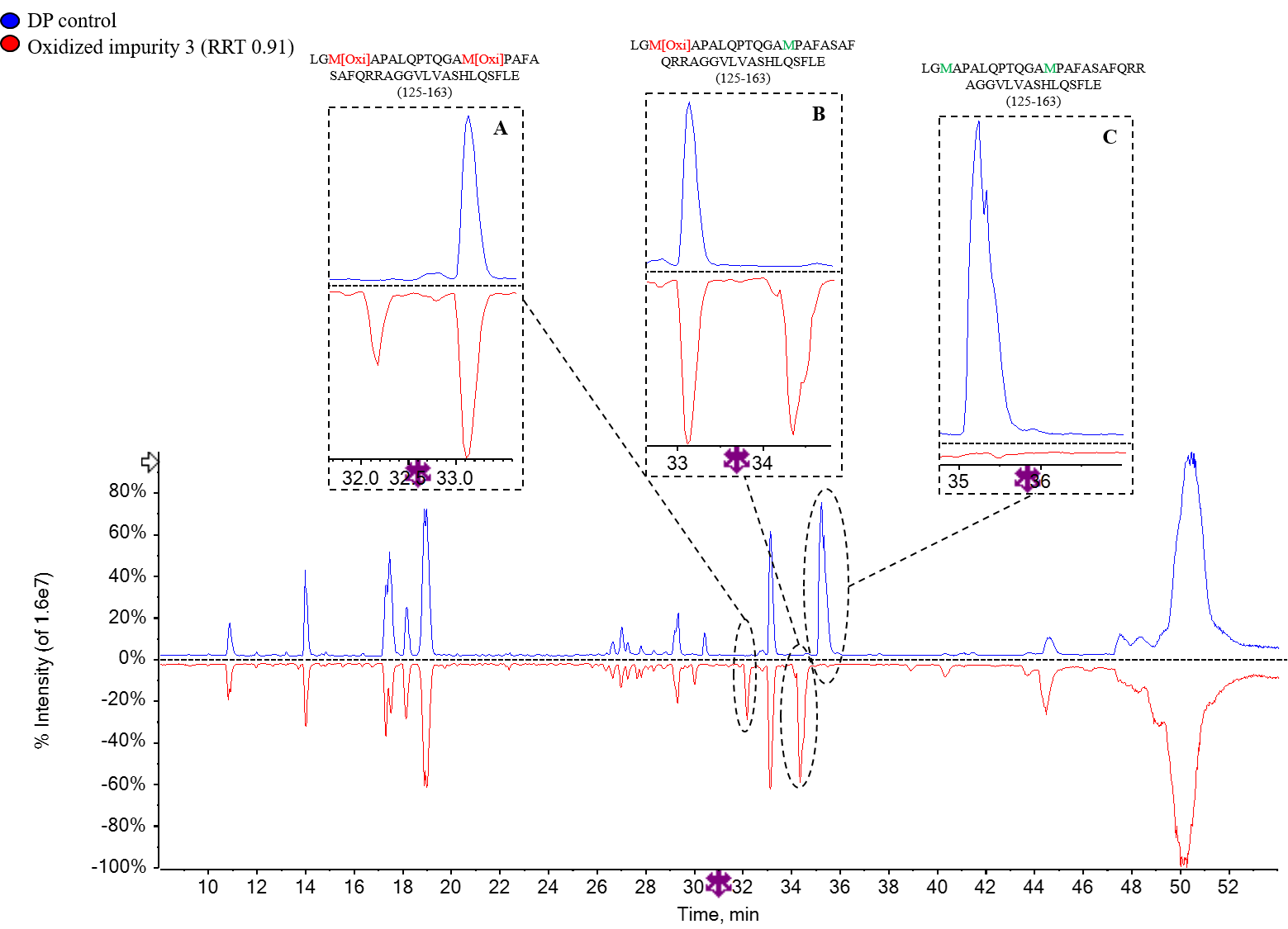

Supplement: S16 Fig — (TIF) [file pone.0212622.s016.tif]

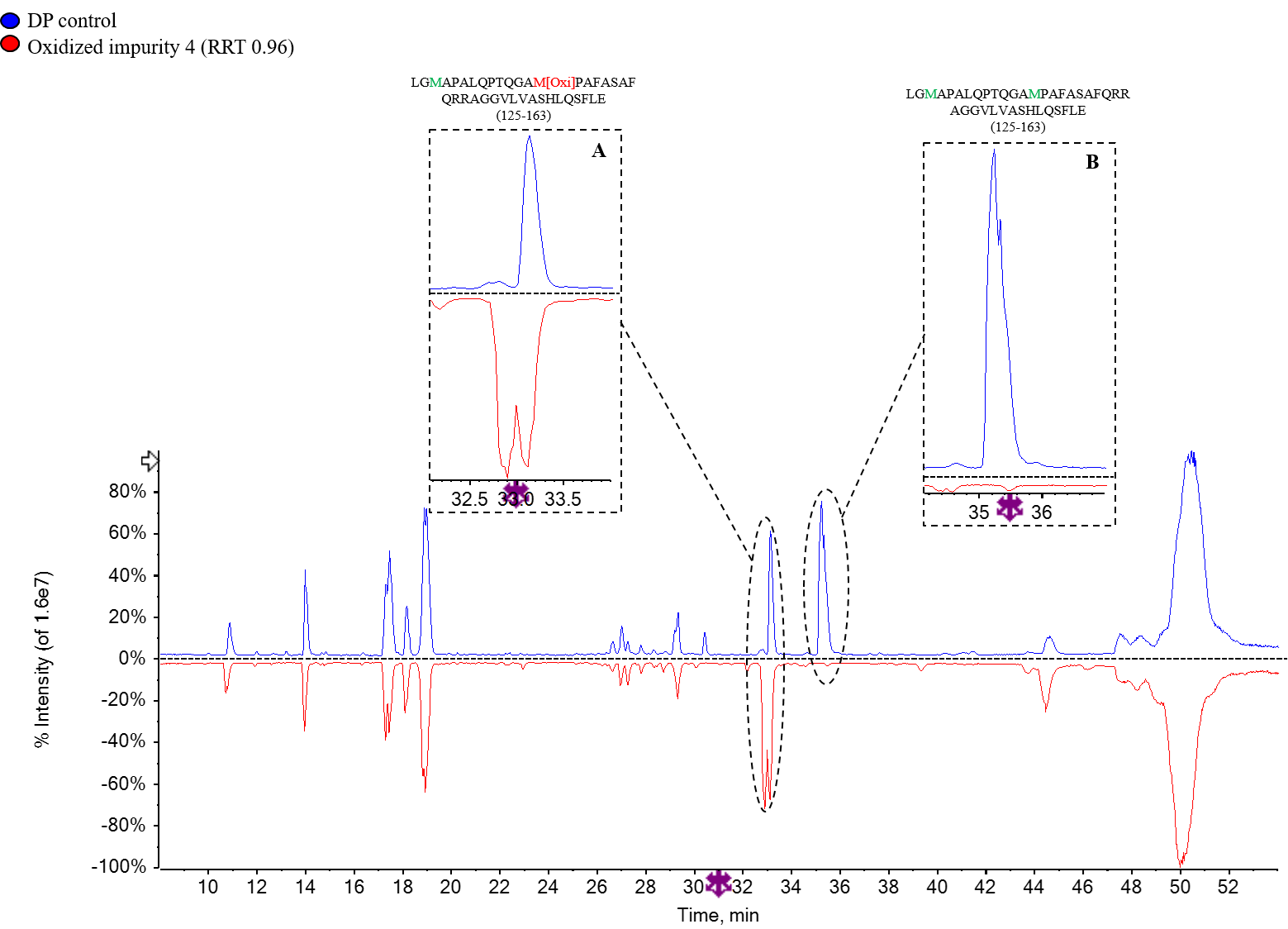

Supplement: S17 Fig — (TIF) [file pone.0212622.s017.tif]

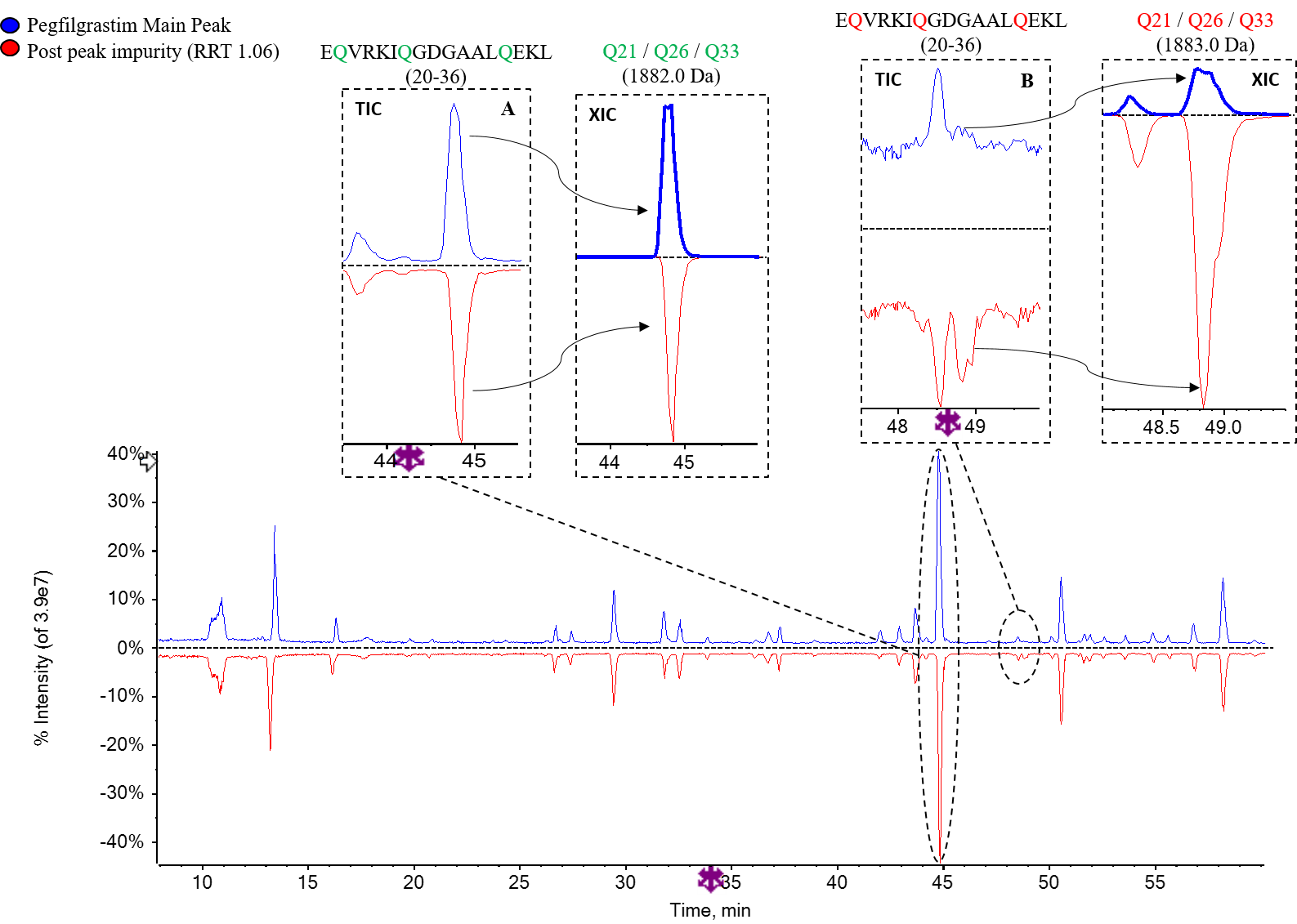

Supplement: S18 Fig — (TIF) [file pone.0212622.s018.tif]

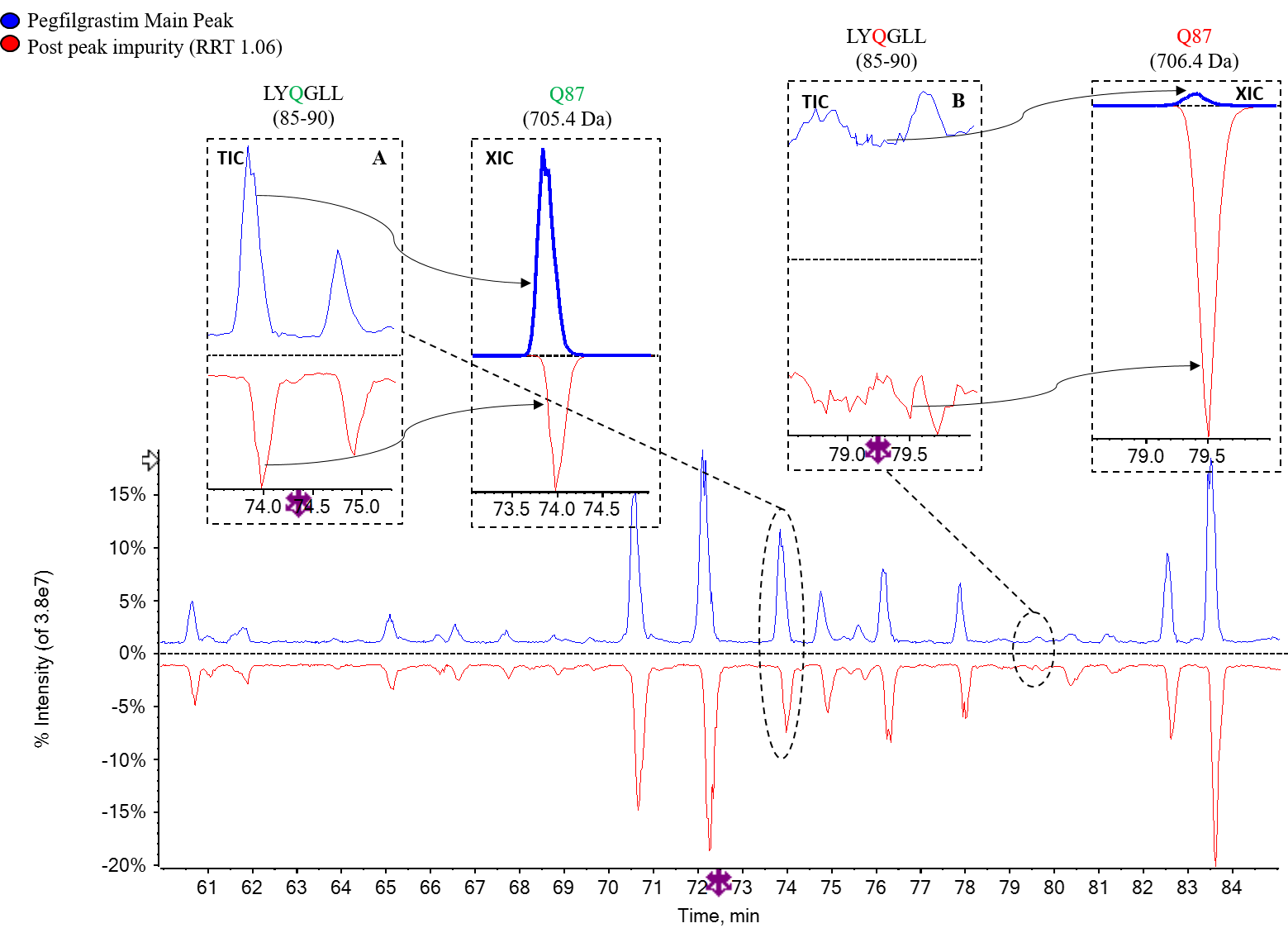

Supplement: S19 Fig — (TIF) [file pone.0212622.s019.tif]

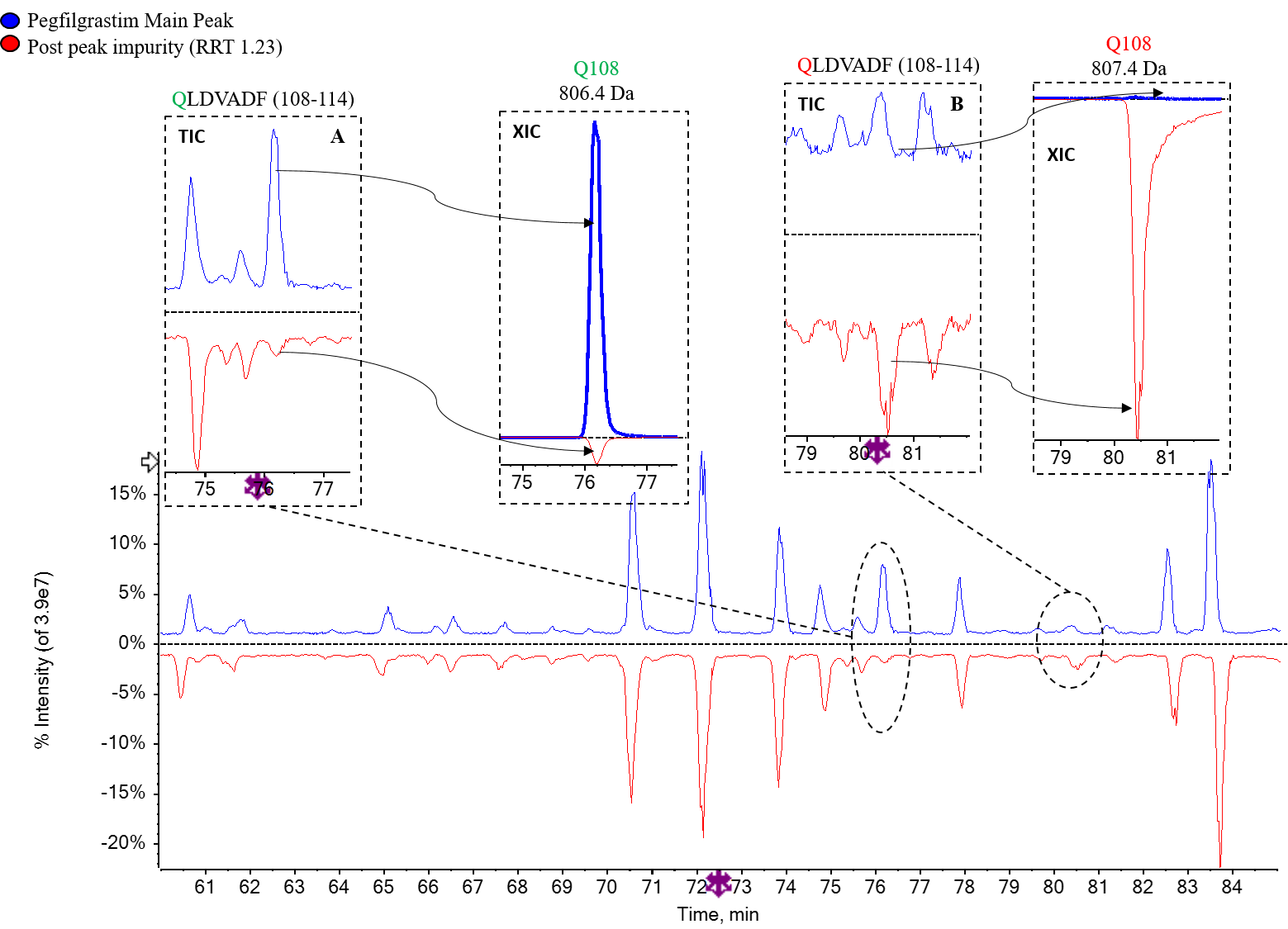

Supplement: S20 Fig — (TIF) [file pone.0212622.s020.tif]

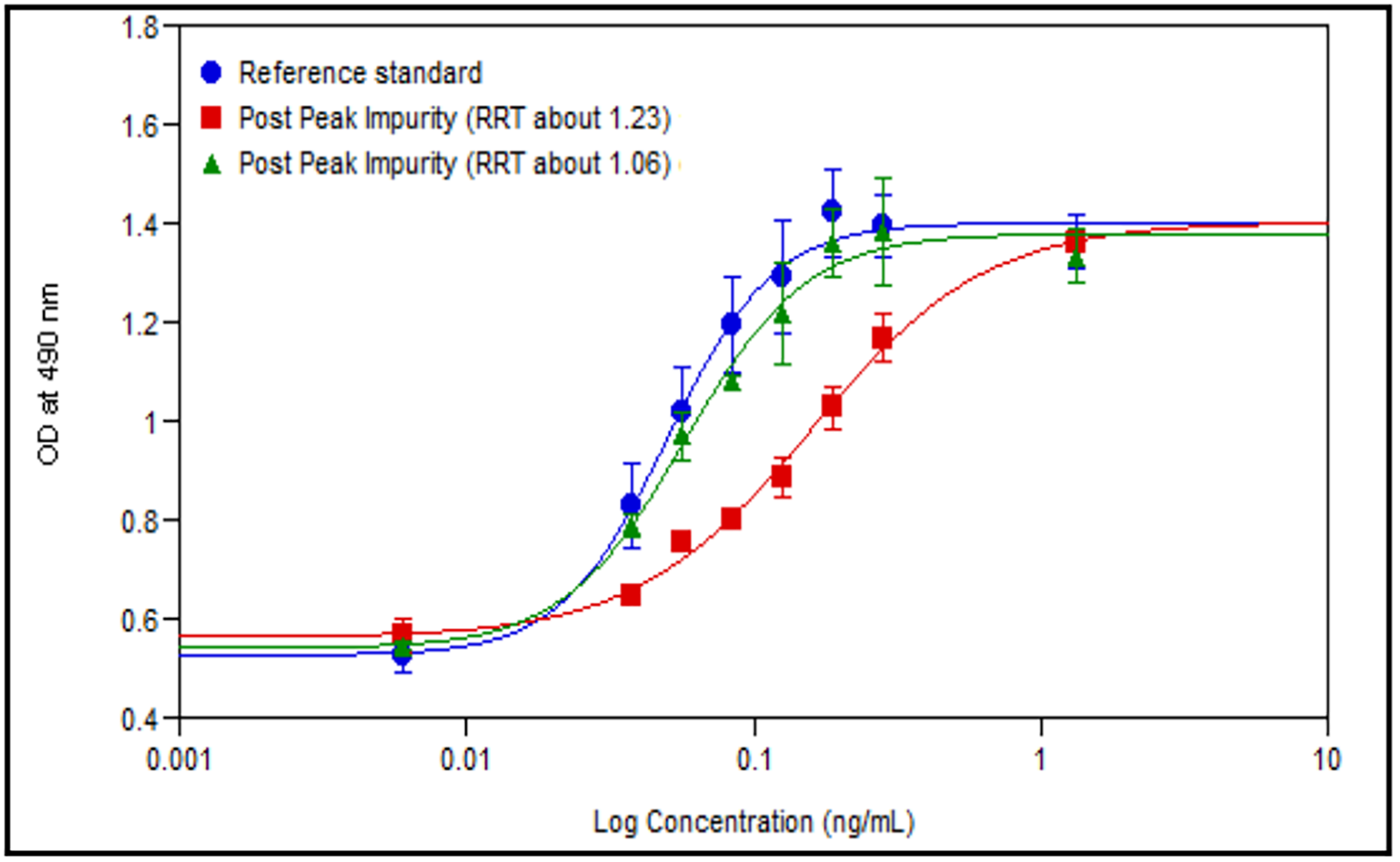

Supplement: S21 Fig — (TIF) [file pone.0212622.s021.tif]

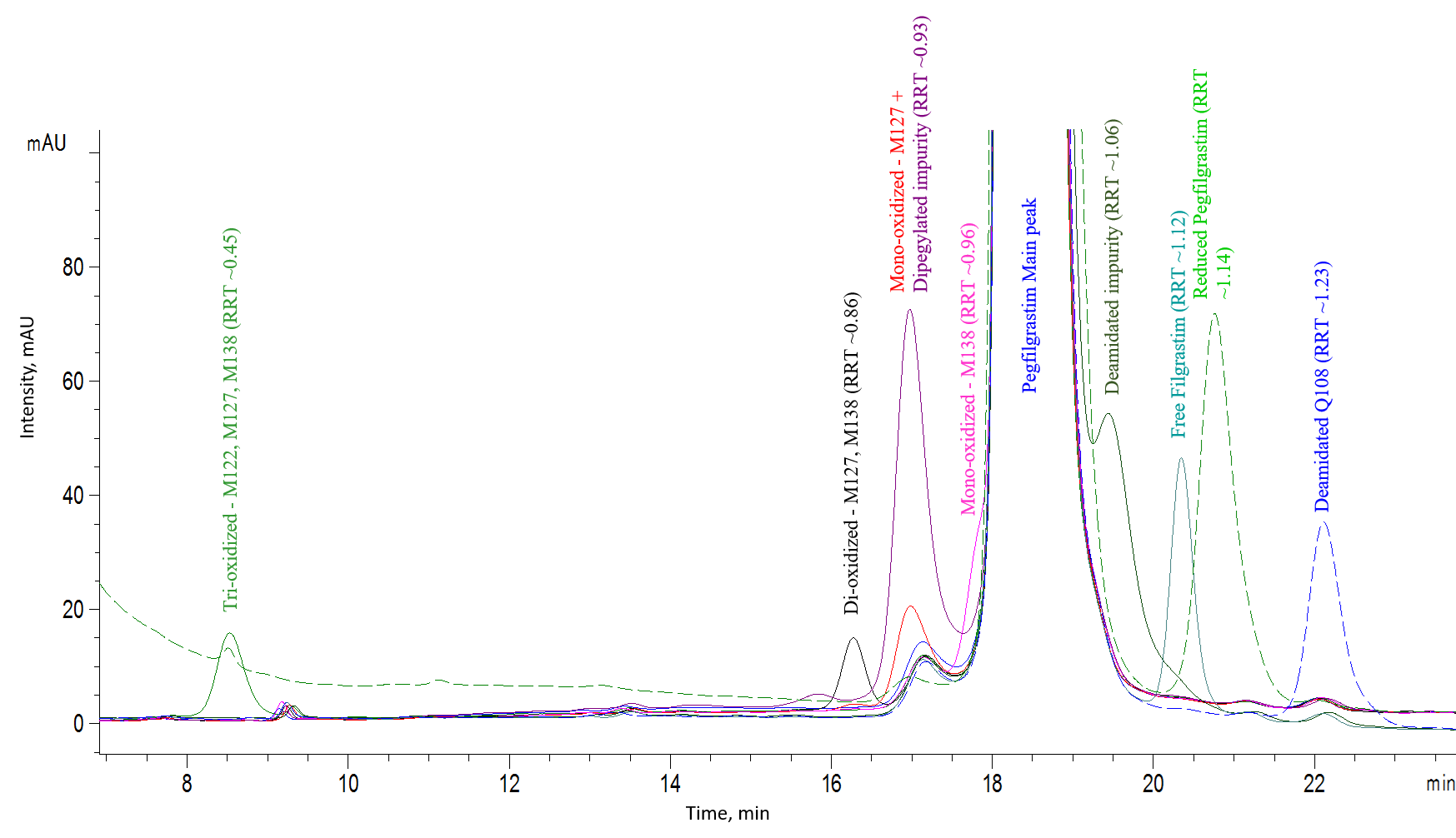

Supplement: S22 Fig — (TIF) [file pone.0212622.s022.tif]

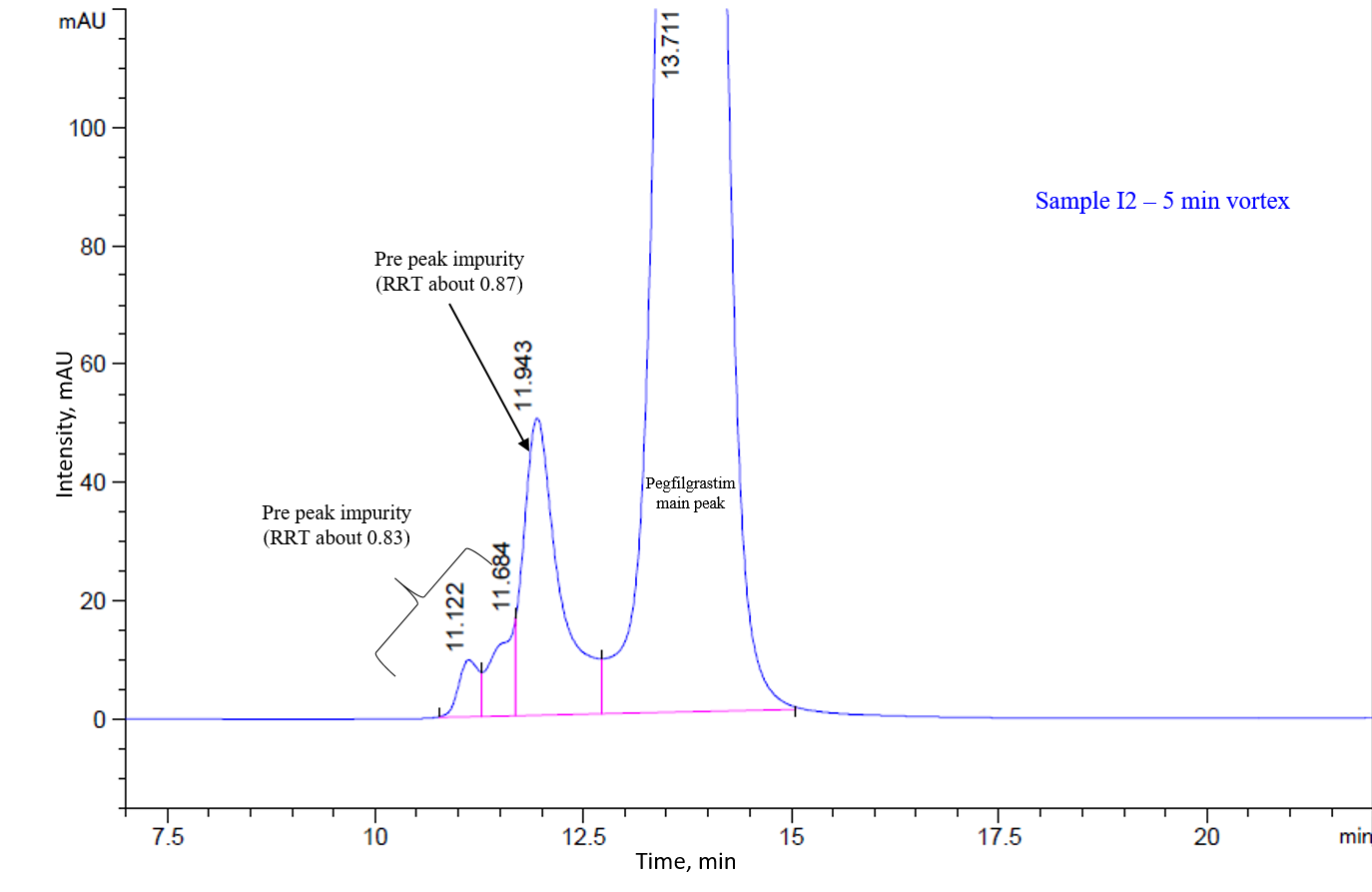

Supplement: S23 Fig — (TIF) [file pone.0212622.s023.tif]

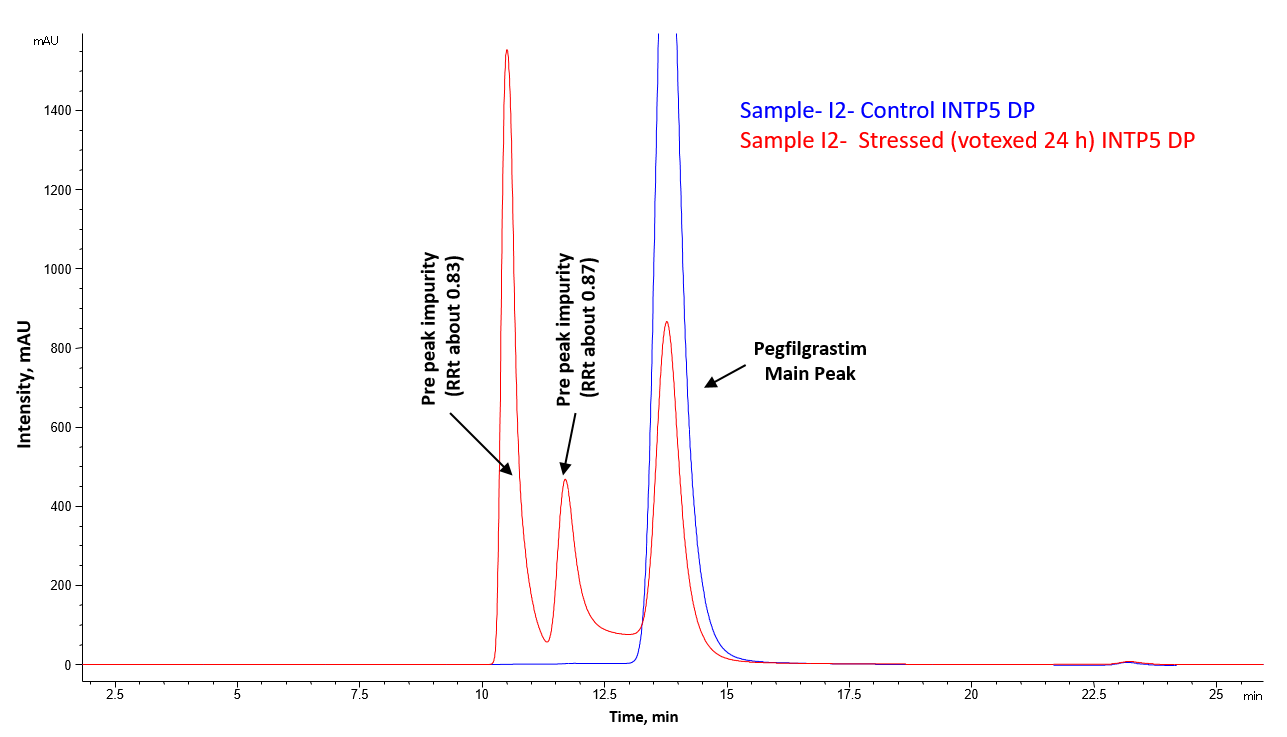

Supplement: S24 Fig — (TIF) [file pone.0212622.s024.tif]
