# Supplementary material for: Structural similarity, characterization of Poly Ethylene Glycol linkage and identification of product related variants in biosimilar pegfilgrastim
Source: PLoS One. 2019 Mar 13;14(3):e0212622. doi: 10.1371/journal.pone.0212622 (PMC6415886; doi:10.1371/journal.pone.0212622)
Supplement: S1 Appendix — (DOCX) [file pone.0212622.s025.docx]

**SUPPORTING INFORMATION**

**Structural similarity, characterization of Poly Ethylene Glycol linkage and identification of product related variants in biosimilar pegfilgrastim**

Rakesh Shekhawat^1^, Chintan Kumar Shah^1^, Akash Patel^1^, Sankaranarayanan Srinivasan^1^, Poonam Kapoor^2^ , Suvaskumar Patel^2^, Sharwan Kumar^1^, Sandeep Sonar^1^, Namrata More^1^, Manasvi Joshi^1^, Jatin Patel^1^, Milan Vachhani^2^, Bhargav Prasad Kodaganti^2^,  Upasana Choavatiya^2^,   Arabhi Pushpaja^2^, Shubhangi Argade^3^, Nidhi Nuwal^2^, Manish Kumar^2^ , Sridevi Khambhampaty^1,2,3^*

^1^Analytical Development Laboratory, Intas Pharmaceuticals Ltd. (Biopharma Division), Ahmedabad, Gujarat, India

^2^Biocharacterization Development Laboratory, Intas Pharmaceuticals Ltd. (Biopharma Division), Ahmedabad, Gujarat, India

^3^CMC Technical writing team, Intas Pharmaceuticals Ltd. (Biopharma Division), Ahmedabad, Gujarat, India

*Corresponding Author

Email: [Sridevi_K@intaspharma.com](mailto:Sridevi_K@intaspharma.com) (SK)

##

## Table A: N-terminal sequence analysis of INTP5 and EU sourced Neulasta^®^, US sourced Neulasta^®^

| **Amino Acid Number** | **1** | **2** | **3** | **4** | **5** | **6** | **7** | **8** | **9** | **10** | **11** | **12** | **13** | **14** | **15** | **16** | **17** | **18** | **19** | **20** |
| --- | --- | --- | --- | --- | --- | --- | --- | --- | --- | --- | --- | --- | --- | --- | --- | --- | --- | --- | --- | --- |
| **INTP5**  **(for 2 lots)** | ***** | **T** | **P** | **L** | **G** | **P** | **A** | **S** | **S** | **L** | **P** | **Q** | **S** | **F** | **L** | **L** | **K** | **C^#^** | **L** | **E** |
|  | ***** | **T** | **P** | **L** | **G** | **P** | **A** | **S** | **S** | **L** | **P** | **Q** | **S** | **F** | **L** | **L** | **K** | **C^#^** | **L** | **E** |
| **EU Neulasta^®^ (for 2 lots)** | ***** | **T** | **P** | **L** | **G** | **P** | **A** | **S** | **S** | **L** | **P** | **Q** | **S** | **F** | **L** | **L** | **K** | **C^#^** | **L** | **E** |
|  | ***** | **T** | **P** | **L** | **G** | **P** | **A** | **S** | **S** | **L** | **P** | **Q** | **S** | **F** | **L** | **L** | **K** | **C^#^** | **L** | **E** |
| **US Neulasta^®^ (for 2 lots)** | ***** | **T** | **P** | **L** | **G** | **P** | **A** | **S** | **S** | **L** | **P** | **Q** | **S** | **F** | **L** | **L** | **K** | **C^#^** | **L** | **E** |
|  | ***** | **T** | **P** | **L** | **G** | **P** | **A** | **S** | **S** | **L** | **P** | **Q** | **S** | **F** | **L** | **L** | **K** | **C^#^** | **L** | **E** |

**^*: As N-terminal methionine is pegylated and so cannot be detected by Edman degradation^**

**^#: Cysteine cannot be recovered by PPSQ-51A N-terminal sequencer using normal analysis technique and is inferred based on peptide map LC-MS data^**

## Table B: Data of peptide mapping by LC-ESI-MS/MS

| **Identification of peptides** | | **INTP5** | | | **EU Neulasta^®^** | | | **US Neulasta^®^** | | | |
| --- | --- | --- | --- | --- | --- | --- | --- | --- | --- | --- | --- |
|  |  | **I5** | **I1** | **I6** | **E4** | **E1** | **U2** | **U6** | **U7** | **U1** | **U5** |
| **Peptide Name** | **Theoretical Mass (Da)** | **Experimental Mass (Da)** | | | | | | | | | |
| G1_MTPLGPASSLPQSFLLKCLE | 2131.1 | NA^#^ | NA^#^ | NA^#^ | NA^#^ | NA^#^ | NA^#^ | NA^#^ | NA^#^ | NA^#^ | NA^#^ |
| G1_MC_SFLLKCLE | 951.5 | 951.5 | 951.5 | 951.5 | 951.5 | 951.5 | 951.5 | 951.5 | 951.5 | 951.5 | 951.5 |
| G2-G3_QVRKIQGDGAALQE | 1511.8 | 1511.8 | 1511.8 | 1511.8 | 1511.8 | 1511.8 | 1511.8 | 1511.8 | 1511.8 | 1511.8 | 1511.8 |
| G4-G5_KLCATYKLCHPEE | 1533.7 | 1533.7 | 1533.7 | 1533.7 | 1533.7 | 1533.7 | 1533.7 | 1533.7 | 1533.7 | 1533.7 | 1533.7 |
| G6_LVLLGHSLGIPWAPLSSCPSQALQLAGCLSQLHSGLFLYQGLLQALE | 4941.6 | 4941.7 | 4941.6 | 4941.6 | 4941.6 | 4941.7 | 4941.7 | 4941.6 | 4941.6 | 4941.6 | 4941.6 |
| G7_GISPE | 501.2 | 501.2 | 501.2 | 501.2 | 501.2 | 501.2 | 501.2 | 501.2 | 501.2 | 501.2 | 501.2 |
| G8_LGPTLD | 614.3 | 614.3 | 614.3 | 614.3 | 614.3 | 614.3 | 614.3 | 614.3 | 614.3 | 614.3 | 614.3 |
| G9_TLQLD | 588.3 | 588.3 | 588.3 | 588.3 | 588.3 | 588.3 | 588.3 | 588.3 | 588.3 | 588.3 | 588.3 |
| G10-G12_VADFATTIWQQMEE | 1667.8 | 1667.8 | 1667.8 | 1667.8 | 1667.8 | 1667.8 | 1667.8 | 1667.8 | 1667.8 | 1667.8 | 1667.8 |
| G13_LGMAPALQPTQGAMPAFASAFQRRAGGVLVASHLQSFLE | 4025.1 | 4025.1 | 4025.1 | 4025.1 | 4025.1 | 4025.1 | 4025.1 | 4025.1 | 4025.1 | 4025.1 | 4025.1 |
| G14_VSYRVLRHLAQP | 1437.8 | 1437.8 | 1437.8 | 1437.8 | 1437.8 | 1437.8 | 1437.8 | 1437.8 | 1437.8 | 1437.8 | 1437.8 |

Table C: Results of Far UV CD

| **Sample Name** | **Minima 1**  **Wavelength [nm]** | **Minima 2**  **Wavelength [nm]** |
| --- | --- | --- |
| INTP5  (N=3) | 221.6 | 209.4 |
|  | 221.8 | 209.4 |
|  | 222.0 | 209.2 |
| Average | 221.8 | 209.3 |
| SD | 0.2 | 0.1 |
| % RSD | 0.1 | 0.1 |
| US Neulasta^®^  (N=4) | 221.0 | 209.2 |
|  | 221.6 | 209.2 |
|  | 221.8 | 209.0 |
|  | 221.6 | 209.6 |
| Average | 221.5 | 209.3 |
| SD | 0.3 | 0.3 |
| % RSD | 0.2 | 0.1 |
| EU Neulasta^®^  (N=6) | 221.8 | 209.0 |
|  | 222.2 | 209.4 |
|  | 221.6 | 209.6 |
|  | 221.6 | 209.4 |
|  | 221.8 | 209.0 |
|  | 221.8 | 209.6 |
| Average | 221.8 | 209.3 |
| SD | 0.2 | 0.3 |
| % RSD | 0.1 | 0.1 |
| AUS Neulasta^®^  (N=3) | 222.2 | 209.0 |
|  | 221.8 | 209.8 |
|  | 221.8 | 209.2 |
| Average | 221.9 | 209.3 |
| SD | 0.2 | 0.4 |
| % RSD | 0.1 | 0.2 |

Table D: Results of Intrinsic fluorescence for excitation at 291 nm

| **Sample Name** | **Intensity at 345 nm**  **(x10^7^cps)** |
| --- | --- |
| INTP5 (N=3) | 5.89 |
|  | 6.03 |
|  | 5.76 |
| Average | 5.89 |
| SD | 0.14 |
| % RSD | 2.29 |
| US Neulasta^®^ (N=4) | 5.87 |
|  | 5.63 |
|  | 5.61 |
|  | 5.86 |
| Average | 5.74 |
| SD | 0.14 |
| % RSD | 2.47 |
| EU Neulasta^®^ (N=2) | 5.89 |
|  | 5.68 |
| Average | 5.79 |
| SD | 0.15 |
| % RSD | 2.57 |
| AUS Neulasta^®^(N=3) | 5.89 |
|  | 5.86 |
|  | 5.69 |
| Average | 5.81 |
| SD | 0.11 |
| % RSD | 1.86 |

**Table E: Chemical shift and weighted sum distribution of 2D ^1^H-^13^ C HSQC spectra peaks in the aliphatic region**

| **Sample - I1** | | | | | **Sample - I2** | | | | | **Sample- E3** | | | | |
| --- | --- | --- | --- | --- | --- | --- | --- | --- | --- | --- | --- | --- | --- | --- |
| **^13^C** | **^1^H** | **Weight sum** | **intensity** | **Sum x int** | **^13^C** | **^1^H** | **Weight**  **sum** | **intensity** | **Sum x int** | **^13^C** | **^1^H** | **Weight**  **sum** | **intensity** | **Sum x int** |
| 13.224 | 0.897 | 22.195 | 69606 | 1544898 | 13.224 | 0.893 | 22.151 | 64503 | 1428800 | 13.224 | 0.902 | 22.239 | 81032 | 1802063 |
| 14.114 | -0.027 | 13.846 | 69847 | 967123 | 14.114 | -0.036 | 13.758 | 55681 | 766076 | 14.114 | -0.022 | 13.890 | 61619 | 855906 |
| 18.566 | -0.014 | 18.430 | 83888 | 1546081 | 18.566 | -0.009 | 18.474 | 68519 | 1265841 | 18.566 | -0.014 | 18.430 | 66372 | 1223256 |
| 15.302 | 0.668 | 21.985 | 87453 | 1922610 | 15.302 | 0.664 | 21.941 | 48565 | 1065540 | 15.302 | 0.668 | 21.985 | 76651 | 1685134 |
| 18.863 | 0.391 | 22.774 | 87884 | 2001479 | 18.863 | 0.391 | 22.774 | 82235 | 1872828 | 18.863 | 0.391 | 22.774 | 87477 | 1992210 |
| 12.630 | 0.814 | 20.765 | 89240 | 1853095 | 12.630 | 0.814 | 20.765 | 83795 | 1740028 | 12.630 | 0.822 | 20.853 | 99833 | 2081847 |
| 15.895 | 0.800 | 23.898 | 94870 | 2267213 | 15.895 | 0.805 | 23.942 | 87577 | 2096777 | 15.895 | 0.800 | 23.898 | 102707 | 2454502 |
| 21.238 | 1.100 | 32.233 | 96029 | 3095255 | 21.238 | 1.104 | 32.277 | 89708 | 2895460 | 21.238 | 1.100 | 32.233 | 92962 | 2996398 |
| 21.534 | 0.343 | 24.961 | 104849 | 2617167 | 21.534 | 0.338 | 24.917 | 103240 | 2572462 | 21.534 | 0.343 | 24.961 | 99719 | 2489116 |
| 22.128 | 1.012 | 32.243 | 112352 | 3622554 | 22.128 | 1.012 | 32.243 | 104170 | 3358743 | 22.128 | 1.016 | 32.287 | 119989 | 3874073 |
| 10.850 | 0.748 | 18.325 | 113238 | 2075030 | 10.850 | 0.752 | 18.369 | 86344 | 1586010 | 10.850 | 0.752 | 18.369 | 100977 | 1854796 |
| 21.534 | 1.034 | 31.869 | 114223 | 3640207 | 21.534 | 1.029 | 31.825 | 101174 | 3219893 | 21.534 | 1.034 | 31.869 | 114832 | 3659615 |
| 16.192 | 1.029 | 26.483 | 114446 | 3030862 | 16.192 | 1.029 | 26.483 | 101193 | 2679884 | 16.192 | 1.034 | 26.527 | 112721 | 2990139 |
| 24.502 | 0.827 | 32.769 | 123954 | 4061886 | 24.502 | 0.827 | 32.769 | 120238 | 3940115 | 24.502 | 0.827 | 32.769 | 114515 | 3752576 |
| 17.973 | 0.303 | 21.004 | 124989 | 2625231 | 17.973 | 0.308 | 21.048 | 123060 | 2590130 | 17.973 | 0.308 | 21.048 | 131731 | 2772635 |
| 17.676 | 0.875 | 26.427 | 130066 | 3437241 | 17.676 | 0.871 | 26.383 | 127184 | 3355483 | 17.676 | 0.871 | 26.383 | 131405 | 3466845 |
| 15.005 | 0.541 | 20.412 | 132544 | 2705448 | 15.005 | 0.541 | 20.412 | 116954 | 2387230 | 15.005 | 0.536 | 20.368 | 134477 | 2738987 |
| 9.959 | 0.497 | 14.926 | 133858 | 1997965 | 9.959 | 0.497 | 14.926 | 119082 | 1777418 | 9.959 | 0.497 | 14.926 | 119782 | 1787866 |
| 22.425 | 0.387 | 26.292 | 167209 | 4396209 | 22.425 | 0.391 | 26.336 | 150219 | 3956123 | 22.425 | 0.391 | 26.336 | 150941 | 3975137 |
| 19.457 | 0.844 | 27.900 | 178238 | 4972787 | 19.457 | 0.844 | 27.900 | 172577 | 4814847 | 19.457 | 0.844 | 27.900 | 180342 | 5031488 |
| 23.909 | 0.910 | 33.012 | 179340 | 5920318 | 23.909 | 0.906 | 32.968 | 168117 | 5542431 | 23.909 | 0.906 | 32.968 | 170265 | 5613245 |
| 20.050 | 0.673 | 26.777 | 184909 | 4951364 | 20.050 | 0.677 | 26.821 | 177447 | 4759359 | 20.050 | 0.677 | 26.821 | 176932 | 4745546 |
| 16.786 | 1.117 | 27.957 | 195800 | 5473883 | 16.786 | 1.117 | 27.957 | 189172 | 5288587 | 16.786 | 1.117 | 27.957 | 200947 | 5617775 |
| 18.863 | 0.677 | 25.634 | 207671 | 5323459 | 18.863 | 0.682 | 25.678 | 197818 | 5079590 | 18.863 | 0.682 | 25.678 | 209149 | 5370549 |
| 19.160 | 0.726 | 26.415 | 218689 | 5776648 | 19.160 | 0.726 | 26.415 | 209384 | 5530857 | 19.160 | 0.721 | 26.371 | 215585 | 5685170 |
| 19.754 | 0.752 | 27.273 | 258685 | 7054987 | 19.754 | 0.752 | 27.273 | 245116 | 6684926 | 19.754 | 0.752 | 27.273 | 267076 | 7283830 |
| 21.534 | 0.959 | 31.121 | 260386 | 8103551 | 21.534 | 0.959 | 31.121 | 245447 | 7638630 | 21.534 | 0.959 | 31.121 | 246912 | 7684222 |
| 22.722 | 0.585 | 28.569 | 269751 | 7706381 | 22.722 | 0.585 | 28.569 | 248406 | 7096587 | 22.722 | 0.585 | 28.569 | 260002 | 7427867 |
| 21.238 | 0.651 | 27.745 | 283771 | 7873085 | 21.238 | 0.655 | 27.789 | 256162 | 7118358 | 21.238 | 0.655 | 27.789 | 266595 | 7408275 |
| 18.270 | 0.937 | 27.637 | 307116 | 8487611 | 18.270 | 0.937 | 27.637 | 278483 | 7696295 | 18.270 | 0.937 | 27.637 | 296753 | 8201214 |
| 23.612 | 0.690 | 30.515 | 315979 | 9642068 | 23.612 | 0.690 | 30.515 | 293340 | 8951241 | 23.612 | 0.690 | 30.515 | 303741 | 9268626 |
| 23.909 | 0.611 | 30.020 | 360015 | 10807542 | 23.909 | 0.611 | 30.020 | 337400 | 10128647 | 23.909 | 0.611 | 30.020 | 359757 | 10799797 |
| 21.238 | 0.699 | 28.229 | 435633 | 12297266 | 21.238 | 0.699 | 28.229 | 411262 | 11609309 | 21.238 | 0.704 | 28.273 | 443586 | 12541285 |
| 19.160 | 1.034 | 29.495 | 444539 | 13111633 | 19.160 | 1.034 | 29.495 | 405623 | 11963810 | 19.160 | 1.038 | 29.539 | 438426 | 12950622 |
| 22.722 | 0.756 | 30.285 | 456787 | 13833566 | 22.722 | 0.756 | 30.285 | 423567 | 12827515 | 22.722 | 0.761 | 30.329 | 453912 | 13766470 |
| 18.863 | 1.113 | 29.990 | 458691 | 13756189 | 18.863 | 1.113 | 29.990 | 431895 | 12952574 | 18.863 | 1.113 | 29.990 | 456179 | 13680854 |
| 22.128 | 0.726 | 29.383 | 550882 | 16186511 | 22.128 | 0.726 | 29.383 | 503433 | 14792321 | 22.128 | 0.726 | 29.383 | 538061 | 15809793 |
| 20.941 | 0.805 | 28.988 | 768986 | 22291135 | 20.941 | 0.805 | 28.988 | 713344 | 20678202 | 20.941 | 0.809 | 29.032 | 748095 | 21718470 |
| 22.425 | 0.849 | 30.912 | 961449 | 29720023 | 22.425 | 0.849 | 30.912 | 892792 | 27597718 | 22.425 | 0.849 | 30.912 | 949444 | 29348928 |
| 17.379 | 1.284 | 30.222 | 108212 | 3270394 | 17.379 | 1.284 | 30.222 | 105320 | 3182992 | 17.379 | 1.280 | 30.178 | 97803 | 2951509 |
| 19.754 | 1.421 | 33.961 | 113313 | 3848166 | 19.754 | 1.425 | 34.005 | 112296 | 3818569 | 19.754 | 1.425 | 34.005 | 120591 | 4100637 |
| 15.302 | 1.614 | 31.445 | 126956 | 3992068 | 15.302 | 1.614 | 31.445 | 118948 | 3740260 | 15.302 | 1.614 | 31.445 | 137505 | 4323776 |
| 13.818 | 1.733 | 31.149 | 148551 | 4627141 | 13.818 | 1.738 | 31.193 | 132033 | 4118439 | 13.818 | 1.738 | 31.193 | 138506 | 4320348 |
| 15.005 | 1.262 | 27.628 | 158720 | 4385069 | 15.005 | 1.262 | 27.628 | 172887 | 4776470 | 15.005 | 1.262 | 27.628 | 154925 | 4280221 |
| 19.457 | 1.315 | 32.608 | 177704 | 5794519 | 19.457 | 1.315 | 32.608 | 156475 | 5102290 | 19.457 | 1.315 | 32.608 | 173027 | 5642013 |
| 15.895 | 1.843 | 34.326 | 188226 | 6461064 | 15.895 | 1.839 | 34.282 | 166240 | 5699056 | 15.895 | 1.843 | 34.326 | 196592 | 6748237 |
| 17.379 | 1.355 | 30.926 | 198609 | 6142202 | 17.379 | 1.355 | 30.926 | 183727 | 5681960 | 17.379 | 1.355 | 30.926 | 198728 | 6145882 |
| 15.302 | 1.892 | 34.217 | 198917 | 6806244 | 15.302 | 1.892 | 34.217 | 177624 | 6077672 | 15.302 | 1.892 | 34.217 | 195930 | 6704039 |
| 15.005 | 1.460 | 29.608 | 216842 | 6420193 | 15.005 | 1.460 | 29.608 | 201362 | 5961866 | 15.005 | 1.460 | 29.608 | 224384 | 6643494 |
| 16.192 | 1.170 | 27.891 | 261871 | 7303818 | 16.192 | 1.166 | 27.847 | 229511 | 6391170 | 16.192 | 1.170 | 27.891 | 250981 | 7000086 |
| 15.005 | 1.350 | 28.508 | 280112 | 7985349 | 15.005 | 1.350 | 28.508 | 252146 | 7188103 | 15.005 | 1.350 | 28.508 | 266035 | 7584046 |
| 14.114 | 2.002 | 34.129 | 302581 | 10326878 | 14.114 | 2.002 | 34.129 | 290077 | 9900125 | 14.114 | 2.002 | 34.129 | 308312 | 10522473 |
| 16.489 | 1.298 | 29.464 | 385029 | 11344379 | 16.489 | 1.298 | 29.464 | 341537 | 10062944 | 16.489 | 1.298 | 29.464 | 377611 | 11125817 |
| 18.863 | 1.196 | 30.826 | 458561 | 14135647 | 18.863 | 1.201 | 30.870 | 444986 | 13736762 | 18.863 | 1.201 | 30.870 | 453658 | 14004468 |
| 16.489 | 1.342 | 29.904 | 483200 | 14449468 | 16.489 | 1.342 | 29.904 | 463561 | 13862189 | 16.489 | 1.342 | 29.904 | 483541 | 14459665 |

## Table F: Pairwise linear correlation for methyl group chemical shifts (above diagonal) and products of shifts and intensities (below diagonal)

|  | **I1** | **I2** | **E3** |
| --- | --- | --- | --- |
| **I1** | - | 0.9996 | 1.0010 |
| **I2** | 1.0690 | - | 1.0014 |
| **E3** | 1.0119 | 0.9451 | - |

## Table G: Results of Tm of Pegfilgrastim samples by DSC

| Sample Name | INTP5  (n= 16) | US Neulasta^®^ (n=11) | EU Neulasta^®^ (n=15) |
| --- | --- | --- | --- |
| Average Tm | 68.84 | 68.58 | 68.63 |
| SD | 0.22 | 0.30 | 0.18 |
| Range | 68.59 - 69.18 | 68.16 - 69.12 | 68.16 - 68.88 |

## Table H: Summary of characterization of oxidized peaks in Oxidized (24 hours) INTP5 (Sample I2)

| **Oxidized impurity** | **Peptide** | **Sequence** | **Theoretical mass (Da)** | **Experimental mass (Da)** | **Mass difference** | **Identified as** |
| --- | --- | --- | --- | --- | --- | --- |
| Oxidized impurity 1 | G10-12  (111- 124) | VADFATTIWQQMEE | 1667.8 | 1667.8 | +16 | Met^122^,  Met^127^ and Met^138^ |
|  |  | VADFATTIWQQM[Oxi]EE | 1683.7 | 1683.8 |  |  |
|  | G13  (125-163) | LGMAPALQPTQGAMPAFASAFQRRAGGVLVASHLQSFLE | 4025.1 | 4025.1 | +32 |  |
|  |  | LGM[Oxi]APALQPTQGAM[Oxi]PAFASAFQRRAGGVLVASHLQSFLE | 4057.1 | 4057.1 |  |  |
| Oxidized impurity 2 | G13  (125-163) | LGMAPALQPTQGAMPAFASAFQRRAGGVLVASHLQSFLE | 4025.1 | 4025.1 | +32 | Met^127^ and Met^138^ |
|  |  | LGM[Oxi]APALQPTQGAM[Oxi]PAFASAFQRRAGGVLVASHLQSFLE | 4057.1 | 4057.1 |  |  |
| Oxidized impurity 3 | G13  (125-163) | LGMAPALQPTQGAMPAFASAFQRRAGGVLVASHLQSFLE | 4025.1 | 4025.1 | +16 | Met^127^ (Major) |
|  |  | LGM[Oxi]APALQPTQGAMPAFASAFQRRAGGVLVASHLQSFLE | 4041.1 | 4041.1 |  |  |
|  |  | LGM[Oxi]APALQPTQGAM[Oxi]PAFASAFQRRAGGVLVASHLQSFLE | 4057.1 | 4057.1 | +32 | Met^127^ and Met^138^  (Minor) |
| Oxidized impurity 4 | G13  (125-163) | LGMAPALQPTQGAMPAFASAFQRRAGGVLVASHLQSFLE | 4025.1 | 4025.1 | +16 | Met^138^ |
|  |  | LGMAPALQPTQGAM[Oxi]PAFASAFQRRAGGVLVASHLQSFLE | 4041.1 | 4041.1 |  |  |
| INTP5 control | G10-12 | VADFATTIWQQMEE | 1667.8 | 1667.8 | 0 | Native |
|  | G13 | LGMAPALQPTQGAMPAFASAFQRRAGGVLVASHLQSFLE | 4025.1 | 4025.1 | 0 | Native |

## Table I: Relative % deamidation in Post peak impurities RRT 1.06 and RRT 1.23 with respect to Pegfilgrastim main peak and INTP5 control

| **Position of glutamine** | **INTP5 control DP (Sample I2)** | **Pegfilgrastim Main peak** | **Post peak impurity (RRT ~ 1.06)** | **Post peak impurity**  **(RRT ~ 1.23)** |
| --- | --- | --- | --- | --- |
| **Q12** | 0.05 | *1.79* | *4.58* | *2.70* |
| **Q21 / Q26 / Q33** | 0.24 | 0.96 | **4.61** | 1.97 |
| **Q68** | 0.02 | *0.91* | *1.64* | *1.23* |
| **Q71** | 0.19 | *1.41* | *3.20* | *2.03* |
| **Q78** | 0.03 | 0.06 | 0.13 | 0.15 |
| **Q87** | 0.04 | 0.18 | **6.83** | 0.68 |
| **Q91** | 0.00 | 0.00 | **9.53** | 0.97 |
| **Q108** | 0.00 | 0.00 | 0.00 | **56.67** |
| **Q120 / Q121** | 0.14 | 0.42 | **14.24** | 1.74 |
| **Q132 / Q135** | 0.19 | *4.72* | **9.46** | *5.91* |
| **Q146** | 0.05 | 0.34 | 3.31 | 1.00 |
| **Q159** | 0.01 | 0.05 | 0.58 | 0.21 |
| **Q174** | 0.18 | *6.18* | *5.36* | *6.10* |

## Table J: Integration *for* SEC-HPLC of Vortexed INTP5 (Sample I2)

| **Sr. No.** | **Time** | **Area % of Control** | **Area % of Vortexed** | **Calculated RRT** | **Remarks** |
| --- | --- | --- | --- | --- | --- |
| 1 | 11.1 | 0.2 | **0.9** | 0.81 | Pre peak impurity (RRT about 0.83) |
| 2 | 11.7 | ND* | **1.6** | 0.85 |  |
| 3 | 11.9 | **0.5** | **8.7** | 0.87 | Pre peak impurity (RRT about 0.87) |
| 4 | 13.7 | 99.2 | 88.7 | NA | Pegfilgrastim main peak |
| 5 | 20.2 | 0.1 | ND* | 1.45 | Free filgrastim |

* ND- not detected

## Table K: Summary of impurity characterization by various HPLC methods

| **Characterization of charge variants** | | | |
| --- | --- | --- | --- |
| Charge variants (Pegylation Reaction Output) observed in  **CEX-HPLC** | **Sample Name** | **Molecular characteristics** | **% Relative potency** |
|  | Pre Peak impurity 1  (RRT 0.60) | Dipegylated pegfilgrastim variant (Met1 and Lys41) | No dose response observed |
|  | Pre Peak impurity 2  (RRT 0.73) | Dipegylated pegfilgrastim variant (Met1 and Lys35) |  |
|  | Pre Peak impurity 3  (RRT 0.84) | Dipegylated pegfilgrastim variants and positional isomers of monopegylated filgrastim |  |
|  | Pegfilgrastim main peak | Native molecule | 90 |
| **Characterization of oxidized Species** | | | |
| Oxidized Species (Under stress Oxidation) observed in **RP-HPLC** | Control sample | Native protein with 3 non oxidised Met residues | 99 |
|  | Oxidized (24 hour oxidized) | Mixture of oxidised variants | 57 |
|  | Oxidized Impurity 1 | Variant at RRT 0.45 with oxidation at Met122, 127, 138 | No dose response observed |
|  | Oxidized Impurity 2 | Variant at RRT 0.86 with oxidation at Met127 and 138 | No dose response observed |
|  | Oxidized Impurity 3 | Variant at RRT 0.91 with oxidation at Met127 | Not evaluated; inferred to be inactive based on impurity 2 and 4 assessment. |
|  | Oxidized Impurity 4 | Variant at RRT 0.96 with oxidation at Met138 | 99 |
| **Characterization of deamidated Species** | | | |
| Deamidated Species (Under stress deamidation) observed in **RP-HPLC** | Pre-peak Impurity  (RRT about 0.93) | Mono-oxidation at M127 position and dipegylated pegfilgrastim impurities | No dose response observed |
|  | Post-peak Impurity  (RRT about 1.06) | Mono-deamidation of pegfilgrastim | 77 |
|  | Post-peak Impurity  (RRT about 1.12) | Free filgrastim and reduced pegfilgrastim | Not evaluated |
|  | Post-peak Impurity  (RRT about 1.23) | Deamidation at Q108 position | 27 |
